# Correction: Complete mitochondrial genome sequence of the “copper moss” *Mielichhoferia elongata* reveals independent *nad7* gene functionality loss

**DOI:** 10.7717/peerj.4350/correction-1

**Published:** 2018-09-18

**Authors:** Denis V. Goryunov, Svetlana V. Goryunova, Oxana I. Kuznetsova, Maria D. Logacheva, Irina A. Milyutina, Alina V. Fedorova, Michael S. Ignatov, Aleksey V. Troitsky

**Affiliations:** 1 Belozersky Institute of Physico-Chemical Biology, Lomonosov Moscow State University, Moscow, Russia; 2 Institute of General Genetics Russian Academy of Science, Moscow, Russia; 3 Tsitsin Main Botanical Garden Russian Academy of Science, Moscow, Russia

Corrigendum:

After publication, the authors noticed that the first author’s surname is spelled incorrectly. This has been corrected so that Denis V. Goruynov is now listed Denis V. Goryunov.

