# Supplementary material for: Complete mitochondrial genome sequence of the “copper moss” Mielichhoferia elongata reveals independent nad7 gene functionality loss
Source: PeerJ. 2018 Feb 2;6:e4350. doi: 10.7717/peerj.4350 (PMC5798402; doi:10.7717/peerj.4350)
Supplement: Supplemental Information 1 [file peerj-06-4350-s001.doc]

LOCUS Mielichhoferia 100342 bp DNA circular 01-JUL-2017

DEFINITION elongata mitochondrion complete genome, whole genome

shotgun sequence.

ACCESSION

VERSION

KEYWORDS WGS.

SOURCE Mielichhoferia elongata

ORGANISM Mielichhoferia elongata

Unclassified.

REFERENCE 1 (bases 1 to 100342)

AUTHORS Goryunov,D.V., Goryunova,S.V., Kuznetsova,O., Logacheva,M.D.,

Milyutina,I.A., Fedorova,A.V., Ignatov,M.S. and Troitsky,A.V.

TITLE The complete mitochondrial genome of copper moss Mielichhoferia

elongata

JOURNAL Unpublished

REFERENCE 2 (bases 1 to 100342)

AUTHORS Goryunov,D.V., Goryunova,S.V., Kuznetsova,O., Logacheva,M.D.,

Milyutina,I.A., Fedorova,A.V., Ignatov,M.S. and Troitsky,A.V.

TITLE Direct Submission

JOURNAL Submitted (01-JUL-2017) Evolutionary Biochemistry, A.N. Belozersky

Institute Of Physico-Chemical Biology MSU, Leninskye gory, Moscow

119992, Russian Federation

FEATURES Location/Qualifiers

source 1..100342

/organism="Mielichhoferia elongata"

/mol_type="genomic DNA"

gene 48..121

/gene="trnI (CAU)"

tRNA 48..121

/gene="trnI (CAU)"

/product="tRNA-Ile"

gene 296..368

/gene="trnK (UUU)"

tRNA 296..368

/gene="trnK (UUU)"

/product="tRNA-Lys"

gene complement(1272..9517)

/gene="cox1"

CDS complement(join(1272..1776,3812..4143,6771..6878,

7269..7381,9007..9517))

/gene="cox1"

/codon_start=1

/product="cytochrome c oxidase subunit 1"

/translation="MNNFAQRWLFSTNHKDIGTLYCIFGAIAGVMGTCFSVLIRMELA

QPGNQILGGNHQLYNVLITAHAFLMISFMVMPAMIGGFGNWFVPILIGAPDMAFPRLN

NISFWLLPPSLLLLLSSALVEVGAGTGWTVYPPLSGITSHSGGSVDLAIFSLHLSGVS

SILGSINFITTIFNMRGPGMTMHRLPLFVWSVLVTAFLLLLSLPVLAGAITMLLTDRN

FNTTFFDPAGGGDPILYQHLFWFFGHPEVYILILPGFGIISHIVSTFSRKPVFGYLGM

VYALISIGVLGFIVWAHHMFTVGLDVDTRAYFTAATMIIAVPTGIKIFSWIATMWGGS

IQYKTPMLFAVGFIFLFTVGGLTGIVLANSGLDIALHDTYYVVAHFHYVLSMGAVFAL

FAGFYYWIGKITGLQYPETLGQIHFWITFFGVNLTFFPMHFLGLAGMPRRIPDYPDAY

AGWNAFSSFGSYVSVVGILCFFVVVFLTLTSENKCASSPWAVEQNSTTLEWMVKSPPA

FHTFSELPVIKESI"

exon complement(1272..1776)

/gene="cox1"

/number=5

intron complement(1777..3811)

/gene="cox1"

/number=4

exon complement(3812..4143)

/gene="cox1"

/number=4

intron complement(4144..6770)

/gene="cox1"

/number=3

exon complement(6771..6878)

/gene="cox1"

/number=3

intron complement(6879..7268)

/gene="cox1"

/number=2

exon complement(7269..7381)

/gene="cox1"

/number=2

intron complement(7382..9006)

/gene="cox1"

/number=1

exon complement(9007..9517)

/gene="cox1"

/number=1

gene complement(9514..10065)

/gene="atp4"

CDS complement(9514..10065)

/gene="atp4"

/codon_start=1

/product="ATPase subunit 4"

/translation="MREFIIFAILIFSVLSSKQILIYNEEIIVALSFVGFVIFSQRTF

GKTLKATSDARSEALLSELQQLMSSQEALLSELKKQHELRSISLRSSTQMIGESCIND

LLTRCAPKCKQTVQAVLCQQVEQKLKTLLAIQEHSRSSLQEKIVTCFRETVCDEFRFS

KLRKHQSKLVQQSMVLLKDGVLK"

gene complement(10058..10582)

/gene="atp8"

CDS complement(10058..10582)

/gene="atp8"

/codon_start=1

/product="ATPase subunit 8"

/translation="MPQLDQFTYLTQFVWLCVFYMVFYVLLYNDGLPKISRILKLRKQ

LISHQNVGTDPSDYSVEQDVVFKECFDTSISYLYSGVSGASKWCNKMVKSLNANQLKR

LNKSYVCSLGEISVSQVIKKNALSIMSPSTYHITSLASRQTTALNKIYVLRGQRNTLV

NIKNGPGKKKNTNA"

gene complement(11136..11870)

/gene="rps1"

CDS complement(11136..11870)

/gene="rps1"

/codon_start=1

/product="ribosomal protein S1"

/translation="MSFTQLFSQYNSSLNLLRGSAIQCSVKKLRQNMILVNTGLKTPI

ICFQHELKRVPITKQTRFILGIEDVEVFGEPKMLLPKPLERKWKSKLVWTELTKIWRS

DRNLIKGFILNSVKGGYAVAIAGHIAFLPKSLRRNRKVFHSQWRIFSILNMNSKIGNI

VVKEIGDGKLDYSSPAKPRQKQVKRLGAKRKHLQNTKKNTFFHKYEETKKKKNKNSSF

IPSYGLYDPKDQTKLKALRPKASSPH"

gene 12279..12349

/gene="trnC (GCA)"

tRNA 12279..12349

/gene="trnC (GCA)"

/product="tRNA-Cys"

gene complement(12703..12776)

/gene="trnF (GAA)"

tRNA complement(12703..12776)

/gene="trnF (GAA)"

/product="tRNA-Phe"

gene 12798..13523

/gene="rps2"

CDS 12798..13523

/gene="rps2"

/codon_start=1

/product="ribosomal protein S2"

/translation="MYNSSLLVIQKLLSTNAYLGHRIPTSDFQGYLYGFRNEMAIIDL

EKTLICLRRACNLIESIIRAKGHFLLANTDPEYNKIVQQMAKRTNQSYINHKWIGGFL

TNRKHMKNVQKHFQNFSAHSKFKDASTSSPFDFFPHFRKMQKCFEGIMTHDIPDCLII

INANKNSMAILEANQLQIPIVSLVDSNISNRLQKLITYPIPVNDDSIQFVYLFCNLIT

KTVILSQSAWPLSRKPLSRGRRP"

gene 13684..14211

/gene="ccmB"

CDS 13684..14211

/gene="ccmB"

/codon_start=1

/product="cytochrome c biogenesis B"

/translation="MIGFSKDFLCHFHSGLIWICLLFSFLPERFLQNDFEDGTLELYC

SSGYCLQKILLSKLVGHWVLQISGIFGTFPVVQLLYQFDQLKMNWFTLIIGSLIFTLM

CGIHSCLALGIISHSWNSLQNLTTLPTLLPLIIFCASTETEWFHVLLLMGYLLLFLFL

YPILVSITLQKLISQ"

gene 14715..15473

/gene="ccmC"

CDS 14715..15473

/gene="ccmC"

/codon_start=1

/product="cytochrome c biogenesis C"

/translation="MYFEILRPYLIMLCPFRYARILIGFCWFLAAMAIYLSIWVAPSD

FQQGENYRIIYVHVPAAWMSLLIYIAMAISSVLFLLTKHPLFQLFSKSGAKIGALFTL

FTLLTGGFWGKPMWGTFWVWDARLTSVLILFFIYLGVLRFQQFSADVASISICIGLIN

IPIIKFSVNWWNTLHQPSSISQFGISIHISMLIPILLILTSFFCLSGIFFILETRQII

LSFSSFSVKSQINSQNNNRKQVFFYTNNRSSKST"

gene 15600..17456

/gene="ccmFN"

CDS 15600..17456

/gene="ccmFN"

/codon_start=1

/product="cytochrome c biogenesis factor N"

/translation="MYNELGHYFLVPSIFVALTYNKRPAAISLYFFLFTISFFGILSC

YISSDFLNYNVFTNSNANAPLFYKISGTWSNHEGSLLLWCWILSFYGFFLGHRVRPCN

VSKRGRSKNIFFFRRPLVAFRSASFIRKENKQFRHHLLQNLFGTINHKSSLESQSEVT

PSGIVQEPSNKRLKDGTNAEENKRPIRLKEWKELKKKKSRFFFLLYALCNNLDSLFLA

QNANNKVSFIDERRIYMGIALFFSIFLLASSNPFVRISFVCTKSLAELNPVLQDPILA

IHPPCIYAGYVASAIGFCLCLAKIMNGISALYLPMRRESKAEIFDAFSRITNKLITQE

NAKKILKNIFENTCSLHPSFAFILRSNRSLLGLRHLVSPSPLRSKERLNLTKSQWTKR

VVRKANTAFLHFGWTRSANKGVSGPQCHGWKQIQIWILTCWCFLTVGILLGSWWAYHE

LGWGGWWFWDPVENASFMPWLLATACIHSVILPKLNYWTLFLNMVTFLCRVLGTFFVR

SGLLTSVHSFATDSTRGIFLWFFFLLITSISLMFFFQMKQQSSTRLVGALSNLPSNQS

LKAPKPVNQILWYSRRSTLFVHLRKFTRLSKLMEDEEGHDKLIVYKASKIHK"

gene 17590..19806

/gene="ccmFC"

CDS join(17590..18455,19218..19806)

/gene="ccmFC"

/codon_start=1

/product="cytochrome c biogenesis factor C"

/translation="MVQLQNFFFFLMFMVVLCGTAAPILFQWLVSRDVPTGAPFSHGT

IIPIFTSLLLLLVHVHSRGFIRSMEKTESFVLVRAKPIFLLNIIEKSSPKTRAKNAFF

FFSLFISNFSIFKFMGDLSYLESFCGVLCFLLFCTFFLSFKYRRDTWANKERRLEIEE

KRKPRKRAQRRKRQALCWPNKRKKQRNKKKEHFYFFSLSNKSKIFLIYLLQFPKTFGF

NEKAKILAFYSLLAFLQAYSFVLENIWNRFFLVRALPKRLMDVGHDFRKVPMTMKISH

GGVCIFIMGVILSNTKKRQFTQLLPLGSELHIGREHCCLRGIDQLHGPTFHSICGNLI

IYKPSLKNSFIFDYDESLRAIIDLLPIAALSYQNEKVEKKSIDFFSTFFHGDRSWRNR

EHHSFPLWLTVFPEKRFSFSNQETSTTKVAIHSNLFTDLYALIGTGSFETGWYITIMK

LPFIFCIWIGFILASLGGLRSFLRQLALYRLDWN"

exon 17590..18455

/gene="ccmFC"

/number=1

intron 18456..19217

/gene="ccmFC"

/number=1

exon 19218..19806

/gene="ccmFC"

/number=2

gene complement(19858..19928)

/gene="trnQ (UUG)"

tRNA complement(19858..19928)

/gene="trnQ (UUG)"

/product="tRNA-Gln"

gene complement(21201..21285)

/gene="trnL (UAG)"

tRNA complement(21201..21285)

/gene="trnL (UAG)"

/product="tRNA-Leu"

gene complement(22211..22294)

/gene="trnS (UGA)"

tRNA complement(22211..22294)

/gene="trnS (UGA)"

/product="tRNA-Ser"

gene 22407..22477

/gene="trnG (UCC)"

tRNA 22407..22477

/gene="trnG (UCC)"

/product="tRNA-Gly"

gene 22506..23093

/gene="rps4"

CDS 22506..23093

/gene="rps4"

/codon_start=1

/product="ribosomal protein S4"

/translation="MPASRFKTCRQISENVWQTKKLTQKQKAIILKLRKKANKKQSDF

FKELQTIQKLSLFYGKLPIKKMQRSKTQTYLDKKNSLLFDIERRLDVILIRLNFCLTI

FQARQLISHKKICVNYKMVNIPGFQLSKGDLISIQDNFLYFIRSKIRQNFQSNRIWRI

KPTHLEVNYRTLKAVVLYEPQQIQFPYSIDLDLLD"

gene complement(23811..24545)

/gene="tatC"

CDS complement(23811..24545)

/gene="tatC"

/exception="RNA editing"

/codon_start=1

/product="Sec-independent protein translocase protein"

/translation="-

NFLFKTILKEVRIRFFWIFICFSLTWFTCYWFSEDLFFLSAKSFLILSYSGFICTQLT

EALSTYVTISLISCFYFLFPFLSYQIWCFLIPSCYEEQRKKYNKFFYLSGFCFFLFFF

VTFVGIVPNVWHFLYELNKTSTNLLIIKLQPKIFDYILLTVRILFISSICSQVQVLVI

FLLESKGIFVKSCIKNRRFFMVLSIFTAAFLTPPDIWCQIVAYLLIYCIIELTIFYAL

IIQVYKKQLVL"

misc_feature 24544

/gene="tatC"

gene 24966..25039

/gene="trnR (UCU)"

tRNA 24966..25039

/gene="trnR (UCU)"

/product="tRNA-Arg"

gene 25191..25273

/gene="trnY (GUA)"

tRNA 25191..25273

/gene="trnY (GUA)"

/product="tRNA-Tyr"

CDS complement(join(25320..25339,27230..27512))

/gene="nad4L"

/codon_start=1

/product="NADH dehydrogenase subunit 4L"

/translation="MDLVKYLTFSMILFLLGIWGIFLNRKNILIMLMSIELMLLAVDL

NFLVFSVYLDDMMGQLFALFVLTVAAAESAIGLAILVITFRIRGTIAVEFINCMKG"

exon complement(25320..25339)

/gene="nad4L"

/number=2

intron complement(25340..27229)

exon complement(27230..27512)

/gene="nad4L"

/number=1

gene complement(27817..28077)

/gene="sdh4"

CDS complement(27817..28077)

/gene="sdh4"

/codon_start=1

/product="succinate dehydrogenase subunit 4"

/translation="MKAHRETLGHWLLQRMTAASLIPTILISNVSTLILLNILLFWHI

HVGIEEILTDYVHHEITRNWILILFRVFCLIIIKYVFLFFVF"

gene complement(28348..29643)

/gene="sdh3"

CDS complement(join(28348..28643,29544..29643))

/gene="sdh3"

/codon_start=1

/product="succinate dehydrogenase subunit 3"

/translation="MKINRPLSPHLTIYKPQLTSTLSILHRISGAFLAIMVFFSILSL

KIGDLNLTSYYLYRYAFFFTSYSYWLILSVVNFSLLALCYHMSNGIRHLLWDLGFFLE

LSKVYTSGIIMLFCAAFLAVSNIIRFYFS"

exon complement(28348..28643)

/gene="sdh3"

/number=2

intron complement(28644..29543)

/gene="sdh3"

/number=1

exon complement(29544..29643)

/gene="sdh3"

/number=1

gene complement(29669..29742)

/gene="trnP (UGG)"

tRNA complement(29669..29742)

/gene="trnP (UGG)"

/product="tRNA-Pro"

gene 30578..32304

/gene="rrn18"

rRNA 30578..32304

/gene="rrn18"

/product="18S ribosomal RNA"

gene 32821..32893

/gene="trnM (CAU)"

tRNA 32821..32893

/gene="trnM (CAU)"

/product="tRNA-Met"

gene 33815..33936

/gene="rrn5"

rRNA 33815..33936

/gene="rrn5"

/product="5S ribosomal RNA"

gene 34187..34261

/gene="trnMf (CAU)"

tRNA 34187..34261

/gene="trnMf (CAU)"

/product="tRNA-Met"

gene 34786..38168

/gene="rrn26"

rRNA 34786..38168

/gene="rrn26"

/product="26S ribosomal RNA"

gene 40333..40866

/gene="rpl10"

CDS 40333..40866

/gene="rpl10"

/codon_start=1

/product="ribosomal protein L10"

/translation="MLIKQIVIRKKAQEIEKKSPYILLFHCSGLTSRQWRQLKNLLCA

FRGRTLFQPNYKHKCPQKNKQDGKQGVFIEQLAYSAGPTCILYLTKEAPDNTWSQLLQ

PKYNQNLVLLYGQLQSTLVNHMDIKKAANLEITPVFQQLFELIFYPYNSLCFCLNKPI

HASPTRRKEGEDARKVT"

gene complement(41067..41139)

/gene="trnH (GUG)"

tRNA complement(41067..41139)

/gene="trnH (GUG)"

/product="tRNA-His"

gene complement(41166..41245)

/gene="trnL (CAA)"

tRNA complement(41166..41245)

/gene="trnL (CAA)"

/product="tRNA-Leu"

gene complement(41247..41326)

/gene="trnL (UAA)"

tRNA complement(41247..41326)

/gene="trnL (UAA)"

/product="tRNA-Leu"

gene 42433..48772

/gene="nad5"

CDS join(42433..42663,43513..44034,44863..45564,48197..48772)

/gene="nad5"

/codon_start=1

/product="NADH dehydrogenase subunit 5"

/translation="MYLLIVTLPLLGSCVAGAFGRFLGSRGTAIVTTTCVSLSFILSL

IVFYEVALGASACYIKIAPWIFSEMFDASWGFFFDSPTVVMLIVVTFVSSLVHLYSIS

YMSEDPHSPRFMCYLSIFTFFMLMLVTGDNFIQLFLGWEGVGLASYLLINFWFTRLQA

NKAAIKAMLVNRVGDFGLALGIMGCFTIFQTVDFSTIFARAGAFSELHHYFIFCNMRF

HAITVICILLFIGAVGKSAQIGLHTRLPDAMEGPTPVSALIHAATMVTAGVFMIARCS

PLFEYSPTALIVITFVGAMTSFFAATTGILQNDLKRVIAYSTCSQLGYMIFACGISNY

SVSVFHLMNHAFFKALLFLSAGSVIHAMSDEQDMRKMGGLASLLPFTYAMMLIGSLSL

IGFPFCTGFYSKDVILELAYTKYTISGNFAFWLGSVSVFFTSYYSFRLLFLTFLASTN

SFKRDILRCHDAPILMAIPLIFLAFGSIFVGYVAKDMMIGLGTNFWANSLFILPKNEI

LAESEFATPTIIKLIPILFSTLGAFMAYNINFVANPFIFALKTSPLGNRLYCFLNKRW

FFDKLFNDFLVRFFLRFGYEVSFKVLDKGAIEILGPYGISYTFRKLAKQISKLQSGFV

YHYAFVMLIGLTIFITIIGLWDFISFWVDNRLYFIYIVSFLFIHFENDISTN"

exon 42433..42663

/gene="nad5"

/number=1

intron 42664..43512

/gene="nad5"

/number=1

exon 43513..44034

/gene="nad5"

/number=2

intron 44035..44862

/gene="nad5"

/number=2

exon 44863..45564

/gene="nad5"

/number=3

intron 45565..48196

/gene="nad5"

/number=3

exon 48197..48772

/gene="nad5"

/number=4

gene 49393..51661

/gene="nad4"

CDS join(49393..49853,50635..51661)

/gene="nad4"

/codon_start=1

/product="NADH dehydrogenase subunit 4"

/translation="MLQFLAPFYSNLSGLILCPLLGSIILFVIPDPRIRLIRSIGLCT

SLITFLYSLLFRIQFDNSTAKFQFVETIRWLPYSNINFYIGIDGISLFFVVLTTFLIP

IRILVGWSSIKSYKKEYMIAFLICESFMIAVFSMLDLLLFYVFFESVLIPMFIIIGVW

GSRQRKIQAAYQFFLYTLLGSVFMLLAILFIFFQTGTTDLQILLTTEFSERRQILLWI

AFFAPFPVKVPMVPVHIWLPEAHVEAPTAGSVILAGILLKLGTYGFLRFSIPMFPEAT

LYFTPFIHTLSVIAIIYTSLTTIRQIDLKKIIAYSSVAHMNFVTIGMFSLNIQGIEGS

ILLMLSHGMVSSALFLCVGVLYDRHKTRLVKYYGGLVSTMPMFSTIFLFFTLANMSLP

GTSSFIGEFLILVGAFQRNSLVATLAALGMILGAAYSLWLYNRVIFGNFKPKFLQKFS

DLNRREVLIFFPFIVGVIWMGVYPEVFLECMHTSVSNLVQHGKFD"

exon 49393..49853

/gene="nad4"

/number=1

intron 49854..50634

/gene="nad4"

/number=1

exon 50635..51661

/gene="nad4"

/number=2

gene 51688..54128

/gene="nad2"

CDS join(51688..51843,52815..54128)

/gene="nad2"

/codon_start=1

/product="NADH dehydrogenase subunit 2"

/translation="MFEHDFLALFPEIFLINATIILLIYGVVFSTSRKYDYPPLVCNV

SWLGLLSVLITILLVASSTPLTVANLFYNNLIIDNFTYFCQIFLLISTASTIVMCLGY

FKEESLNAFESIVLILLSTCSMLFMISAYDLIAMYLAIELQSLCFYVIAASKRDSEFS

TEAGLKYFILGAFSSGILLFGCSMIYGFTGVTNFEELAKIFTGYEITLFGAQSSGIFM

GILFIAVGFLFKITAVPFHMWAPDVYEGSPTLVTAFFSIAPKISILANMVRVFIYSFY

DPTWQQLFFFCSIASMILGALAAMAQNKVKRLLAYSSIGHVGYLFIGFSCGTIEGIQS

LLIGVFIYVLMTINVFAIVLALRQNRFKYIADLGALAKTNPILAITLSITMFSYAGIP

PLAGFCSKFYLFFAALGCGAYLLALIGVVTSVISCFYYIRFVKIMYFDTPKKWILYKP

MDREKSLLLAITLFLISFFFLYPSPLFLVSHQMALSLCL"

exon 51688..51843

/gene="nad2"

/number=1

intron 51844..52814

/gene="nad2"

/number=1

exon 52815..54128

/gene="nad2"

/number=2

gene complement(54625..54696)

/gene="trnG (GCC)"

tRNA complement(54625..54696)

/gene="trnG (GCC)"

/product="tRNA-Gly"

gene complement(54788..54861)

/gene="trnR (ACG)"

tRNA complement(54788..54861)

/gene="trnR (ACG)"

/product="tRNA-Arg"

gene 55493..55565

/gene="trnE (UUC)"

tRNA 55493..55565

/gene="trnE (UUC)"

/product="tRNA-Glu"

gene 55888..56268

/gene="rps12"

CDS 55888..56268

/gene="rps12"

/codon_start=1

/product="ribosomal protein S12"

/translation="MPTINQLIRHGRKSKRRTQRTRALTQCPQKQGVCLRVSTRTPKK

PNSALRKIAKVRLTNRNEIIAYIPGEGHNLQEHSVVMVRGGRAKDLPGVKYHCIRGIK

DLQGIPSRRRGRSKYGTKKPKDSI"

gene 56265..56984

/gene="rps7"

CDS 56265..56984

/gene="rps7"

/codon_start=1

/product="ribosomal protein S7"

/translation="MNLFVKSSNFSFVFGLFFDWFHQSKLSEKAGMEKKENWPFSGKN

LFFFSESFFARRLSHCRYLCYALPGHVPSRPKGREASIYNSSDNLGYIRGLHGKQKQL

IKKLVHICMIDGKKTRSRAIVYKTFHCLAQHGDILRLLVNAIENVKPVCEVKKVRISG

TTQLVPSIIATNRQETLAIRWMLEAAAKRRINKKSMSLDQCLADEILDASRKMGIARK

KRDDLHKLAQANRSFSHYRWW"

gene complement(57716..59088)

/gene="atp6"

CDS complement(join(57716..58394,59009..59088))

/gene="atp6"

/codon_start=1

/product="ATP synthase F0 subunit 6"

/translation="MACSPLEQFAIIPLIPIHIGNFYLSFTNSSLFMLLTISLVLLLV

HFVTLNGGHLVPNAWQSFVEIIYDFVLNLVNEQISGPSSIKQRFFPLIFVTFTFLSFS

NLIGMIPYSFTVTSHFIITLGLSLSLFIGITIVGFQTHGLHFFSILLPQGVPLPLAPF

LVLLELISYRFRALSLGIRLFANMMAGHSLVKILSGFAWTMLSMGGIMYLAHLAPFLI

VFALTGLELGVAILQAYVFTILICIYLNDAINLH"

exon complement(57716..58394)

/gene="atp6"

/number=2

intron complement(58395..59008)

/gene="atp6"

/number=1

exon complement(59009..59088)

/gene="atp6"

/number=1

gene 60338..60943

/gene="nad6"

CDS 60338..60943

/gene="nad6"

/codon_start=1

/product="NADH dehydrogenase subunit 6"

/translation="MILFSVFSSIALVSSVMVIRAKNPVHSVLFFILVFFNTSGLLVL

LGLDFFAMIFLVVYVGAIAVLFSFVAMMLNIKIAEIHENVLRYLPVGGIIGVIFLLEI

FFIVDNDYIPILPTKLSTTYLTYTVYAEKIQSWTNLETLGNLLYTTYFVLFLVSSLIL

LVAMIGAIVLTMHKTTQVKRQDVFRQNAIDFKNTIKKIRDI"

gene 61957..66442

/gene="cox2"

CDS join(61957..62066,63080..63348,64742..65059,66378..66442)

/gene="cox2"

/codon_start=1

/product="cytochrome c oxidase subunit 2"

/translation="MSLKNTWLFPIGYCDAAEPWQLGFQDAATPMMQGIIDLHHDIFF

FLIIILIFVLWMLVRALWHFHYKRNPIPERIVHGTTIEIIWTIFPSIILMFIAIPSFA

LLYSMDEVVDPTITIKAIGHQRYWTYEYSDYNGSDEQSLTFDSYMIPEDDLELGQSRL

LEVDNRVVVPAKTHLRMIITSADVLHSWAIPSLGVKCDAVPGRLNQTSIFIKREGVYY

GQCSELCGTNHAFMPIVVEAVSLDDYVSWISNKLD"

exon 61957..62066

/gene="cox2"

/number=1

intron 62067..63079

/gene="cox2"

/number=1

exon 63080..63348

/gene="cox2"

/number=2

intron 63349..64741

/gene="cox2"

/number=2

exon 64742..65059

/gene="cox2"

/number=3

intron 65060..66377

/gene="cox2"

exon 66378..66442

/gene="cox2"

/number=4

gene 66462..68090

/gene="cox3"

CDS join(66462..66967,67799..68090)

/gene="cox3"

/codon_start=1

/product="cytochrome c oxidase subunit 3"

/translation="MSVSQKHPYHLVDPSPWPLLGSLGALASTIGGVMYMHSFMGGGT

LLSLGLGMILYTMFVWWRDVIRESTYEGHHTVVVQLGLRYGMILFIVSEVMSFLAFFR

AFFHSSLAPTVEIGAIRPPKGIDVLNPWGIPFLNTLILLSSGAAVTWAHHAILAGFKK

QAVYALVATISLALVFTGFQGMEYVEAPFTISDGIYGSTFFLATGFHGFHVIIGTIFL

IICAIRQYLGHFTQTHHFGFEAAAWYRHFVDVVWLFLSVSIYWWGGN"

exon 66462..66967

/gene="cox3"

/number=1

intron 66968..67798

/gene="cox3"

/number=1

exon 67799..68090

/gene="cox3"

/number=2

gene 69468..72071

/gene="nad1"

CDS join(69468..69754,70517..70957,71813..72071)

/gene="nad1"

/codon_start=1

/product="NADH dehydrogenase subunit 1"

/translation="MRLYIIGILAKILGIIIPLLLGVAFLVSAERKVMASMQRRKGPN

VVGLFGLLQPLADGLKLMIKEPILPSSANLFIFIMAPVITFMLSLVAWAVIPFDYGMV

LSDLNVGILYLFAISSLGVYGIITAGWSSNSKYAFLGALRSAAQMVSYEVSIGLIIIT

VLICVGSCNFSEIVIAQKQIWFGIPLFPVFIMFFISCLAETNRAPFDLPEAEAELVAG

YNVEYSSMGFAPFSPGEYANMILMSSLCTLLFLGGWLPILDIPIFYVIPGSIWFSIKV

LFFLFVYIWVRAAFPRYRYDQLMRLGWKVFLPLSLAWVVFVSGVLVAFDWLP"

exon 69468..69754

/gene="nad1"

/number=1

intron 69755..70516

/gene="nad1"

exon 70517..70957

/gene="nad1"

/number=2

intron 70958..71812

/gene="nad1"

/number=2

exon 71813..72071

/gene="nad1"

/number=3

gene 72429..74592

/gene="cob"

CDS join(72429..72848,73792..74592)

/gene="cob"

/codon_start=1

/product="cytochrome b"

/translation="MARRLSILKQPIFSTFNNHLIDYPTPSNISYWWSFGSLAGLCLF

IQIITGVFLAMHYTPHVDLAFLSVEHIMRDVKGGWLLRYMHANGASMFFIVVYLHIFR

GLYYGSYSSPRELVWCLGVVILLLMIITAFIGYVLPWGQMSFWGATVITSLASAIPVV

GDTIVTWLWGGFSVDNATLNRFFSLHYLLPFIIAAAAIIHLAALHQYGSNNPLGINSS

VDKIAFYPYIYVKDLVCWVAFAIFFSIFIFYAPNVLGHPDNYIPANPMSTPAHIVPEW

YFLPVYAILRSIPNKLGGVAAIGLVSVSLFALPFINTSYVRSSSFRPIHQKLFWLLLA

DCLVLGWIGCQPVEAPYVTIGQIASVGFFFYFAITPILGKLEARLIQNSNVCEDLSPR

KLSHIFKNSIYVVK"

exon 72429..72848

/gene="cob"

/number=1

intron 72849..73791

/gene="cob"

/number=1

exon 73792..74592

/gene="cob"

/number=2

gene 75610..78169

/gene="nad9"

CDS join(75610..75841,77814..78169)

/gene="nad9"

/codon_start=1

/transl_except=(pos:75610..75612,aa:Met)

/product="NADH dehydrogenase subunit 9"

/translation="MDNQSFFKSLIATLPKWIHQFQKSKHENILYTNPDYLFQLLWFL

KYHTNTRFQVLIDIGGVDYPSRKQRFEVVYNLLSIQYNSRIRVQTSVDEITPICSAVN

IFPSAGWWEREVWDMFGVYFSDHPDLRRILTDYGFEGHPLRKDFPLSGYVEVRYDDSE

KRVVSEPIEMTQEFRYFDFASPWEQSSRSDKSRKK"

exon 75610..75841

/gene="nad9"

/number=1

intron 75842..77813

/gene="nad9"

/number=1

exon 77814..78169

/gene="nad9"

/number=2

gene 78632..81338

/gene="atp1"

CDS join(78632..79775,80926..81338)

/gene="atp1"

/codon_start=1

/product="ATP synthase F1 subunit alpha"

/translation="MNKLTGNKLAGAELSTLLEQRITNYYTKLQVDEIGRVVSVGDGI

ARVYGLNKIQAGEMVEFASSVKGMALNLENENVGIVIFGSDTAIKEGDIVKRTGSIVD

VPVGKALLGRVVDALGVPIDGKGALSAAERKRVEVKAPGIIARKSVHEPMQTGLKAVD

SLVPIGRGQRELIIGDRQTGKTAIAIDTILNQKQINTQGTSDSEKLYCVYVAIGQKRS

TVAQLVKILSEAGALEYCIIVAATASDPAPLQFLAPYSGRAMGEYFRDNGMHALIIYD

DLSKQSVAYRQMSLLLRRPPGREAFPGDVFYLHSRSSERAAKMSDQTGAGSLTALPVI

ETQAGDVSAYIPTNVISITDGQIFLETELFYRGIRPAINVGLSVSRVSSAAQLKAMKQ

VCGSSKPELAQYREVAAFAQFGSDLDAATQYLLNRGARLTEVLKQPQYSPIPIEKQIV

VIYAAVKGYLDQIPISSINKYEHELLKSIDSDILSAIVQQKNITEQINSQLATFCKKF

TQSFLATHSV"

exon 78632..79775

/gene="atp1"

/number=1

intron 79776..80925

/gene="atp1"

/number=1

exon 80926..81338

/gene="atp1"

/number=2

gene complement(81784..81856)

/gene="trnW (CCA)"

tRNA complement(81784..81856)

/gene="trnW (CCA)"

/product="tRNA-Trp"

gene 81880..82236

/gene="nad3"

CDS 81880..82236

/gene="nad3"

/codon_start=1

/product="NADH dehydrogenase subunit 3"

/translation="MEFAPICVYLVISLLFSLILIGVSFLFASSSNSAYPEKLSAYEC

GFDPFDDARSRFDIRFYLVSILFIIFDLEVTFLFPWAVSLNKIGLFGFWSMIVFLLIL

TIGFLYEWKKGALDWE"

gene 83201..83273

/gene="trnV (UAC)"

tRNA 83201..83273

/gene="trnV (UAC)"

/product="tRNA-Val"

gene 83345..83418

/gene="trnD (GUC)"

tRNA 83345..83418

/gene="trnD (GUC)"

/product="tRNA-Asp"

gene 83421..83493

/gene="trnA (UGC)"

tRNA 83421..83493

/gene="trnA (UGC)"

/product="tRNA-Ala"

gene 83782..83854

/gene="trnT (GGU)"

tRNA 83782..83854

/gene="trnT (GGU)"

/product="tRNA-Thr"

gene 86684..88075

/gene="rpL2"

CDS 86684..88075

/gene="rpL2"

/codon_start=1

/product="ribosomal protein L2"

/translation="MTLKQLTFRLKKKSAGRNSSGRITVFHRGGGSKRLHRKIDFQRS

TSSIGLVQRIDYDPNRSSWIALVRWLRAMKQAEAANSQTEENAKRFLGRREKNLFFFG

LLFSFSSLSRKAQRRNYVFFSALFSLETKREAAILGSFGSFLDLPRIALAGAKPAFFA

SRMKDFRGHNTFYKNESGRWKTHSEVQRIERKALSWRTNLFFSFKPKHSEEEPMVEAG

KVDRAPFTYILASDQLEAGKTVMNCDWSKPSTSFDQYKSSHNLLAHNDLRFQNHFVHT

TNEGQRSLRVEEPVQRSQAASWLRPGEDYASNENKNILDSYYQMVGNCVALANIPIGT

WIHNIEWNPGQGAKLIRAAGTFAQIIKKFENTPQCIVRLPSGVDKLIDSRCRATVGIV

SNLHHGKRKLDKAGQSRWLGRRPIVRGVAMNPVDHPHGGGEGRTKGGRPSVSPWGKPA

KGGFRTVVRKRRN"

gene 88079..88360

/gene="rps19"

CDS 88079..88360

/gene="rps19"

/codon_start=1

/product="ribosomal protein S19"

/translation="MTRSVWKGPFVDACLFKQKKIRWKIWSRRSCILPQFVGCYAQIY

NGKGSVGLKITEEMVGHKFGEFASTRKPSSSGKRASPLKTKIKQKKKVR"

gene 88367..89983

/gene="rps3"

CDS 88367..89983

/gene="rps3"

/codon_start=1

/product="ribosomal protein S3"

/translation="MAQKINPISVRLNLNRSSDSSWFSDYYYGKLLYQDVNFRDYFNL

IRPPTGKTFGFRLGKFIIHHFPKRTFIHVFFLDRLGRSRHTGLGAIQSVKLIRHIDDA

TKIQRNEVKIRRYGYDDRLPSMHEIDQLLRISGWMASKNSTSLRNDALLENDDRKMSE

KSYAFSCFGSLRQISDVFPQTIFAAVRAPLNHLVMQYLFYSKNRIQFDPIVNIISNLA

ARSIIKKYITKEAKKKEDSLKKRMRSILLNKSICSKKEGLTYMDKTAQGSYVEALRGS

THFIRQANEVGFARKNRPEISPNIQTAYSVWLFSKDINSGRTEMRSAEELLALRTALP

SFVRALTFPHQNALHCLRKQSLLRLRFQIHREQGMPLANNYVIKNTEPIRQPGSILDF

GAPFISRDAEWKKVHSLFSRYYYWKKMQFFLSNQTKTNTLIRPVKIASVYQSASLIAQ

EISWKLEQKKSFRQICRSTFQEIEKCQYVKGIRICCSGRLNGAEIAKTECRKYGETSL

HVFSNQIDYAKAQASTPYGILGVKVWVSYF"

gene 90004..90411

/gene="rpL16"

CDS 90004..90411

/gene="rpL16"

/codon_start=1

/transl_except=(pos:90004..90006,aa:Met)

/product="ribosomal protein L16"

/translation="MLYPKRTKFRKYQKGRFKGCKADGTQLCFGRYGMKSCEAGRISY

QAIEAARRAISREFRRNGQIWVRVFADIPITSKPTEVRMGKGKGNSTGWIARVVEGQI

LFEMDGVSLSNAQQAATLAAHKLCLSTKFVQWF"

gene 91149..91709

/gene="rpL5"

CDS 91149..91709

/gene="rpL5"

/codon_start=1

/product="ribosomal protein L5"

/translation="MFTPNRLRFHYENLLRQDLLLKLNYGNIMEVPRLCKIIIVPKAP

SNLIKNVKLAMEIVCGQKFIRTRSRDSAGKSFRFNKFILNQESKKDTGYITYLAQSTL

RGHTMYNFLEKLITIISFYDYPVKVQKSSIQLAMPTPLLRLFPEIQNHFEIFEHIQGF

DVTIVTSAKTQDEAFILWSGFLQKEV"

gene 91714..92013

/gene="rps14"

CDS 91714..92013

/gene="rps14"

/codon_start=1

/product="ribosomal protein S14"

/translation="MSNQIIRDHTRRLLVAKYELERMQCKAISRHKNLPNQIRYEYFL

KSSKLPRNSSKTRVRNRCIFTGRPRSVYKLFRVSRIVSRELASKGSLIGINKSCW"

gene 93586..93891

/gene="rpL6"

CDS 93586..93891

/gene="rpL6"

/codon_start=1

/product="ribosomal protein L6"

/translation="MEAKFFCFLEIIGVGYKASTNPQGSILYPKLGFSHEIRLQVTSA

VRVLCFKPNIICCTGIDHQKVTQFAASIKSCKPPEVYKGKGIQYRNEILRKKQGKKK"

gene 93895..94263

/gene="rps13"

CDS 93895..94263

/gene="rps13"

/codon_start=1

/product="ribosomal protein S13"

/translation="MSYILGTNLMPNEQVEIALTRIFGLGPKKAIQVCAQLGFNDNIK

VNKLTKYQIDRIIKIISQNYLVDLELARVIQRDIKQLMSIGCYRGFRHNAGLPLRGQR

THTNAKTCRKFRNVSINQRS"

gene 94718..95074

/gene="rps11"

CDS 94718..95074

/gene="rps11"

/codon_start=1

/product="ribosomal protein S11"

/translation="MQKKKKHGIAYIRSTLSNTIITVTDHKGDTKTWSSSGSLGFKGS

RRSTNYAAQATAENAARTAIQLGIKSVEVEIKGLGYGKESSLRGLRLGGLIITEIRDV

TPTPHNGCRPPKKRRV"

CDS join(95722..95742,97151..97216,98396..98403,99659..99788)

/gene="atp9"

/codon_start=1

/product="ATP synthase F0 subunit 9"

/translation="MLEGAKLIGAGAATIASAGAAIGIGNVFSSSIHSVARNPSLAKQ

LFGYAILGSASTEAIALFASMMAFLILFVFQ"

exon 95722..95742

/gene="atp9"

/number=1

intron 95743..97150

/gene="atp9"

/number=1

exon 97151..97216

/gene="atp9"

/number=2

intron 97217..98395

/gene="atp9"

/number=2

exon 98396..98403

/gene="atp9"

/number=3

intron 98404..99658

exon 99659..99788

/gene="atp9"

/number=4

misc_feature 99786

ORIGIN

1 cgacaggtta cgttggattc gatatgctta ctagcatatc gaatccaggg cttatagttt

61 aattggttca aacgcaccgc tcataacggt gatattgtag gttcgagtcc tactaagcct

121 atattctata aaaccaaaat aataaaatta gactgaaatg gatgcgtaac acgctgcgcc

181 ttgaatcccc caaatatata tatatttttt tgctccgcag gtcattgttg ttttttatca

241 atttatcatc attaaattac ataatgataa aaaaaaaata tatatatatt tttttgggtg

301 tatagcttaa ttggtagagc attaggcttt taacttaatg gtcgcaggtt caagtcctgc

361 tatacccaaa tgtttttata gaagttcccc tggcagttca tatgcacatc aaaatataca

421 ttgtacgttg gctcgtctag tacgagtaat tacgttaaac taaaatatat atatattttt

481 ttatgtcttt atggccgctc acgccctttt ttttccccgc gagaaacctt tggcctctaa

541 tctaagctca gatagtgaaa aaaaatgcaa agagagaagt tacaagaaga tttcgagctt

601 agattgggcg aagagaactg gcagcccaaa tagtaggatg cggctctgaa aagagagaag

661 tgttgtaggt gttaccaact actatttcta ttctattttt ctatatgtta acaatctgca

721 gcaatcctgc agtgttggag ttgtttgata gcaactgtga ataacagtat agatatagat

781 atcttcatat ttagctcccg caaattcatg tcatatctat gatttttcca tgaagagtgg

841 attcccgtta ttttcaagtt ctgccagtaa tctacgtcac cgtcaacatg cattctcttt

901 ctgtccgttt cattcattct ctacatgatg ccaacctatc ttattctatt gcccggcaat

961 agaatcaata ctggctcttc atttatggag aagcagtgct cttcatttct agtctttttt

1021 ctctctactc tttcttgttc tcttggccat ttctcaacca ttttctgtag tttgaagctc

1081 ggcctttctg tagtttgaag cgagtagtca tttctgctct attcagaaaa cgttttgctt

1141 tgccttgtcc aattcatctt tttttgccat tccttttctc catgagtaga gaggcgaggt

1201 ggggctccgc ccccccaatg ggcagagggc agagcgggta agtgcttaag tggcaccatc

1261 tgctaagacg tctaaatgct ttccttgata accggaagtt ctgaaaaagt gtgaaatgcc

1321 ggagggcttt tgaccatcca ttcaagtgtc gttgaattct gttcaacagc ccaaggactt

1381 gaagcacact tgttttcact ggtgagagtc aaaaaaacca ctacaaagaa acacaaaatt

1441 cctactacag aaacatacga gccgaaacta ctaaaggcat tccatccagc gtaagcatct

1501 ggataatctg gaatgcgacg tggcatacct gcaagaccta aaaaatgcat aggaaaaaaa

1561 gtcaagttca caccaaagaa agtgatccaa aaatgaatct gacctaaagt ctctggatat

1621 tgaagaccag ttattttacc tatccaataa tagaatccgg caaataaagc aaaaacagct

1681 cccatagaaa gaacataatg gaaatgtgca accacataat aagtatcatg tagagcgatg

1741 tccagcccag aattggccaa tactattcca gtaagagtag agtaggtgcc cttactcgcg

1801 tggggccaat tttgagtttc ccttgtgctt ttagtgcacc tctctcgtaa ccgtacatga

1861 tagttttccc atcatacggc ttgcacgcgc gtttaattct tttacgactt ggcacgggtt

1921 tcatagcctg taccgacgtg tcgaacaagg ctgcggtgcc tctccggttt tcctcttttt

1981 cccccgagtt ccttttttcg atcggggaga gagagcccga agaagtattt tccgtggtcg

2041 gctctctcgt gctcgagctt gcaaagggat atttgttttc tgttgaatat ggtcatcatc

2101 tcgtttatct gtgagatatt tttgtcgagt tggctcaact tgcgcatgtg gttaatcttt

2161 attggtccgg atttgttgac taggcatttg gtatcgtccg gtatagatcc gccgcatagc

2221 tttttggaaa gaagcgtagc gggaccaagc tttttggttg gggttttttt taatgctcgt

2281 gtgccctcct tgttttggtt cggacttcta ttactgcaga acaccagaaa ggaacctctg

2341 acttaattcc gggttccttt ccctgtaaaa ttattaagat atatcacttc tggagaagcc

2401 cttttcctct tgttctaatc cgtcccattg tagttgtaag gcgtttgctt tttttagatc

2461 gtgccttagt attacttgtt gttttttcga gaatttttcg tagacgagtt gaccctttct

2521 ggttgggact ttgtatttgg ctgcgtaccc tgcgctaaaa tttttgtacg ttttcgaggc

2581 ttttcgttgc cgcaatttta tgatcaaggt gcgaacacag cagctcctga tgacgagttt

2641 cagataattt gttaattggt agaacttatc aacgaacgaa tagtagttgc atattcttct

2701 taggatccag cctttgtagt cccctcgtat tttgctctta gcgccttaga aacgaatcgt

2761 ataagtctcc tatctttaaa caggtttacc caaattagat ggtcgattaa gccgtagtat

2821 tttgttatat gccctttcag agctcagtgg tgcttttgca gtccagttcc gattaaccgt

2881 gggccaaata aatgcaaggg tcgaaaacct ggataagggc accggtttgt aaataactta

2941 tatatactgt tgtcccaatt gcttctagaa tgatcttttc tctagagggc cactgtagta

3001 agcggcgggc gttcgcctcc ctcgaagtgc ctcataccct gttgaaattg ggagcgttct

3061 gagcgaagtt gtttattttt cttctgctga cgtttactat tttgccgtca agggccggtg

3121 tacttcgcct tcgccctggg atggaccaaa acatttgtat gccaactcga tcaggtctcc

3181 ttgaaataga aatctgcaag ggttaaggtt gttgttaagg ttgcctttgc cgttgttaag

3241 aatggaatgc agttctttcg ctatcaggtt agttatccgc ctgactagtg aagtcctcac

3301 acgcctgtgc gcctggcccg cgtagcaagg gtatagcaaa aagatttttc caaacctcat

3361 ttgttgaggg cctcctcagc aaagtgaagt ggtgggtcac ctgctgtccc gtgcttgaca

3421 gaaatggcgt catccggtta ggtgttggga ttgggaacct caagtccttc atagcgggcg

3481 aggcttactt gagccttgaa ttgcttgcac cctactactt actatttggc cacgcacgag

3541 gttttcgaga gagttgacgc gttgacccgg tagggggtcc accgggttcg cctcccgtta

3601 gtgcaaatta cctcgttgtt tttggttcct tccgttgcct ttttagggta ccacctttcg

3661 gaagccccca aaggaggggt tttttcaccc tacttatctt cgcttctggg gtctccccta

3721 cacgtcaact ggagagagaa tacgtccgtc tgtcgccatt tctggggtta tggcgaagta

3781 aacgtcatcc attccggtta gcacgtcgca cccccctaca gtaaacaaga agataaatcc

3841 tacagcaaat aacatgggtg ttttgtattg tattgaacct ccccacatgg tagcaatcca

3901 actaaaaatt tttattccag taggcacggc aataatcatg gtagccgcag tgaagtaagc

3961 acgtgtatca acatctaggc ctacagtaaa catgtggtgc gcccacacaa taaatccaag

4021 aactccaata ctgatcaagg cataaaccat gcctagataa ccaaatacgg gttttcttga

4081 aaaggtagaa acgatatgac taatgatacc gaatcctggc aaaattagaa tatagacctc

4141 aggatcggga tagattttgc cgttcccagc aaagcccccc acagaaccca gcgagctcct

4201 cttaaagcac tgggctcttc gcggaatatg cctataaact atgtagtcta tccccaagca

4261 tgttctgtaa caagacacca agagtgcaca agggatttgt gcaacaaatt accattacca

4321 ggcacttccg agttcttata aaaaggccgc aggtcatgaa tctctaaatt agagctagtc

4381 gaataaccta agccttgatt gcatacggga catatacctt tctggcggat gaatagcctg

4441 ctaaattcgt tacctttccc gtccacaaaa atttcccgat agctttgaat tcgaagattc

4501 cattcatcaa agggggttga atctacaaaa ggattaccta gatcacgaga tgctcgaaac

4561 atatgagcag gaacttcttt taccatagac gcaaggagtg ttatccatat cacgtcagca

4621 caatggcgtt tggcatctac actgggctcg gtccaagtag catgaaaatc ccagagtctg

4681 ccacggggag agggcacccc ctgatagaat cgggaaacca gtctgggtcg aggagtttta

4741 gagaatttac gatgtaaata tcgccagctt ctatgccaga tccaatgatc cagcagactg

4801 aaaatatgaa gaaagttgcc aattccaaag tagttgcccc aaccatgaag aattgggttt

4861 accaatttga tcatctggta aggagagaga tctatatttt cagaaaagac attttttatt

4921 ttccatttca gcgacattat cctattagga ttaggataca cgtaaaggcc cccacgagtg

4981 aagttttttc ctaccttaca tgaggaagtg acttgactat gagagcgagc cctatcgata

5041 ttgtggaata tgaagccgat aaaattaagt ctctctccga tcttccacgg gaatatgcgg

5101 gttttttctg acgacaattg aaggccacgt tccgccagga aactttttgc tttttcaaaa

5161 agtcgttcca ctaatatctc atcattggta ataataacaa agtcatcagc gtacctgata

5221 aggcggactg attctgtttt tcgagtccgg ttaaagagat aactatggcc tttcctctgc

5281 atccattctg tccttttagg atcagtcata gtggtccggt gtccgctagt tatctttttt

5341 tctaagccat ccagggcaaa gttcgcgatt aaaggactta tgacaccgcg gaacgagact

5401 tcaagttccc cttgatattc aattggactc ttaagccatt cctcgagtac aatttctgta

5461 ctactaggca tgggaaaatt atctaagatc catcggtgaa atattcggcc gaagaaacct

5521 tttatatcag catcaattac ccatttggaa acaaaatttt tactattttg ttctattttc

5581 ttgttgctaa ctttgcgtac tcttggcctt ctcgtgtcta gtatgtaagc aatttcacct

5641 accgccatgt gtgcacattt tccccttcga gatccaaagc tatatttgtc agcaaagggt

5701 tctgttacag gttcaatagc tagtttgaac aagtgttgga cggtcctgtc aaatattgtg

5761 ggtattctta gcagcctttc accatttttt ggttcgaaaa tggaaacatg gcgaacgggt

5821 gagctcttgt agttctttag attgatccaa aagatacgac gaactagacc gattcgggaa

5881 gaagcttcta gtattttacc atcgactccc ggaattcgac ttccagaagt ataatagaaa

5941 ttatctacgg caattattcg agaggttaat tgagtacaga tttcaagcat gcagtctttc

6001 aggagtgggt ttccgggccc cagggaagct gctaaaagag agaggatcct ttgttggttc

6061 tttactaatt gatggatagc cgtaaatttc ttttccaaca ttggccaccg accctcgttc

6121 atgatgacca cggctttcct ggcgataatt cgtgattttt ttcgggtctc cttccattca

6181 gcgaagagac tgtctggaga tcctgagcca ttagagaagc cacgtctctc tttccacgtc

6241 aaacgtttca gcattaccca catgttattg tgtatagcct tacgtttcat ctcaccttgt

6301 gatagcatca ttgacttgga tatatcaaca atgtccagtt gaatggaaat gccaatttcg

6361 gctcttgaaa aaggttccac cgcaagggca acgctaccaa tagaatatct gtcttttcga

6421 aagatgttgg gtctcgagtg gtccgcccgg gcaagggccg aaggcttctt gcacacaacg

6481 tgtgaaatct tacccttaaa agaaaggagg gcacactcca atcccaaaag atccctacta

6541 ttaccggggt ctgacctaaa agagtttgaa actaaaacaa aagggccttt tttccttttt

6601 ctggcaccat cctctacctt tcttatgaca tcgactccca aacatcccac ctcattgcca

6661 gttatctgca ttccaggcag atagtttatt tgaggcacag aacagctgtg ctcgcttagg

6721 tagcgctgta agggcagcgt accacctttt ctattggcgc aatcgcgccc atgaccaaaa

6781 aaccaaaaga gatgctggta taatattgga tctcctcctc ctgcaggatc aaaaaaggtt

6841 gtattaaagt tcctatcagt taataacatg gtaattgcca atctattcac acacttttgg

6901 tcggtagagc acaataaaaa tcgatttttt tcatgccgtt gtggtgcagt ttggactata

6961 tcttcatctt ttcttaatca tacccgcttt gatgcgcaca atatcctttt ttttagcctg

7021 cattgaccgc gaggccaaag gatttgcaaa cactaggatc atgcacccat cacttcaaat

7081 taagccggcc aaataggcat caagcttttg tttgcttgga tggcaaaagt aggtttggcc

7141 agagatgttt ggcgtatagt ctctgagggc aaaccatcat ggttcgttcc ctgctgatcg

7201 tctttgcaaa gttcccagca tatagtcaaa ttcggccaag acatcgctgc cttgtcaggc

7261 gcaaatacac ctgccaatac tgggagagat aataaaagta ggaatgctgt cactaatacg

7321 gaccatacga ataggggtaa tctatgcatg gtcattcctg gcccacgcat gttaaaaata

7381 gataggggtc agtctatgct gccctccgaa ccatacatga caattttttg tcatacagct

7441 ctcactcgga aaacttcaat tgctgagatt cttgtatcag gtgcgtttcg aaggatgtat

7501 tacttaatag aggcctagga ctgatagtat gatattatct gcatttccta gcttctttgc

7561 atgatacgct tcgtggtgag cttcacataa tggaatctgc tttcgtttct atgctctggg

7621 caaccgggta accttagttt cttattcgaa tttttccttg aacgtttaaa acagtcctta

7681 cattatttat ttccatattc ctatcaccgc agatggcaca aatccgacct aacccagact

7741 cgtaaaattt ttcggtctac caaacctaca taacttaatt tggagtagct gtttaccttg

7801 taatcatgta gaaccttata gttatttgga atcgtcagac tactctaagt attcaggcat

7861 tgaagcttag ctccaatttt attgaaccca gttcggaact tcgattttaa agccggcttt

7921 ggatgagtga actaagaacc atatcacttt ttgcagattt cttttatcaa ccacaccaca

7981 ataattgagc agtcctatta attaggctag gatggatatc tggatgtgat agtcttatct

8041 tttaatacct ttggcgcaga ttcatttatg attcggcctt tgcgtatacg tatctgcgag

8101 ttcgaaaaac cagttcccat gcaatctata aggggttggg ccatctgaac ctttttccgc

8161 aaagccaaga aagctggttt tacgagtaat gcccccaggc ttaccttgga tattttcata

8221 gcagtaaacc agaaatttag gatccgatag aattattctc agtccattat agcgtctctg

8281 gttattttta caattgttta ctatcttatt agcatatgta cgggcgtcgc tttgttaacc

8341 attcttctga tttctttgaa gaggcctgcg agagtaactt tttttgcccg aaacagatga

8401 aaaagaacta tggatcgttt tctgactagt cacctttgtg ttctggctgg gttggtttcc

8461 ttctcctggc tactatgaga cctctgagcc cgcgcatcat ttgtcggatg atgtgaagcg

8521 gttacttccc gtggttgccc tattttcgtg ttttctttct gacctttgtc agagccttca

8581 gtagtttaag gtccttgcgc acttgcttga ttgctcaggc cggttcctat gttagaatca

8641 ctcgtggctg tccaacaact atgaccattc actacatcag tcaaagcttt cgcccagatt

8701 ggtattggtt cctgggttac tctgccacac atataccgac gtattctgct tggtatatgt

8761 agccttttca aggcagtgag tgcgtatcaa gttggttaac ctaaccctta ctaccctgct

8821 taaaagatac caccaaggag ggcagtcccc tttggcctcc ccattgacca actgctcgca

8881 aacatgatgg attattaacc caaaatgccg cattggcctg ctgcatgtat ttccttaaaa

8941 gagtcagaca tacatgtagg aatatggata ttagtcctca ctgaaaacga gcctcgcaca

9001 gcgcactagt gataaaattg atagaaccta aaatagagga aacacccgat aaatgaagac

9061 tgaaaatggc taaatccaca gatcctccag aatgactggt tataccactt aacggcggat

9121 agaccgtcca tccggtacca gcgcccactt ctactaaagc ggaacttaag agaagtaaca

9181 gtgacggtgg taacaaccaa aaactaatgt tatttaatcg tggaaatgcc atatcaggtg

9241 cacctataag aatcggaacg aaccaattac caaatccacc tatcatcgca ggcataacca

9301 taaaggaaat cattaaaaaa gcgtgagctg ttattaacac attataaagt tgatgatttc

9361 caccaagaat ttgattgcca ggctgtgcta attccatacg aattagtact gagaagcatg

9421 tacccatgac tccggcaatg gcaccaaaaa tgcaatatag agtccctata tctttgtggt

9481 tcgtggaaaa cagccatctt tgtgcaaaat tgttcatttc agaactccat ctttcaataa

9541 taccatgctt tgttgaacta gttttgactg atgttttcgc aatttagaaa agcgaaattc

9601 gtcacaaact gtttcgcgaa aacaggtgac tatcttctct tgtaaactgc tgcgagaatg

9661 ctcttgaata gctaaaagtg ttttcaactt ttgctctact tgttggcata aaacagcttg

9721 cactgtctgt ttgcacttag gtgcgcagcg cgtaagcaga tcatttatac aagattctcc

9781 aatcatttgc gtacttgaac gcaaacttat actacgtaat tcatgctgtt tcttcaactc

9841 ggacaacaga gcttcttgag aactcatcaa ttgctgtaac tctgaaagaa gagcttcgct

9901 tcgcgcatca gaagtagctt ttaaagtttt accaaaggtt ctttgactaa atataacaaa

9961 acctacaaaa cttaaagcta caataatttc ttcattataa attaagattt gttttgaact

10021 taaaacacta aaaattaaaa tagcaaatat aataaattca cgcattcgta tttttctttt

10081 ttcctggtcc gttcttgata tttactaggg tgttcctttg tccgcgtaaa acatagattt

10141 tgttaagagc tgtggtttga cgtgacgcta aagaagttat atgataagta gagggactca

10201 taattgaaag tgcgtttttt tttattactt gtgagacact aatttctccc aaggaacata

10261 cataagattt attcagtcgt tttaattgat tagcatttaa gctttttacc attttgttac

10321 accacttgga tgctccagat acacctgagt acagatagga tatactggta tcaaagcatt

10381 ctttgaaaac aacatcctgt tcaacactgt aatcgctcgg atctgtgcct acattttgat

10441 gcgaaatcag ctgttttcgt aatttgagaa tgcgacttat tttgggtaat ccatcattat

10501 ataataatac ataaaagacc atatagaaaa cacataacca aacaaattgc gtcaaatagg

10561 taaattgatc tagttgaggc attttttttt actttttctt tattgattca aatactttta

10621 ttgaatcacg agagaagaaa acaagttttc ttctttgagc ccttaatgtt aaagctcttt

10681 aagcaaaacc tgccaaacgg gccgcagatt ttcataggaa attaaagaag gaaaagttcg

10741 taaaaaaact aaagtcatga ttaaaatata tatatatata ttttgttatt agtattctac

10801 ataacgcgaa gcgaacacca cgattgtttt ggagtctgga cgaaaaaaac tcttttttaa

10861 gcttatagat agataggtta actaaaagct actaatattt ccactactat ccattccatc

10921 atcgaggtat cgagtttcca catgggcata tgggacaatg ataaatcgct tgtagtcaca

10981 ctaattggtc atttacatga catcctttct tcaaacccag ttctttagtt gttctgcgtt

11041 aacacactaa caaaattgaa ttccttgccc ggccttgatc tctgagtcta gatacgttat

11101 ttgtggtgga tcgttccgta ttttcatgcg ttttctcagt gtgggcttga ggctttgggc

11161 ctaagcgctt taagctttgt ttggtctttt gggtcgtaaa gaccataaga aggaataaag

11221 ctggaatttt tatttttttt tttttttgtc tcttcatatt tatgaaaaaa tgtgtttttt

11281 ttcgtgtttt gcaagtgttt ccgctttgcg cctaaacgct ttacttgttt ttgacgtggt

11341 tttgctggcg aagaataatc tagtttgcca tcaccaattt cttttaccac gatattgcca

11401 atttttgagt tcatgttcaa aatggagaag attctccatt gactatgaaa tacttttcga

11461 tttctgcgaa gactctttgg aagaaaagct atatgacctg cgattgctac cgcataacct

11521 cctttgactg aattcaaaat aaaccctttg atcaaattcc ggtcacttcg ccaaatttta

11581 gttagttcag tccaaactag tttgcttttc cattttcttt ctagcggttt tggcaaaagc

11641 atttttggtt caccaaacac ttccacatct tcaattccca aaataaacct cgtttgctta

11701 gtgattggca ctcttttcag ctcatgttgg aaacaaatta ttggagtttt cagccctgta

11761 ttcaccaata tcatgttttg tcgtaatttt ttaactgaac attgtatagc gcttccgcgt

11821 aatagattca aactagaatt atattgggaa aatagttgag taaagctcat gttgcttttt

11881 tctttttttt agtacttatt ttttaacctg attcatctgc ttatagaggc tttcagacca

11941 cgctgcgccc tcataatcag tttcatgagg aatgcaatta catagtaatt gctagagagt

12001 ggggcgaaat tcgggctgct attgttgtgt cctcttacag agccatacat gatagttttg

12061 tgtcatacgg ctttttgctc gaaaccacga aaaaaaaagt atagattttt ttatgttgat

12121 atcatccatt ttctatcacc agttggccta tctcacaaaa aaagagtccg ataccgtcgc

12181 cgcgtgaata cacacgcggc tcttaacgag taaagcctaa ggggctgcga agactgtggc

12241 ctttcattca ttttttttat accaaaaata gaaaaaatgg ctaagtaaca taaaggtaat

12301 gtattggatt gcaaatccta gaaagatggt tcaaatccgt ccttagccta ctttaaattc

12361 cactgtttct ttacaagtgc tgcactttat tatttaaaga aggtcaaaaa ctttgatcaa

12421 cctctatact tacccgccat ctgcaacaca gacaatgcaa gcaacaaccg gctaagcgcc

12481 cgcggccttt tggcaaggcc ttgtttcaaa ctttgaggtt gctttttatc gaagataaga

12541 agcaagataa gtaaaatata tatatatatt tttttgtgaa acagtctcca gaacgaagca

12601 tgcttttgtt taaagccact gcaagatcga ctttcagacc actttattct ctcaagatcc

12661 aaactcctga aaaattattt aatataaagc ttcactctat tgtgtttaga agtggatttg

12721 aaccactgac acaaggattt tcagtccttt gctctaacca tctgagctat ctaaacagaa

12781 ataaaaaaaa aggaactatg tacaattcta gtttgttggt tattcaaaaa ttattaagta

12841 caaatgcata tctaggccat cgaataccta cttccgattt tcaaggatat ttatacggat

12901 ttagaaatga aatggctatt attgatttag aaaaaacact tatttgttta cgaagggctt

12961 gtaatttgat tgaatctatt attcgtgcaa aaggccattt tttattagca aataccgatc

13021 cagaatataa taaaatagtt caacaaatgg caaaaaggac caatcagagc tatatcaatc

13081 ataaatggat tggaggattt ttgaccaatc gcaaacatat gaaaaatgta caaaaacact

13141 ttcagaattt ctctgcgcat tccaaattta aagacgcctc tacatcgtcg cccttcgatt

13201 tctttccgca ttttagaaag atgcaaaaat gttttgaagg aatcatgaca cacgatattc

13261 cagattgttt aataataata aatgcaaata aaaattctat ggctatactt gaagccaatc

13321 aattacaaat acctatcgta tctttggtgg attctaatat ttcaaacaga ttacaaaaat

13381 taataaccta tcccattcca gtgaatgatg attctataca gtttgtatat ctattttgta

13441 atttgattac gaaaacagtc attctttcac aaagtgcttg gccgctctcg cgaaaaccct

13501 taagtcgagg ccgcaggccc tagctaaaaa aaaaatatat atattttttt ttcgaattga

13561 agattaagaa aaagaacctt gaaactcttt ctaaaacttt tttatcaaca gattttactt

13621 aattcatcta cactaataac gactttttct ctatttctgt catatatcgt agtcacgccc

13681 ttaatgatag gcttttctaa agacttctta tgtcattttc attcaggttt aatttggatt

13741 tgtttattgt tttcttttct tcctgaacgt tttcttcaga atgatttcga agatggtaca

13801 ctcgaattgt attgttcaag cggctattgt ttgcaaaaaa tattactttc taaattggta

13861 ggtcattggg ttcttcaaat aagtggtatt tttggtactt ttccagtggt acaacttcta

13921 taccaatttg atcaactcaa aatgaattgg ttcaccctta ttataggaag tctgatattt

13981 actcttatgt gtggtattca ttcttgtttg gctcttggaa taatatctca tagttggaac

14041 agtttgcaaa atcttaccac tttacccact ctattaccct taattatctt ttgcgcctct

14101 accgaaacag aatggtttca tgttctttta ttaatgggat atttactttt gtttttattt

14161 ctttatccta ttttggtttc aattacttta caaaagttga taagccaata gaattgcttg

14221 cctttgcggg tacaccggct cactcatcgt aaatattgtg gagcaaacaa aaaaagaata

14281 tatatatatt cttttgaata tatatatatt cttttctttc tgtatttatt ttctatacta

14341 gactcctgta cactgtttaa ttctagcaga aggagaaagc agggatttgg tgaagtttga

14401 gtaagaaaac tatttctagg tggcccagat gggaatgatc cagcttagca gagcgcaaag

14461 cttatttttt tttgcattct agatgtctta ggaaggctta ttagtaaagc acttcaaagc

14521 gccgcgctca gcgaagaaaa tttgtttcct tgctgggctg gctctttttt ggcaggttcc

14581 cacaaagcaa agagcaaatc aagtagaaaa aaaagctgtc gaattttaat aattagcacg

14641 aaatgatcca tatttttttt ctagctgggc cattgatgga ttattatttt atgataaaac

14701 gttagaaaat ccctatgtat ttcgaaatac tacgacctta tttgattatg ttatgccctt

14761 ttcgctatgc acgaattctc attggatttt gttggttctt agcagcaatg gctatttatt

14821 taagtatttg ggtagcacca tccgattttc aacaaggtga aaattatcgc attatctacg

14881 tgcatgttcc agccgcttgg atgagtttac ttatttatat tgcaatggct atcagcagtg

14941 tgttattctt attaacaaaa catccgcttt ttcagttatt ttccaaaagc ggcgctaaaa

15001 taggtgcttt gtttacattg tttaccctat taaccggggg tttttggggt aaacctatgt

15061 ggggtacctt ttgggtgtgg gacgctcgtt taacctctgt attaatcttg ttctttattt

15121 atctaggtgt actgcgtttt caacagtttt ccgcggacgt cgcttctatt tctatttgta

15181 tagggttaat caatatacca ataattaaat tttctgtaaa ctggtggaat acattgcatc

15241 aaccttccag cattagccaa tttggtattt caatacatat ttctatgctc attccaatcc

15301 tgttaatcct tactagcttt ttttgtttaa gcgggatttt ttttattttg gaaacacgtc

15361 aaattatttt atctttttct agtttttccg taaaaagtca aataaattcg caaaacaaca

15421 atagaaaaca ggtttttttt tatacgaaca atagatcaag caaaagcacc taaggaaagg

15481 tggactttta ttgggtttaa gcaagttgtt caaggccttc taagaagcaa agcaacgctc

15541 tgcgttaaaa aacgcaaaaa aatttatttt tttgccaatt ataagcaaaa tttactgaaa

15601 tgtataatga attgggccat tattttttag taccgagtat ttttgttgca ttaacttaca

15661 acaaaagacc tgcggctatt tcactttatt tttttctctt tactatttcc ttttttggta

15721 ttttgtcctg ctatatttct tctgattttc ttaattacaa tgtatttacc aactcaaatg

15781 ctaatgcgcc tttattttat aaaatatcag gaacatggtc taatcatgag ggcagtttgt

15841 tattatggtg ttggatccta agtttctatg gatttttttt gggtcatcgg gttcgaccct

15901 gcaatgtctc aaaacgaggg cgcagcaaaa atatattttt ttttcgcaga ccgcttgtgg

15961 cttttcgctc tgcttccttc ataaggaaag agaataaaca attcagacat catttgttgc

16021 agaacctgtt tggtaccata aatcataaaa gctccctcga gagccaatcc gaagtgacgc

16081 cctcaggtat cgttcaagag ccctcgaata aaaggttaaa agatggtaca aatgcagaag

16141 aaaataaaag gcccataagg cttaaagaat ggaaagagtt gaaaaaaaaa aaatctagat

16201 tttttttttt gctttacgcc ttatgtaaca atttagattc tttatttttg gcacaaaatg

16261 caaataataa agtttccttt attgatgaac ggcgaattta tatgggcatt gctttatttt

16321 tttcgatttt tttattagca agttccaatc cttttgttcg aatttcattt gtttgtacta

16381 aatcacttgc agaattaaat cctgttttac aagatcctat attagctata catcctcctt

16441 gcatttatgc aggatatgtc gccagtgcta ttggtttttg cttatgtcta gccaaaataa

16501 tgaatggtat ctctgcactc tatttgccga tgcgaaggga aagcaaggcc gaaatttttg

16561 atgctttctc tcgcataaca aataagctta ttacccagga gaatgcaaaa aaaattctaa

16621 aaaatatatt tgaaaatacc tgttctctcc atcccagttt tgctttcatc ttgcgtagca

16681 ataggagcct gctcgggctt cggcacttgg tgtcgccttc tccgctccgg tcaaaagagc

16741 ggctgaatct tacaaagagc cagtggacga agcgtgttgt tcgtaaagcg aatactgcgt

16801 ttttgcattt tggttggacc cgtagcgcga ataaaggagt ctctggccca caatgccatg

16861 ggtggaaaca aattcaaatt tggatcttga catgctggtg ttttctaact gtaggcatat

16921 tgctaggaag ttggtgggct tatcatgaat tagggtgggg gggttggtgg ttttgggatc

16981 ctgtagaaaa tgcttctttt atgccttggc tattagctac agcttgtatt cattcagtaa

17041 ttttacctaa attgaattat tggactttgt ttcttaatat ggtcactttt ctatgtcgtg

17101 ttttaggaac ttttttcgta cgttctggat tgctaacttc tgttcatagt tttgctacag

17161 attctacacg aggaatcttt ttatggtttt ttttcctctt aattactagc atatctttga

17221 tgtttttttt tcaaatgaag cagcaatcaa gtactaggct agttggtgca ttatctaatt

17281 taccatcgaa tcaaagcctg aaggccccaa aacccgtaaa ccagatctta tggtattcgc

17341 ggcgaagcac tctattcgtg cacttgcgta aatttactcg tttatcaaag ctgatggagg

17401 acgaagaagg ccatgacaaa ctaattgtat acaaagcaag taaaatacat aaataaaaaa

17461 agctcttttt ctcgggctag agagacaaaa agctagcaac atttcgaatc cacacggtga

17521 aacctttggc ttttttgtca tttgttatgc gaagacggaa aagcagaatc ctaaaaagaa

17581 atagagcaga tggtccaact acagaacttt tttttttttc ttatgtttat ggttgtgctt

17641 tgtggtacgg cagcacccat actatttcaa tggttggtaa gtagagatgt tcccacaggt

17701 gctccttttt ctcatggtac tataatacct atttttacct ctttattatt gctcctagtt

17761 catgtacatt ccaggggatt catacgctct atggagaaaa cagaaagttt cgttttggta

17821 agagcaaaac ccattttctt actcaacata attgaaaaaa gctccccaaa aactagagct

17881 aaaaatgcat tttttttctt ttctcttttc atttccaatt tttccatttt taaatttatg

17941 ggagacttgt catatttaga atctttctgt ggtgtgcttt gttttttatt attctgtaca

18001 ttttttttat cattcaaata taggcgcgat acgtgggcaa acaaggagcg taggcttgaa

18061 atagaggaaa aaagaaaacc gcgtaagcga gcacagagaa gaaagcgcca agcgctttgt

18121 tggcctaaca aaagaaagaa acaaagaaat aaaaagaaag aacattttta ctttttctct

18181 ctttcaaata aatcaaaaat atttttgatt tatttgctgc aatttccaaa aaccttcggc

18241 ttcaacgaaa aagccaaaat tttggctttt tattctctgc ttgctttttt gcaagcttat

18301 tctttcgtcc tcgaaaatat ttggaataga ttttttcttg ttcgcgcctt accaaaaaga

18361 ctgatggatg ttggtcatga ctttcgaaaa gttcccatga ctatgaaaat ttcacatgga

18421 ggagtttgca tctttattat gggtgttatt ctgtcgtgcg acccggcagc ttatgcgcga

18481 tcatctcgtg tttaagctca cgccatgtgg gcgtgaactc tggttgaatt cgggttttaa

18541 atcccgccgc tgagatgctc agtcgactct tgaaccttga taggaagacg gcttcttcct

18601 aaattagtgc aaaaggttca aggacttagg atgaacttaa tgtgaatgag tataagcttc

18661 gctgctcaaa aacacccagt attgaccaca ctgagagact tgccaatggc aagaaaggcc

18721 gcgggtaacg ctagttggcg aaatagcgtt aagcattcct agcaatacgg aaagagaggt

18781 cgtgatgata tcatctacgt tcgtactgtt cctcgtggag taaatctcac atccaaaaat

18841 ctaaccaggg aacggaataa ttcccattaa gttccagtaa aactggcagg ccagccgggc

18901 cgtaagctag tgggaacagg attcttccga aaagacaaga ccttcggggc aaacgctcac

18961 tttgctcgcg ttagtaaagg aaatctcgaa gaaaaggccg acgcggggac tcagggcgca

19021 gcataacgaa tggtgaagag tccatagaag caaaataaac aggaaaaaaa gatttttagg

19081 cgaatgccat gtaaatttcc gctttattat ttattattcg gttttcaggt tccccttgag

19141 aagagccgta tgaggccgta ggctcacgta cggttcggaa gccaagctcc tgcagtgatg

19201 ccgtggctta ggttaactaa tacaaaaaag agacagttta ctcaattatt gcctttaggt

19261 tctgaactac atataggaag agagcattgt tgtttgcgag gtattgatca attacatgga

19321 cccacctttc attccatttg tggtaatttg attatttata aaccgtcctt aaaaaattca

19381 ttcatttttg attatgatga atcacttcgt gccataatcg acttgttgcc aattgcagcg

19441 ctttcgtacc agaatgaaaa agttgaaaaa aaatctatag attttttttc aacttttttc

19501 catggtgaca gatcatggag aaatcgcgaa catcatagtt tcccactttg gttgactgtg

19561 ttcccagaaa aaagattttc tttttctaat caagaaacaa gcactaccaa agtggctata

19621 catagtaatc tttttacgga tctatatgct ttaattggaa ctggaagttt tgaaacaggc

19681 tggtatatta ccataatgaa actacctttc attttctgta tttggatagg ctttattttg

19741 gcttcattgg gaggcttgcg tagttttcta cgtcagctgg ctttatatag attggattgg

19801 aattgaaaaa aatatatata tatatatttt ttgtataaaa tgaaaaattc cataaatctg

19861 gagtaaaagg attcgaacct ttgcctgtcg gtatcaaaaa ccgaagcctt tccacttggc

19921 tatactccaa gaaatctacc tacggccttt tttttaaagc ttgtaaagtg ctacgcaccc

19981 acctaaaaca aaaacgaaat aacttcccac tctgccaatg gctcataacg gaacaacgta

20041 ggggcggtta atttgtttat gttctcccac aatatacaat attttttaat aatcgaatta

20101 ttcatctata tcgtgaatga aggacctata acttcaagcc tgagctgttc tcttatgcat

20161 acatagtaaa taaatagagc acataatagt ttataatctg atttatgaat tgagcacact

20221 tcttatgaat aaacgtatat tattttttcg ccaatcaagc agtatggctt ctcttacaat

20281 tcttaaaaac agtttgatca cgattcgtgt tcgatccgca aagcgagagt acggatacta

20341 tgcgaggtga aagacccatt gatttacaaa tttatgatat gataataaat ttcctactag

20401 tagtcatact ttttttttgt taaatgtcgt ttgttctaaa gatgtctatg aatttaggtg

20461 aaggccgcag ccgtaaatct ttaataaaaa aattcaaaac atcataggga tttatatcta

20521 tagataaagc aatctggtta cacttaatct tgatatgggt cttagaccta accactcgaa

20581 ttactaagcc tgtttttttt taggcgtcaa atacacaaaa ataaccgcgg tgattagagg

20641 atagtgcgat cactgcggtc cctcccgcac aagtaggtta ggattagaaa atgcatgtca

20701 tattaatatc aaatatatca caatgatata tttaaaaaat aataatgcta aacaaatatt

20761 tgtttctgga gccttgtaac ttccgctggt aataatcata atttaaggaa agataagttt

20821 ctgaaaattc tcgtcatagc cttatgtttt gcgataaggg cagagtttag ggttagcttt

20881 aagcttatat tacctaggaa aaatcgtgga ctcattatgt tattttatcg ccatcacttc

20941 ataatattgt catacttggc gcttactgtg gattgggcac ctttattctt catttctatt

21001 cggtcgacct gcgcgtaaat ttgtggtcac ttcgtgattc aacttccatt agtcgaatat

21061 gccgggtgca gctcgacgat ctaccatagc tggtggcata ctatctcaag aagcagtatt

21121 aactaagaaa cagtaattat atagtaggct agccgaacga ctaaagggct agtgatcgca

21181 gaacttgaag ttccgacttg tacggataga gggactcgaa cccccacaaa cattatagtc

21241 accagaacct aaacctggca tgtctaccaa ttccatcata tccgccaaaa tttgattcaa

21301 tctatggaaa tttttttttc tttctttagt tattagactc tcttaatggc ctcaatacca

21361 tttcagatgg tagcccaagg gcccatggat tgccagattt tttatcgtcg atcaataatc

21421 acagtcacat gaatgggtca aagggcctct cgtcaaacta gaattgaact tacaccgtca

21481 tatttggcct aaccctgtag gttaggttgt gttttatggt ttctgaatcg tgcctgtaga

21541 taaagttatt tctcatggtt cgtcactgta aaaataaact aaactagatt tacttattaa

21601 tgatccataa gtcatattgt tttttgcgtt tgcgggtgta ttatctaagc atcgtatctt

21661 tttgactgcg tcgaaagaaa gagggcgaag cggttctttg ctctcgcgca gtgaaccatt

21721 agtgccttgt aggtcatttg attggtatac cggcgatttt attatgttat ttcttattgt

21781 tgtccttgtt atattgtggt tgggcgcgtg atgtacaaaa acatgcaaat ttttgattta

21841 ctcaatacta tacagattat gacatctata tatttcggtc cagtgcaccg tggcgcgttt

21901 cgcgacatgg ctcagtgttg cgcctcgagc taagccacgg cacgatacgc gctgtcccgc

21961 tgagtaaaaa tctcctcaag ctcttcgggg tactgcccta tttttcggct accaagcaca

22021 gagccaccag ctgaagatca ttactttttc tcgaatgaat catcataaat aatgattata

22081 tatttttttt cataaagaga atatcttgtt ttttgcttga ttctgcaaaa taattacaca

22141 acgttaagat ctgtaaaggt tacatgctat tttgttttaa aaaaaaaagg ttaactaatt

22201 accctactta cggatggaga gggattcgaa cccccggtat tcccaatact tcggttttca

22261 agaccgactc tttcaaccgc tcagacatcc atccttatct taataagatc atcggctcaa

22321 aggaaccaat aatcaaccta atattaattt gctatgtaaa tggagattta aagagaaaag

22381 cgagaaaaaa gaaaaaaaaa ttttaggcgg atatagatta aaggtaaatt atctgccttc

22441 caagcaggag atatgggttc gattcccgtt atccgcaatg gctaaaaaaa caaattaaac

22501 ttcatatgcc tgcatcaaga tttaaaactt gtcgtcaaat ttcggaaaat gtttggcaaa

22561 ccaaaaaact tactcaaaaa caaaaagcca ttattttgaa gcttcgaaaa aaagcaaata

22621 aaaaacaatc tgactttttc aaagaattac aaactataca aaagttatcc cttttttatg

22681 gaaaattacc cattaaaaag atgcaaaggt ctaaaacgca gacttatcta gataaaaaaa

22741 acagtttgtt attcgatata gaacgaagat tagacgtgat tctgattcgt cttaatttct

22801 gtttaaccat ttttcaagca aggcagctaa taagtcataa aaaaatttgt gtcaattata

22861 aaatggtcaa tattcctggt tttcaattat ctaagggtga tcttatatct attcaagata

22921 attttctata tttcattaga tcaaaaataa gacaaaattt tcagagtaac cgaatatgga

22981 gaataaaacc tactcattta gaggttaatt atagaacact aaaagccgtg gtattatatg

23041 aacctcaaca aatacaattc ccttatagca tagatctaga ccttcttgat taaagcagga

23101 acgtatgtaa attatttatt ggcagagcga taacagagtg aatctctcgt agatgataaa

23161 aaaaattccg ggaatctttt gatttttttg aaccactttt ggctctttgc tatagacata

23221 aagacaatac tacgattgcg caaaaaaatc aagtaaagct cgtatgccca ggcagacgga

23281 gcattcgttt tatgacgagc ttttttctcg gcctcgtatt atttttctgt gtcttcgagg

23341 ccagagacgg aaaaataagc ggggaattaa gtccgctttg tagtgtggag tgaaaaatac

23401 ttttgtttgc tgcgccctgg ctagttttgt aacggctata agcgagcctg gtctcatatc

23461 atatcgaatt ggcgaagatt atgggcgccg cagctctatt tcggcgaagc gcttatttgt

23521 tttttgatgg cgtcgtcacg gtcgctttgc tctgcttggc cggatccaaa tcaaccataa

23581 agcatctagg gtggaggagg gcagcgcaaa caaaagctat cgggcttctg cgcgtcctct

23641 tcctgcatca gcagaagaaa aagaaaggta gccgggaaag gataaataga tggttaaagc

23701 ttaatgcata gcagcaagca aaattaggcc aaagatcaaa aaaaatctat ctatattttt

23761 gtttttcgtt gcgccccgcg gcctggcccc tggccatggc ccaatttcgg ttaaaggact

23821 agttgcttct tataaacttg tataatcaat gcgtaaaaaa tggtcaactc tattatacaa

23881 taaataagta aataagcgac aatttgacac cagatatctg gaggtgttag aaaagctgct

23941 gtaaaaatcg aaagaaccat aaaaaaacga cgatttttta tgcagctttt cacaaaaatc

24001 ccttttgatt ctagcaaaaa gatcacaagt acctgtactt gagagcatat tgatgaaata

24061 aacaaaatac gaacagttaa taaaatatag tcaaaaatct taggttgtaa ctttattata

24121 agcagattag ttgatgtttt attcaattca tataaaaagt gccaaacatt gggaactatc

24181 ccaacaaaag taacaaaaaa gaacaggaag aagcaaaaac cacttaagta aaagaatttg

24241 ttgtattttt ttctttgttc ttcataacag ctaggaatca aaaaacacca gatttgataa

24301 cttaagaaag gaaataaaaa ataaaagcat gatattagag aaatagtaac atacgtgctt

24361 aaggcctccg ttaattgtgt acaaataaaa cctgaataag aaagaataag aaaggatttc

24421 gctgacaaaa aaaacaaatc ttctgaaaac caataacacg taaaccatgt taaactgaag

24481 caaatgaata tccaaaaaaa acgaattcta acttctttca gaatagtttt aaataaaaaa

24541 ttcgtgatat ttcattgtgt gttaaaccta ataagagcga aaaaaccgtt gggttggctt

24601 ttactgcgcc cggcaaagtg tgcaatcaat cgtaaggccc taccaagatt ctcttttcat

24661 ttcattagta gttaagggtt cgcctctaga attttactac atttaagggt actcattcat

24721 cataatgcaa ttactatgta ctaaaaccgt attacagccg agcatttcta tttaattgag

24781 taaaaggcat ggcatagagc gcatatagcc acgggaatct atggctaaat catatttttt

24841 tttgctttat gtatgacttt tcttttattt tcagtgttat tttcacgctg cgcgcccttt

24901 attcgttata cgaagacagc ttttttccct gtagttcacg aatgaatgaa ggttagtatt

24961 gtactgcatt cttagctcag ttggatagag caacaacctt ctaagttgaa ggtcacaggt

25021 tcaaatcctg taggatgctt aaagaaagtg cataatgaat gaatcaataa tatataccaa

25081 cgatacatgc atctatcgat tagttcaaac ccattaaagg ttacatacca tacatacata

25141 cacgcgcgga ttgaccgatt tataaataaa taatttttta tttattttgg gggagagtgg

25201 ccgagtggtt aaaagcgaca gactgtaaat ctgttgaagg ttttctacgt aggttcgaat

25261 cctgcctctc ccatatactc cgtatttgaa gaatagagac gaagcacttg tttttgtgct

25321 tatcccttca tgcaattaaa tagagtggaa ctttctcaga aacatattga atgctttggt

25381 attcgagccc tctccgaacc gtacgagata gtcgcccatc atacggctct ccaattcaac

25441 ctctatttgg atttgctttt gtcgttagat ctttggcctg tttttatcaa gaaataataa

25501 cataaaaaaa atgcattttc attatctcct ctgagctcgt ccttaatact ctgaacatgg

25561 tgcattctat tcagatttac gatataatga aggaagcacg aagagcagtt tcgcagcgtt

25621 ttagtcatca agcaagtgat gccataggat tcttgatagc agaatttact ctgcagttta

25681 gaacgtatga aacgaatttt agatagtcaa ggtagggctt tcgacaagga cgccactaag

25741 ctggcattga agacagtatg gtttttagta tatccctttt tcggttttat attttgcgtg

25801 gagtatctct tccactcata gattgtttcc atgcttccat ctgggtgtcc gtgataccgc

25861 aaaaaaaaaa tatatatata tatatttttt tttttgctta tgaaagtaag tcttcaaaaa

25921 ggcgttttcc attttcggct cctcattctg ctctgttcgc atagcaaatg gcagcatgta

25981 gattcgtttt gtgctgaact ttttttgatt actgtgcttc gttctatagg gctcccagtt

26041 tcggttccat caatggtcct cttttgtttg atattggtgt catcgattca ggttccgttg

26101 ccctaatcgg atataaagca tcattcatga gtttaggttg cccacgatgt tttgcagatc

26161 tgaataggtt ctcccagctt tgcgaaagcg gaagatccaa gagtccattg aaagagcggc

26221 agcgccctaa attgcttttt tctctatttc cagacaaatc ccgtttgaaa cccgtagggg

26281 cttagctaga aaacgtggtt gaatatggtc tgctaaggta cagcagacgg ttaggcccct

26341 tcccttttgt tagcagtttt agcgaaaaaa atctttttac tacgtacggc tcaggcgggt

26401 actatgggcc ctctgccatc cacttcggtc agctggacga aagtggattt caccggtgtt

26461 gcctacagtt tttggccaat tttaatttga ggctatttcg cgtcgagatg tcgtttagtc

26521 ttttgtcccc attaatttgg gtaatctgct ccctcgtgca ggctctgtaa tattcgtccg

26581 cagggtttct ttttccattc tggtcttacc ataatttatt gatggcggac tgagagcttg

26641 gcagcgcgca ctcgtgtgca ttaccacaag gagtttctcg tgattaactg caggtccagt

26701 ttgaccgaaa gagactttga tttgccagag caggggctca tctcatacaa gagcaggcta

26761 gcgtagtggt cttgggttgt ttgggctaga atcattcgtt tgctttgtga acctttaggc

26821 tttgttcgaa gaaaaaagaa aatttttttg ctatgcgctc ttcgctgcct tttattataa

26881 ccaacctttt attcgctaag caaagaagta gaccgcaggt caaacgtatt ttcttttccg

26941 aacgctgcct gtggtccttt gggtgctttt gcgctttcta ggatggcaaa aaagcaagtt

27001 aaaagcctaa aagacatact ctaacatcat gaggtcttcc ttgttttttt tccaactacg

27061 tttttagagc atgctgtggt tcccacccgt ttacacggtg gttgggtaag ctctatgccc

27121 ggcacgcgga atagggtctc gcgtggtttg ctatatggcc gctagcgcaa aactgcgaat

27181 aatttccata tggctaaaca atgactgtag gcgacgcgaa cggtcgccct aaattccact

27241 gcaatagtcc cacgaattcg aaaagttata accagaatgg ccagcccaat agcggattcc

27301 gcagctgcca ccgttaaaac aaataaagca aataattgac ccatcatatc atctaaataa

27361 accgaaaata ccaaaaagtt taaatcgacc gcaagtaaca ttaactcaat tgacattaac

27421 ataataagga tattttttct attcaaaaaa atcccccaaa tacctaaaag aaaaagaatc

27481 atagaaaatg ttaaatattt tactagatcc ataataagag tctctttttt gaaagccaac

27541 tcgggttcaa gccaagcaca tgccggttcc ttagccaata agtactgctt tgctcttttt

27601 tttatggcag gggcaatata aaccagaaca agcacataga ggacaatgaa aaaaaccatc

27661 attagtaacc atttaattac ttaccaactt catccatgac acggcctcta ctagcaccag

27721 aaaaaaaaat atatatatat ttttttttgc tgcgctctcc gcgcttattt ttgccctata

27781 gcactatctg aatctttaga tgattagaaa gtttttttaa aagacaaaaa acaaaaaaac

27841 atatttgata attattaaac aaaatactct gaaaagaatc aagatccaat ttcgtgttat

27901 ttcatggtga acataatctg tcagaatttc ttcaattccc acatgaatat gccagaataa

27961 caaaatattt aacagaatca aagtagaaac atttgatata agaatggttg ggattagaga

28021 agcggcagtc attctttgaa gaagccaatg ccctaaggtt tctctatgag ctttcattgc

28081 tttttacctt tcttttaaga gtaaaaggaa actggccttt ttggtccggc cccttcttat

28141 agaagcgtat gtgacaattg tccgtcatac ggctctgcct atctaaaaag tatcttgtta

28201 gccgaaatag tactggaaca ggttgtaggc ttgtcttagt taagccttcg caagataagg

28261 tagacctgat tccgaggcat cgctgagcta tcagggaaaa aagtatatat atatatactt

28321 ttttttcgag gttatcgtat ttcgtgctta tgagaaatag aatcggataa tattcgatac

28381 agctaaaaaa gctgcacaga ataacataat aattccggaa gtatatactt tggataattc

28441 tagaaaaaaa cctagatccc acaataaatg acgaattcca ttactcatat gatagcacaa

28501 agccaataaa ctgaaattga ctacgcttaa gattaaccaa taggaatagg aagtgaagaa

28561 aaaagcgtac cggtaaagat aataggaagt aaggttaaga tcgcctattt ttaaagaaag

28621 aatactaaaa aaaaccataa tggatagagt caaacttcgg taggaagtta tgattaactc

28681 ctacctacta agtgctctcc gaaccgtacg tgatagtctc ccatcatacg gctcactaac

28741 tctcctgatt tgactcatag gagacgggct ccattgactg cggctcgttg ggtgcctgcc

28801 cttcccggct accgattgga ttatcaatgg cactggatgt atcatcatat ataatgtatt

28861 aacatcttga tgttaatagg gcgcaacaaa aaatagatat atttgccgtt ttcttttttg

28921 agttttggag agtaaaacct gccagactag gcaggtgcat agaccccttc gccttttatt

28981 caatccgcaa gtttcctgtt tacacggttc ttttaggatc aggcaggtac tatgggtcct

29041 ctgacttcca actcaaacca tagtgtatgg ttggatctcg ccgatatcac ctgtgagcta

29101 tagcgtttga gatgtcgttt agttctctgt ccccattaat ttgggtaatc tgcttccatg

29161 cgtaaaaata tatctatttt tttctcgtcc ttaccgttct tagccgccag caggctgaaa

29221 gcataacagt gttcattcgt gcgattcccc gcgtgcattt ttttcgcatt agttcgctgt

29281 agggtaggct gacccgaaaa gaactgcgat tctgctttat catctcatgc taaatgaaat

29341 atatatatat atatttcatt tagcaagcgc cagcggactc gcggaggatt attgagtagg

29401 ccgataaact agccgacagc cgacgaatat ctcctttatc gctgcttttg tggcatgctt

29461 cctttttttc gctatcgggt aagccctgag cccctcgagc agtagtgcct ttattgtaat

29521 cacaagtgat ctaacggccg cccctaagaa agccccagaa attctatgga gaatagaaag

29581 agtagaagta agctgtggct tataaatggt aagatgaggt gataacggtc gattgatttt

29641 catgttttta gttaactttg attttgactc aaggtgacag gatttgaacc tatgaccctc

29701 tgtacccaaa acagatgcgc taccagactg cgctacacct tggctgatgt tccctaatga

29761 atatttgata aatgcttaaa ttgttattga gcaacataca gaaaaaacta aaactaataa

29821 aaaaaactat atttttaacg ccactgaaaa aaagcactac catctcgttc agtttttttt

29881 ttgcctgaaa atattttttt ggtggacgtt tatgatcgtt tcagcccttt aatgcctgct

29941 cgaggccaga agggcaagtc ggtcattgtc cgccttattt ataaagcttt ataatgcaat

30001 tacatgtaat tgcattataa agccaagtat tcaacataat atattattaa tcttttagtt

30061 ttgttattaa ttaagtaatt ccatgaggaa tagccatgaa taatcatgga tagagaaaag

30121 gggccgggtc aaaaattgcg cgattaatct tgcagcccaa aaatagcatt attatcaaga

30181 ctgggaagaa taaaagccac acagaaccga aatgaggctt tcctaggtaa tagctccagc

30241 tcactgtgga caactagcaa tgtcgacaga gccttagaac aaaaaaaatg aatttaggaa

30301 ttcggatcaa tagcaacaag gcaaacaaat ccgcggggct accctgcaac gaagaccgtg

30361 gtattctata gaatatagca tacttagaat ctgcaatcac tatgtaattc cataatgcga

30421 aagaagcata gcaagtttat tggaccaaaa gttctacata gtgaatgcat tttggtttat

30481 gggtagtaaa ccttagtatg atgatgattt agttggtgat acgcagaggt tttctattgg

30541 aagtttggta ggttcaaaac ctacttctgt gtaaaaaatc atactcaaaa aaaagagttt

30601 gatcctggct cagaatgaac gctagcgata tgcttaacac atgcaagttg aacgttgttt

30661 tcgaatcaaa atgaaaacaa agtagcgaac gggtgcgtaa cacgtaggaa tctgccaaac

30721 agtttgggcc aaatcccaaa tgaataaaag ctaaaaagcg ctgtttgatg agcccacgta

30781 gkattagsta gttggtgagg caatggctta ccaaggcgac gatcagtagc tggtctgaga

30841 ggatgatcag ccacactggg actgagacac ggcccagact cctacgggag gcagcagtgg

30901 ggaattttcc gcaatgggcg aaagcctgac ggagcaatgc cgcgtggagg tagaaggscm

30961 acgcagcttg taaagctctt tcatcgagtg tgcgattgtg acaagactcg aataagaagc

31021 cccggctaac tccgtgccag cagccgcggt aagacggggg gggctagtgt tattcggaat

31081 gactaggcgt aaagggcacg taggcggtga atccggttga aagtgaaagt cgccagctca

31141 actggcggaa tgctttcaaa accaattcac ttgagtaaga tagaggatag tggaatttcg

31201 tgtgtagaga taaaattcac agatatacga aggaacacca aaagcgaagg cagctttctg

31261 ggtctctact gacgctgagg tgcgaaagca tagggagcaa acaggattag ataccctggt

31321 agtctatgcc gtaaacgatg agtgttcgtc cttggtctgc gctgtatgca aaacggctga

31381 tcagkgsysy agctaacgcg ttaagtatcc cgcctgggga gtacgttcgc aagaatgaaa

31441 ctcaaaggaa ttgacggggg cccgcacaag cggtggagca tgtggtttaa ttcgatgcaa

31501 cgcgaagaac cttaccaggg cttgacatry srwkaagttt gcttgtcctt aatgggacgg

31561 tacgaaaatt tatacaggtg ctgcatggct gtcgtcagct cgtgtcgtga gatgttgggt

31621 taagtcctat aacgagcgca accctcgttt tgtgttgcta agacatgcgt tttggggtcg

31681 attattgatc atttgatact gacaaagacc acgcaaagaa tatcacaacg ccttatggct

31741 acgaccacac ggttgggtac ttcgacgagc acagctctca tagcgtggca cacataacaa

31801 tgtggcactc agtctttgta aacataattg acaatccatg ccatgtactg cacycwmaas

31861 agactgccgg tgataagccg gaggaaggtg gggatgacgt caagtcagca tgccccttac

31921 gccctgggcg acacacgtgc tacaatggcc gggacaawrg gwagcgaggc tttaaggcag

31981 agcgaatcca gaaagatagc cttagttcgg attgttctct gcaactcgms wgcatgaagc

32041 cggaatcgct agtaatcgcc ggtcagccat acggcggtga atccgttccc gggccttgta

32101 cacaccgccc gtcacactat gggagytggc ywygcccgaa gcatcaaacc aaggatcacc

32161 cattattcca gtgttctacg ccgttggtga gtgtggtcta ccagagtaat tggtggtcta

32221 atgtgggata ccaaggtggg gcctttgact gaggtgaagt cgtaacaagg tagccgtagg

32281 ggaacctgtg gctggattga ctcctttttt taaacaaaaa aaataatggt ttttcgctat

32341 atctgtagcc aaaaatatac atctatatat tttctgtcgt tcggttatgt ttgatagtaa

32401 cacgaataag caaaaaaaaa tatttccttt tgtaaatacg ccctgtgctt ctcattctct

32461 ggactattat tgcatagccc aaaggtaaag tggccagcat aatgcaatat tagcctttaa

32521 tttaaaggct tgagtgaatt tttggtctat atcagttagt gagagccgtt gtactccgca

32581 gcctttccaa ttacgcctgt gaatgtcctt actattatac aataacggct tcagtcacga

32641 ttatgaatat cactaaggcg cagggcgctc taataatcta ttccgtttga acttcataca

32701 taaaaagttc cttctatcgt taagccctat cggggccagg ttaaagggcg catgaggcga

32761 attatcatcc aacagccagg gagcggcaga ttggtataac agcggattaa taattcgcat

32821 gtcggaatag tttagtcggt tagaacagcg ggatcataat tcgcacacgg gggttcaaat

32881 ccctcttccg ataggaactt tcggataaga attgaaccta cgggaggcaa aaaaaagata

32941 tatatatctt tttttgctgg tcactaacct tatcctaaat ctccttcttc tatgataggc

33001 cacggtaaag aaagatttac gatgtttctt aggataacta gaaatgcaac ataatgaatg

33061 ttgttttgac aaaacgatca atcatagaaa gtacaccttt aagtttaatt atagattttg

33121 ttcacgaact aagtggctat actcttatgt tatatcaaca aagtagaatt agccgttatg

33181 ctagtagttt atgaatcatc atagataatt cattattgta gctccgcaaa tgggagcgcg

33241 cgcgaatgta tgttgctccg gctcgttgca agataaattt aagtaggtct acatggcttg

33301 ctttcgatcg ccctattcgt acgtaaggaa taagtcaaga tagaagtagt aagaaagggg

33361 cagtaaacaa cattataaca tagggcaggg ccctgtgatt agacaagcca tgtagaaaac

33421 aaacacggct aggagcatct cgagaagcgg tcgtatctta tgtaagttat aattaataat

33481 tagatttatt tagaaatcta aagtaaatct gcatgttata tatatgccgt gacttaaaaa

33541 tacgtaaaga ctaaaattcc atataacggc agcatcaccg gcggcaattt gatgtagccg

33601 acgcgtggcc tgcggccttg cggggccgga gcgctcctca gcggccgcag gttcttctct

33661 tagggtttta taagaaatcg aaataaagtc aagttagaga gccttgtgat cggcgattct

33721 cgccggagag cacttgtgta gtctacctta ttgaacgcag gaaacctacg actgagcaag

33781 ttcccttttt actctattca ttaatcgcta cgcgcttccg ggcactatgg taagacgtga

33841 aaacacccga tcccattccg acctcgaaat gtgaaatcgt cttacgctat atgtactgat

33901 ttataaattt cgggagacat ggtccaggcc cggacaacgt ccaatttaaa ttgttctaaa

33961 acgctattga aaatgatgaa caatcaatcg cgaggccgaa ggtcctagag gactaagctc

34021 gccaaatatt actatgcaac actcggcgcc taccgatgaa tcatcataga gactcgcaat

34081 aaaaaaattc catatttcta ataaagaact atacttgggg cgggcgggga actgctgcgc

34141 aaaaagaaat attttgcggc gccgttgaag aaaaccttat tatgtgcgcg ggatagagta

34201 attggtaact cgtcaggctc ataatctgaa tgttgtgtag gttcgaatcc tactcccgcc

34261 aaatatttta agtggttttt gtaaaatagc gggatttata caaaaagcag cagtgcatcc

34321 ataatttggt tatttctgcg cgttcttgat ctatttttcg gttataagaa tattaaaata

34381 aataaatggt gtgataaggc aaatactcat tatattgtgt ctaggcgaaa ggaaccctgt

34441 attctgcttg taatgcgaat gatgcgatat agaaatagaa gatacgtatt attctattga

34501 tgatctgaaa tagtcattta caggataatg aaggggtgaa tcgatccagt tatttcaagt

34561 ggcctggatt ttcttatgcc tttttttttc catcagatgt tctttattct tatccttttc

34621 ggaaaaatat gtacaaaatg cataaacctt cgtcttcaag cctgaatgca cccgctccag

34681 taaatgacct gactagaagg caagcccgcc attctgaagt atgaatagaa gtctcattgg

34741 cgaatatttt ggtagtgact agtgaggtag gtgcaattcc tactgtatcc aaaataatgc

34801 atcggatgaa tgcctaggca ttgagaagga aggacgcttt caagggcgaa acgccatggg

34861 gagataccgt ctgtgatcca tggatctcca atcgggaaac cgtatccaag cggggcggcg

34921 cattagtgtg ctgcgctccg tcttggactt tcaaaactta gcgaactgaa acatctaagt

34981 agctaaagga aaggaaatca accgagactc cgttagtagc ggcgagcgaa agcggattag

35041 gctttgtgag aaggccgcag tagaaagcgc attgcgcttt ctactgcggc cttctctctt

35101 catccaattt gtttgaaatt gaatgatgga aaaaccattc aaggaattgt gaaaagattg

35161 gaaaatcttg ccaaagaagg tgatagccct gtaaattttt tcttatggtt cgactcaata

35221 agtaaaacgc ggataccgtg tttgaattat gatcgctctt atgtgaccaa gggggaccac

35281 cytstaagsc taartaytcc tsrgtgaccr atagcgwasw agtaccgtga gggaaaggtg

35341 aaaagaaccc ymagwarggg agtgmaatag aaaacctgaa atccgatgca aacaatcagt

35401 cgaaggacaa agtaggctaa gtctaaatag cgagaaagaa gagattgtgt tctcttttgc

35461 gcttcgttat tcagaacttt ccactctaac ggcgtacctt ttgcataatg ggtcagcgag

35521 taaatggaca cagcaagctt aagccattag gtgtaggcgc aatgaagttg aaagtaaata

35581 aagaatatag ttgtttctat ttgacccgaa actgagtgat ctagccatga gcaggttgaa

35641 gagagctcta acgggccttg gaggaccgaa cccacgtatg tggcaaaata cggggatgac

35701 ttgtggctag gggtgaaagg ccaaccaaac tcagagatag ctggttttcc gcgaaatcta

35761 ttgaggtaga gcgtataatg tttatggcct gaggtagagc actcaatggg ctagggtggc

35821 ccaaagcttg accgacccca aggaaactcc gaatactggc catgattttg tacagacaga

35881 ctttgggtgc taagatccga agtcaagagg gaaacagccc agatcgtacg ctaaggtcct

35941 taagcaatca cttagtggaa aaggaagtaa tcgagcgatg acaaccagga ggtgggcttg

36001 gaagcagcca tcctttgaag aaagcgtaat agctcactgg tctagcttca tggcaccgaa

36061 aatgtatcgg ggctgaagtg attcaccgaa gcgacgagac cttgaaagca cccaccatat

36121 gcaattactg cgtaattgca tgctccgcgg ttacggtacc ctaataggac caaataagca

36181 gggagcacca aagtgcaaaa aaaatctttt tttttcaagt gttagtagcg gaacgttctg

36241 taaatcaaag aaggttgttg gtgacaacac ctggagatat cagaagtgag aatgctgaca

36301 tgagtaacga taaatcatgt gaaaaacatg atcgccaaaa gttcaagggt ttctgcgttc

36361 agtaaatcta cgcagagtga atcggtccct aaggaacccc cgaaagggct taatccgatg

36421 ggtacacgaa agtgacgaag ttgctttggc tactaccaaa ccacgggtta ctcgtgaatt

36481 ggatgattgg gcaggccggt gaacttccaa gaaaaaactt ggtggaattg gaagaatcaa

36541 gattcttcca gtgcaaaacg aaccgtaccc saaaccgaca caggtrrrya rgtagagwat

36601 acywaggsgc kygagasaac ymtstykaag gaactcggca aaatrrcccc gtaacttcgg

36661 gagaaggggt gctttcctag gaaagcggca cataccaggg ggtagcgact gtttattaaa

36721 aacacaggac tctgctaagt ggtaacacga tgtatagagt ctgacacctg cccggtgctg

36781 gaaggtgaaa aggagaagtg ttataggctt cgraysgaag ccccggtgaa cggcggccgt

36841 aactataacg gtcctaaggt agcgaaattc cttgtcgggt aagttccgac ccgcacgaaa

36901 ggcgtaacga nctgsscyrc tgtctccaac atggactcag tgaaattgaa ctctccgtga

36961 agatgcggag taccaacggc tagacggtaa gaccccgtgc acctttacta tagctttgca

37021 gtgacaacct tgattgaatg tgtaggatag gtgggaggtg gtgacgttgc acgtcaccac

37081 caatcttgaa ataccactct ttcgtctaag gatgtctaac tgccgaaaca gaggcgggac

37141 actgcatggt ggttagttta tctggggcgg atgcctttaa gtaaggggcg cttggatggt

37201 aacatcccag gctaatctgg ctatatgctg aaatctccat tttttggaca atcagcaggg

37261 aaggctgaaa aaagccactg cgaaaaattt atttggctcc ctcaacgact gcatgccagg

37321 tatccaacgc aataattatt ggataatgat acagtctgaa cttccagtaa actggaagcc

37381 taaagcactg ttcactacga aacaatgata ggacattcaa actgctaata cgaaaaataa

37441 acaaaaagac taaagaaaaa gaaaatagaa atactagagt gagcacttca attgaattaa

37501 atacaacgac aagtatgttg cagagtgaat gaacataatt gcccaaagag taacggaggt

37561 gtgcgaaggt aggctcgagc taatattatg acagctttag agcgtaatgg tagaagcctg

37621 cctgactgtg agacttacca gtcgaacaga gacgaaagtc ggccatagtg atccgggagt

37681 cccgtgtgga agggctstcg ctcaacggat maaaggtack cyrgggataa caggctgats

37741 wcycccaaga gctcwyatcg acggrrwsgt ttggcacctc gatgtcgrct cwtcrcmwcc

37801 tggggcggta gtacgttcca agggttgggc tgttcgccca ttaaagcggt acgtgagctg

37861 ggttcagaac gtcgtgagac agttcggtyc mtatctaccg ttggtgttta agagagaact

37921 gcgagaagcc aaccctagta cgagaggact gggttgggtc aacctatggt gtaccggttg

37981 ttatgccaat agcagcgccg ggtagctaag ttagtataga agaactgctg aaagcatcta

38041 agcgggaaat ccttctctat acaagttctc ataaaaggtg aaagaccatc actttgatag

38101 gcgagaggta taaacaccgt gaggtgtgaa acaaggctta cggctcttgt tgagcttact

38161 cgtactaatt atctacttct cgggagccca gggctctccg gagagaacct cttaattgag

38221 cagagattgt tgcgatccgt acggaaagta aaaaaaaatt tatcagagaa gattgttagt

38281 tcttcacatt tctgtggatt ctctagtaga gtacctaaaa atatagtaag ctacagaatc

38341 tttttgcttc tttttgttat cttcaaaatt accgtaagaa tagttacaac ggtgttattt

38401 atttctggaa catttttgat ttcaaaatga ttattctaac ataaaagtcc ataatgaaat

38461 tatagcattt ccgagatgtt ttcttattta agtatcatgg tttttagatt tactattgtg

38521 tctatcttgt cgggttgtta tgaggtttcg taaaaaatga accgaacttg aatttaatcg

38581 aagtagcggg aacctagctg atagtaatcg tacgtcattc gaaatgtaat aatgcacggt

38641 tgggtgtatc gattgtttca aggaacgcat agcttgagta ttagatggtt tcgacgaaca

38701 tcaaacatcc aaaaaagaaa aaacacttaa aataaagaac taaagggcga agcttgtaag

38761 cgcgtgcttg cacccaagca gtactccctc cgggttttat taagtgcagc atgacgaaac

38821 attaaaaaag acttccttcc agctaaccga gcgaaactta gccgagtact aagtaagtcc

38881 ttcggtcctg ttttttagac ttagctgaac cataatcagt tctgtagaac cgtttcccgg

38941 aggaggaggc taacgtcttt ggagaaacga aggatgttgc agagagatgg cacccttccc

39001 tggttcaatt tgattggcaa ttatcttatg taaaggaagt acattgttta caggccataa

39061 ataatgttct atttacttaa gttatttcaa tctgattata gaagtataaa tcatcgtaga

39121 taaaaaattc ccatagatga tctaaatcca tgtaaaacac gacagaaatt aatgatcata

39181 aaggatcaaa tacatttacc tcaccatcac ccaaaaacaa aaaatattta ttatattcga

39241 tatattggta ttttcctgct aattatcttt gtatgtggtt tttctattgt ggaggagcct

39301 ataagcattc tttagcgtat gactctggca atattacagt agaagcttat atggcttctg

39361 ttatcgcctt aataaagccc taaatacaga aaaaaaaaaa ccggataaaa ggatactaga

39421 aataactcat acaatgaaga ccccaacaat agatacttga ttatgatggg attgtctact

39481 ctataatata taagtaacta gaatatgaaa cagaatcacc ctgaaaacaa agctggccga

39541 acgatgggtt ggacttgaaa cacctaagga cgcagttcct atgaatcatc atatatagca

39601 atatagcaga aaaagccatt aaaacgaaca acaattatta gggcggtata acgcagctct

39661 gtagcgctgg atcgtcgcct atgtatgcta caaacgatta ggctactggt ccgctctaaa

39721 gttgtaaaat gtcataagaa ttctgaacgt gacgccatac acgcaaaaaa cacacccact

39781 aagtaacttt ccattctaga agaaccgcaa taccataata tttcctaatc gagacagggg

39841 aggatacaaa gtaatggccc gcgtaggtag tttcaaccta ctttttctac ttacttatcg

39901 aaaccgaaga gcgtagcagg gcgaaacatg cgggcttcaa tttcatcgtc agaaaacgca

39961 taaatgctct tttcgcggaa ataaactaaa atatggagct cggggctgag ccttgtgtct

40021 tctagttaaa agtccgaaaa agcgagaagg tccaattagt atggctgaag atacgaggtt

40081 cgaaaaaaaa atatttctct gttcgaaaat ctattgtgtg ccctaacaaa atgattgtat

40141 agtacgacgc aaggttgacc tcacctccac tatttgtgct atgcggttta ttattcagaa

40201 aatagaaacc atgttgacaa acatagcatt tgtttgatat agaaaaaaaa cccggcatca

40261 cttacttcaa tttaaggtat gtcaaaaaga aagaagagaa aaaactaaaa aaaaaagaat

40321 aataatgatc ctatgctaat aaaacaaatt gttatacgga aaaaagccca agagattgaa

40381 aaaaaatctc catatattct tttatttcat tgtagtggct tgacaagtcg acaatggcga

40441 caactcaaaa atctattgtg tgcctttcga ggtagaactt tatttcaacc aaattacaaa

40501 cacaaatgtc cacagaaaaa caagcaagat ggtaaacaag gtgtttttat tgagcagctt

40561 gcttatagcg caggtcccac ttgtatcttg tacttaacta aagaagcacc agataataca

40621 tggtcacagt tattacaacc aaaatacaac caaaatctag ttttattata cggacaactt

40681 caatctactt tagtcaatca tatggatata aaaaaagcgg ctaatttaga aataacacca

40741 gtatttcaac aactttttga gctcattttt tacccatata attctttatg tttttgtttg

40801 aataagccaa ttcatgcttc acccacgaga cgcaaggagg gcgaagacgc aaggaaggtt

40861 acttagtcac cagaggataa ccacttagta acccccactt tgccaattaa attaaggctt

40921 tattaacaga actggggcgc gtagtttata gcgaagcttt ttttttttcc aatattttgg

40981 gaaatcctcc aaaaatattt tttgctgcga aaagcagcgc cctcaaagat tgtaaagatt

41041 taagctaatg gatgatttgt ttttttggcg aataacggga tttgaacccg tgttttcaaa

41101 ttcacaatct gacactttca cctattaagt tatactcgcc cttttttcta attcaaacat

41161 taaaatactc gctatcggat tcgaaccgat aacccatagg aacagatttt gagactgccg

41221 tgtttaccat tttcaccaag cgagtatgct cactatcgga cttgaaccga taacccatag

41281 gaacagattt taagtctgtc gtgtttacca ttttcaccaa gtgagcttag ggaagcgaag

41341 cgcagaatgg aaacacaatc tttttgtttc gttttatctt tgccatttca ttttatagac

41401 atctccggct tattccggct cggctgaatc tgcggcattt tctcaaatat ttgaaaaatc

41461 tatagtcctc tgtcttaagg tgtgtagaga ctccttttta ttgatttata ccagtctatc

41521 ttagatccga gatagtgccg aaaaaatgat tcaatttaat ctaaccatgg ttgccttggc

41581 aatggcaatg acttttatac aatatgcaga atactccaaa acctctattt agaactaaaa

41641 gaagataatg cttttttata aaaaaatcat aaagtgcaaa ggttaaaatt ttgagctttg

41701 ttttacatag ttttgccatc tatataatag aatagaataa gaaataataa gttggcgagg

41761 aagtaaaaat atatatatat ctatctatat atatatatat ttttttttat tctgtggtct

41821 tgcaattgtg ctcaaagtta agggccccag ccccctattt cttatcgttt tcgtcaatca

41881 aataaaggct tcgtttagcc aggggttatt cttctcctct gctgaattgc ttcgcgaaca

41941 aagtaggcga aaaaaataaa gatttttttt gtccgcctta ttctcttcca ttatttaaat

42001 ggccctatga gcttttacca agcaaaatat tgattgcata agccatcggc ttctacagag

42061 accaataaag tgataacagc agcgtagcca agcttgttcc gcccgctgtg tgcaaaaaaa

42121 gagttttttt ccggcactta gtaaaacaaa attttgacac tccttgcgtt gcggatgatg

42181 agtgaattac taataaaatg gagtgatcca agatgacctc tctttcaatt tcaattatgt

42241 cattatatag taatggcatg cggcgaactc catcggatgc gccaaaaact tgatttgttg

42301 ggctgtgtca atttattaag attgacagag ccttcatcta agtcttggct cgtgcagctg

42361 tcgcccaaaa tacaatcaat tatctaccca atcgactgta ttttggtcac aataaaaaaa

42421 aatgatttat gtatgtattt attaatcgta actttacctt tactaggtag ttgtgtagca

42481 ggtgcttttg gtcgttttct tggttcacga ggaactgcta tagtcacaac cacgtgtgtt

42541 tcattatctt ttattttatc tttgattgtt ttttatgaag ttgcactggg agccagtgct

42601 tgctatataa aaattgctcc atggattttt tccgagatgt ttgatgcttc ttggggcttc

42661 tttggcgacc gtgaagtcac cggatgaatt gctggataga tagattatct ggacgctgca

42721 gttgctttgc ggtgagacgg actcgactca ctctatgggg cgaacttctt tagagcatca

42781 tagcatgtcg ggaaaagggc atactggacg tagccaaaaa tatccttggc tgtgccctga

42841 ccgtgtcttt gagaaatagg tgaggccgta ggtcacgaac gtaaggtgga ggaagtatcc

42901 caaacttagc gcatcgggcg aggcccgcgt tccccctgca tataggcagc aagagggcgc

42961 atatgcctga acaaataggg ggcctgagtc caataagggc agcacaccac ttcaagcgca

43021 cctatgtctc gatatcgata gagtattcgg tggaatcggt gaaccataca tttttctaga

43081 tagagatgcg tgggacagag ggcttgtagt acctgcctac tcgctttaaa tattcaaaaa

43141 tttttttgct gcgagttctt tcaggaaaac gctgatatta gcaaaaggga agaggcctaa

43201 gtcggcagat gccgtacgct tgagtggcaa aggtaagcga cactatacga ctaagcaatg

43261 aaaaaaaaag tatgcagcga ataaaaaaaa atctattttt tgctgcggcc cgcttaaaca

43321 tacagtggtt actccaagcc ttaataacaa tctcaagttg gtgagccgtg tgataggtaa

43381 ctatctcgca cggttcgggg agcactttat tattttactt gagtgaacgg ccgccgcatt

43441 gcggtacgca aaaaatttcc tgcttgattc tgcgattcaa ggctccagta aaataaaact

43501 tttgactctg tgtttgatag tccgactgta gttatgttaa ttgtggttac atttgtaagt

43561 agcttagttc atctttattc catttcatat atgtctgagg atccacacag ccctcgattt

43621 atgtgctatt tatccatttt tacttttttt atgctaatgt tggtcaccgg agataacttt

43681 attcaattat ttttaggatg ggaaggagta ggtctcgctt catatttgtt aattaatttc

43741 tggtttacac gacttcaggc caataaagca gctataaaag ctatgcttgt caatcgagta

43801 ggtgatttcg gattagctct ggggattatg ggttgtttta ctattttcca aacagtagac

43861 ttttcgacta tttttgctcg tgccggcgcc ttttccgaac tccatcatta tttcattttc

43921 tgcaatatga gatttcatgc cataactgtt atttgtattt tacttttcat tggcgctgtt

43981 ggaaaatctg cacaaatagg attgcatact cggttacctg atgcaatgga gggttttcga

44041 atatttgagg gctctcatgt gccaataggt acattataac aatctcactg tatgctggaa

44101 tcgtcgacca aatgagagtc cccatcggaa aaatatctca ttttgaccaa tcagcaggaa

44161 acctataaag gaaggccgag cccacccaac gaagtaaagg ctgaaggagc tctataggat

44221 cctcagagac tgtacgtgag acctcttgtc cgaaatggaa tttatccttg aaaagagaag

44281 ctggccagat ttaactaaga tacaaagcat tcttttgtcg tgaagattcg atcatctttt

44341 ttttagatta tatagtctag atatgcaggt cttatggaat agaaggatcc gtatagggtt

44401 tgggcatgta gaaaaccttg tattgaatca aatactcctc atcattttgg ttataagctg

44461 gaaacggaaa aaaatatata tattgttttt ttctagcgcg gcctgatgtc acattccaac

44521 tttccgctcg agcccagcct tattgtcatc ctcaccaaag cgccgcgcgc tgaatctacc

44581 aggatgcagt ttaaaaaagc ttaaatattt tttgttgctt cgcaaaatat atatatactt

44641 gcgaagagcg ttgggataga gctaagaagg tatctcatgt ataaaagaaa aaaaacatct

44701 tagttgttag ggacgcagca tccgtaagat tcttgggaga gtctctattt tataagtgga

44761 agatacagtc cctacttttg ctaagctcat cagcttatta cctaatgctc taaaatatta

44821 tttctttgcc ttatgggagc gtaagagatt attaaagagt agcccactcc agtatctgct

44881 ttgattcacg cagctactat ggtaacagca ggcgttttta tgatagcaag gtgctcccct

44941 ttattcgaat attcacccac tgctttgatt gttattactt ttgtaggggc tatgacgtca

45001 ttcttcgcgg caaccactgg aatattacag aatgatttaa agagggtcat agcctattca

45061 acttgtagcc aattaggcta tatgatattt gcttgcggta tttccaacta ttccgttagc

45121 gtatttcatt taatgaatca cgcttttttt aaagcattac tttttttgag tgcaggttca

45181 gtgattcatg ccatgtcgga tgagcaagat atgcgtaaga tgggagggct tgcttccttg

45241 ttacctttca cttatgctat gatgcttata ggcagcttat ctttaatagg ttttcctttt

45301 tgtactggat tttattctaa agatgttatt ttagagctcg cttatactaa atatacgatt

45361 agtggtaact ttgctttctg gttaggaagt gtttctgtct tctttacttc ttattattct

45421 tttcgtttac tttttttaac ttttttagca tcaacaaatt cattcaagcg agacatctta

45481 cgatgtcatg atgcgcccat tcttatggca atccctttaa tctttttagc ttttggaagt

45541 atttttgtag gatacgtggc caaagtgtga cccgttagcc tataagtcac tcctgtgacg

45601 aagcggctgt tgctcactca aaaaataaat tatttttcaa agtggacaat aattaagaat

45661 tggatgcggg cgaaattccc gccaatggct gagatgttca gtcgactctc ctccctttgt

45721 aggaggtccg gcagcctccg agttagaggt aagcaaaaaa aggaggagga aagaggcctt

45781 ggtgaaccac cataagtaaa caagtgtaag ctttgttgcc cgacagtatc aagtactgac

45841 cacactgagg gacgaaccct aaagttaaag ggaggaatga agggtacttg cagtgcaaat

45901 ttttggtctt gcaccgaaca tccaatggac cttggactaa atggccactg tctgaaagga

45961 ctatgtccaa gggaccaccc cgcaaagtac atcttgcgcc tatgggccgc tataactatc

46021 caataaactc aaaactggaa aactctggta gggctccctt gcggcgggct gccaagctat

46081 aaacccgacg agagcaggat tctctaaaaa gccaaggccg cagagtgcag ccagccaaaa

46141 tatatttctt ttcgcagcat tttcattatg ctcacttgga gatttaggat ttcagtaaaa

46201 actggaaagg agaataagtt atgagtaggt tctacataga tacccaaacg ataccctggg

46261 aacaaattcc ttggcccaag gtcgaggcag ttgtttttag actccaaacc agaatttacc

46321 aagcttctcg ctccgacaat atggacaaga tgcgtgctct acaatttaga ctcatacgaa

46381 atctacatgc cagactctta gccgtaagac aagttacaca ggagaaccaa aggaaaaagg

46441 accctgacat ttacaagaag ttggttgcct cgggatcaca aaaaatagag atggcaagag

46501 gtcttcaatt ggatggaaag gctacaccca ttcgtcagat aataatatca aagccccaga

46561 aagaaaaaga gcaccccaga aaactacgag caaaaaaaaa atatagattt tgttgcgcct

46621 tttctgcgct aggcgcaaga aaacgtagag tcaatcaggc acgctttacg cctggaaaat

46681 ggaaagcaaa aaacaatcta tatttttttt ttcgaactaa actttgcgtc tttttatctt

46741 tttggcctat ttgtgttatc gaagacagag cgaagcaggc tttaatcaaa atggccttag

46801 aaactgaatg ggaagcccgc ttcgaaccca attcttatgg attcagaccc ggacgaagct

46861 gccaggatgc catcaaagca atattcttag ctattcaagc caagcccaag tatgttttga

46921 acgcggacat ttccaaatgt ttcgatcgca tcaaccacca agccctcttg gacaaactga

46981 aaactttgcc taatttagcc aaacaggtca aagcctggct taaagtcaac ctcatgaccg

47041 cattgggaaa taatgctcag tacatggaaa ggggcaccta tcccgtgatc ttcccactgc

47101 tagccaacat tgctctccat gggatggaga cagctttaac agagtggtca ctgagagcat

47161 atcccaaaga accaactccg atactggtta gatatgccaa cgacttcgtt gtacttcacc

47221 aatccaagga gatcatagaa caagctcagg cattcctgag agaatggtta aagtgcatgg

47281 gactagaatt gcactcagat aaaatccaga tagtcaacac cggatccggg ttccaattta

47341 tagggttttc gatcaatcat atttggaaat ggggcaaagt tagaacttta ataactccgt

47401 ctcgtgagtc tcgccggaga tttcttgaag atattcgacg ggccattcga ttgtcaaaag

47461 gaaaagcagc ctaccaactt atcataaccc tggtacccca gatagtagga tggggcaact

47521 acttcaaata ctctgaatgc aacaatatat ttcggagatt agaccatgat atctttcaaa

47581 agatccgagc atgggtatac cgacggcatc caacatgggg tcgtgataaa atcaaacaaa

47641 agtacttccc cgaaaagaga agctggctct tcaaagggaa agtataccaa gataactgga

47701 ttttgtacga ctcttgcaca acggcctttg gagtgaaaag agaatattac ctggtcaaac

47761 tgagttggat agctagccac aagcacatta aggcaagaca aaataagagc ccaataaaat

47821 gaaaagccgg gtgaaaagag agcgcaagag caaaaaaatc tatatttttt ttttgctctt

47881 gacttctctg ttctactgcg cacctttaac ggaactacta tctactgaaa tctaaaggtt

47941 ccaaatgcga taaccaaaaa gctgaagggc tctttcttta ttatatcgcc caaacatgtt

48001 agaaaaccgc gagagcataa aagtagagcg catcaaagtg aaatccttgt gttttcgtag

48061 aaaacttatg gacaagcact gccatattgg aaacaagcaa gaaaacgtaa aaattctgag

48121 aggagccgta tgaggcggaa gcctcacgta cggttttgaa gccaagccgt tccatcgctg

48181 gaacggctta ggaaccgata tgatgattgg tttaggtacc aatttttggg ctaattccct

48241 tttcatacta ccaaaaaatg aaattcttgc cgaatccgag tttgctactc caacaattat

48301 caaactaatt cctattttgt ttagtacttt aggtgctttt atggcatata atataaattt

48361 tgtagcaaat ccattcattt ttgctttgaa aactagtcct ttgggtaatc gattatattg

48421 ctttttaaat aagcgctggt tttttgataa actttttaat gactttctag ttaggttctt

48481 tttgcgtttt ggatatgaag tttcatttaa agttttagac aagggtgcta ttgaaatctt

48541 gggaccttat ggaatttcgt acacatttcg aaaattagcc aagcagataa gtaaacttca

48601 aagtggtttt gtttatcatt atgcttttgt aatgttgatt ggcctaacca tatttattac

48661 cataataggt ctgtgggatt ttatttcttt ttgggtagat aatcgattgt attttattta

48721 catagtgagt tttctattta ttcattttga aaacgacatt agcacgaact aaaggggcat

48781 acttccttat ataataccat gaaactttaa gggacgcaat gataagccag tgctacgccc

48841 ttttttcggc ttcgttgaga gggagggctg aggcccgacg aagcctaaca agcaaaaaaa

48901 atagattttt ggagcctaag ctatgctctg cggtctagct acgataaatc atgaaaaaaa

48961 aatggatcct cgaataaagc acgtaattgc tttgggttta cgaggaatct gaaaaagaag

49021 aagaaaagag taaaagttgg aatgatagaa taatggatca aagaaaggaa aatgaaaaaa

49081 atcataaaac gaaaaaaaaa attttcggct ttttttatcc tctccgaccg tgactggggc

49141 cgtccggtca taatagcaag ccctagagca ttggacaagg cggcaaaagg cgaagcatgc

49201 aattacatag tatgcgcgta taaaagctca tcaagttatg caattattgc gggctttatg

49261 tatataagtg cagggggtta tgatgatata cccataaggc ctctatggct agaaagccga

49321 gaaggaatgt ggacccgcgg cctgcgaaga gagctgcgtc cccgggatct tttggaaaaa

49381 gagtaaacat ttatgttaca atttttagct ccattttatt ccaatctgag tggtcttatt

49441 ctgtgtcctt tgttaggaag cattattctt tttgttatcc ctgatcccag aatacgactg

49501 atacgaagta ttggtttgtg cacctctttg attacttttc tatattccct tctttttcgg

49561 atacaattcg ataattctac agccaaattt caattcgtgg aaaccattcg atggcttcct

49621 tattcaaaca tcaattttta tataggtata gatggtatct ctttattttt cgtggttttg

49681 accacatttt taattcctat tcgcattcta gtaggttggt ccagtattaa aagttataaa

49741 aaagagtata tgatagcatt tctaatttgt gaatctttca tgattgctgt gtttagcatg

49801 ctggatcttt tactatttta tgtttttttc gaaagcgttt taatccctat gttgtgcgga

49861 gctgagcatc tgatattcgc ggcgcgaagc gccgcctctg caggagcctt gtgcagtaaa

49921 cccttccgag cgatctgaag accggtaggt cccgcgaagc tttcggagaa gggtagtctt

49981 gtgtgtaagc atagcttttt ggtcgaaccc gtcggatgac ttatttggga ttgaggggta

50041 ctttgagtgg gtactttgca ggacttcact ctgcctactc gaatattgta taaacatgca

50101 ggaaagggtg ctcctaggca gggaagaatg gagttttgct acgagaaaac agttttcgtg

50161 aagtctaggg ggtgcagaca cacttgcgcg aattacagat gacggctaca agggtggtgg

50221 ggaaaagaaa agtacccgac gcctggggat ggacataaat ttgttaacta cctattgagc

50281 tgacaaggat aatcgtcctg atcttgaaag aggacctcag gtcaattcct ctgtcaagaa

50341 gttattggcg aggccgcggg atgagtcgct ggaacaaaaa gggagaccga aataaaaaaa

50401 tattttagaa ctacaagccg aaaaaaggcg taaagccctt tctgcaaaaa tctatatatt

50461 tttttttttg cttggctttt attttcggct catccgctat tttgagaagg agccgtatga

50521 cgcgagagtg tcatgtacgg ttctttgaga agggtgtggg tacctacagg agctttttct

50581 cgacccattt atcatgggga gataaagccg agggcctatc atcccttact ctcctattat

50641 cataggggta tggggttcta gacaaagaaa aatccaagca gcatatcagt ttttcttata

50701 taccttactt ggatctgtct tcatgctctt agccatttta tttatttttt tccaaacagg

50761 aaccactgat ttacaaatat tattaaccac agaatttagt gagcggcgcc aaatattgct

50821 atggattgct ttttttgctc cttttcccgt aaaagtgcct atggtaccag ttcatatttg

50881 gttacctgaa gctcacgtag aggcacctac ggctggatct gtaatcttgg caggaattct

50941 tttaaaatta ggaacctatg gttttttaag attttctata cccatgtttc ctgaagcgac

51001 actttatttt actcctttca ttcatacttt aagcgttatt gctattatat atacttcctt

51061 gactacaata agacaaatcg atctgaagaa aattattgcc tactcttcag tagctcatat

51121 gaattttgtg actattggta tgttcagtct aaacatacaa ggaattgaag gtagtatttt

51181 acttatgtta agtcatggaa tggtttcttc agcccttttt ctatgtgttg gtgttttata

51241 tgaccgacat aagactcgac ttgttaaata ttatggaggt ttagtcagca ccatgccaat

51301 gttctctacg atttttttat tctttaccct agccaatatg agtttacccg gtactagcag

51361 ctttatcgga gaatttctta ttttagtagg agctttccaa agaaatagct tagtggccac

51421 attagcggca cttggaatga ttttaggcgc agcttattct ctttggctat ataatcgtgt

51481 gatttttggg aatttcaaac ccaaattcct ccaaaaattc tccgatttaa atagaagaga

51541 agttctaata tttttccctt ttattgtcgg ggttatttgg atgggtgttt accccgaagt

51601 tttcttagag tgtatgcata cttccgtaag taacttagtg caacatggaa aatttgatta

51661 aaaaacataa aaaagaggtt tgaagaaatg tttgaacatg attttttagc gcttttccca

51721 gagatctttc ttattaacgc aaccatcatt ttgcttattt atggagttgt atttagtacc

51781 tctagaaaat atgattatcc accattagtg tgtaatgtta gttggcttgg tttacttagt

51841 gttgtgcgcc taggagggcg cgtttgaaaa gacgatagtt gaaaacaatc gctcgggtag

51901 actccctaac cttatgtggg atcagaccgc agggaaccat agcatgggta ctatccgcgt

51961 ttcgtcgcaa gtacaattgt acgcatagga gtaaggtaac ttcccccaac gaaaggcggg

52021 gccggaaggc acgtgggctc gaacgctact cacgtgcgag caaccctccg gacgtgactg

52081 taatggacag aaaaagtggt acacgtaact aaacgacaag caaacggcca aacgcgcaaa

52141 ctagggatca tccaaatgga ctctatagga accggctttg ataaaagcag ggcgtagcaa

52201 atttgctacg attttcaaaa aaaaaatact tttgaaaatc ccttggagac caaggcttta

52261 gccaaaatgt acgggtgaca gatccttcca taatgtaccc tgagaaatac accgggcaat

52321 taatgttgag catacgacga tgccctttag agtgaaagcc tcggaggaaa aggaggtagg

52381 tgataatgcg ctgtactttt cagacgcggc gcgcgccgcg tctgaaaagt acagcaaact

52441 cggagaagca atttaggata atgtcattaa agagaaaaaa aaatatcttt ttcgctgagt

52501 gctactactt ttagccccat attcatgaga cttcctacgt caatatacaa ccctaaataa

52561 aagactaggg caatagctag gtaaaacagc gaatttgatt aatctaccta caaacgtaac

52621 caataaaaga atggaagaca atgtggcata acgccaactc cgaaatgatc agtgttttat

52681 tttgttcatg caggcccggc cttagagggt ttcggtccgt aaacctgaaa aagttatcat

52741 ggacgagcca catgcgggga aactcgcacg tgtggttctg accggggggg gggggaaaag

52801 caccctatcg gtatctaata actatcttat tggtcgcttc tagcacacct ctaactgttg

52861 ccaatttatt ctataataat ttaataatag acaattttac atatttttgc caaatctttc

52921 tattaataag tacggctagc actatagtta tgtgtctggg ttatttcaaa gaagagagtt

52981 tgaatgcttt tgaatctatt gtcctaattc tactttctac ttgcagtatg ctcttcatga

53041 tttcggctta tgatttaatt gccatgtatt tagctattga gcttcaaagt ttatgttttt

53101 atgtgatcgc agcatcaaaa agagactctg aattttctac agaagctggc ttaaaatatt

53161 ttattttagg tgcattctcc tctggaattt tattgtttgg ttgttctatg atttatggat

53221 ttactggagt taccaacttt gaggaattag ctaagatttt cactggatat gaaatcactt

53281 tatttggtgc tcaatctagt ggtatcttta tggggattct atttattgct gtaggttttt

53341 tattcaagat cacagcagtt ccttttcata tgtgggcacc tgatgtatac gagggttcac

53401 ctactctggt tacagcattc ttttctattg cacccaaaat atctattctt gccaatatgg

53461 tacgtgtttt tatttatagt ttttatgatc caacatggca acaactattc tttttttgca

53521 gcattgcttc tatgatctta ggagcattgg ctgcaatggc tcaaaacaaa gtcaaaagac

53581 ttttagctta tagttctatt ggacatgtag gttatctttt tattggtttt tcatgtggaa

53641 ccatagaagg gattcaatcg ctactaattg gtgttttcat ttatgtatta atgaccatca

53701 atgtattcgc catagttttg gcattacgtc aaaaccgttt caaatatata gcagatttag

53761 gtgctttagc caaaactaat cctattttag ctattactct gtctattact atgttttcat

53821 acgcaggaat acccccgtta gccggatttt gtagcaaatt ctatttgttt tttgctgctc

53881 taggctgtgg ggcttactta ctagccttaa taggagttgt tactagtgtt atcagttgtt

53941 tttattatat acgctttgtg aaaataatgt attttgatac acctaaaaaa tggattctat

54001 ataaaccaat ggatcgtgaa aaatccttac tactagcaat tactttattt ttgatcagtt

54061 ttttcttttt atacccttct cctctattct tagttagcca tcaaatggca ctaagtctat

54121 gtttgtaagt tgtatgttta ataaataaat aaaattttta taggttcagc ataatataat

54181 aattgcataa ttcacaagtc tgaattggat tcaaggctga attcgagcaa ggcaacagag

54241 cgtagctttt cgcggggcgc gataaaaaaa aagaattttt ttcgcagatt agcctttgct

54301 aaaaacaagg aaagcactgt gcctccaatg actctccgta ccgttttctt tctccatcct

54361 cccggttgtt tccagcccca tcatttgtta tgcgaataat gtgggcacag caccggtttc

54421 attgcgtttc gtgaaagcag agaaatggcc acgcttcgca cctgcgccct gaatcatgac

54481 aaatgatttc tattcgcttc gcgcatagaa atagaggcct gcttttgttg gctactctct

54541 tctggtacca aaaatagatt taacaaaaaa aaaatagatt tttcatagat ttttggagaa

54601 tgaaactgag gaaagaaaaa cccaagcgga aaaagggact tgaaccctca acctcagcct

54661 tggcaaggct atactctacc attaagctat ttccgccagc ttcaacaaaa caaaacaata

54721 aggaaaaaat aagaaggcct ttacacttat cagatgtatt ctcttagtaa aatttgatgg

54781 ataggcccat gcttggaaag attcgaactc tcaaccccca aatccgtagt ttggtgctct

54841 atccgattga gctacaagca caaatttatt attcttaagc actacgtgtc atgtaggctg

54901 cattactata aagaaaaaac atatatatat acgaaaaaat attttaagaa ttgaaaaaaa

54961 aagagatatg ttttttttta ccttattcat ttaagcgatg gtatactttg taaattagga

55021 tagtattacc ctattgcatt atttcaaggc gtttatgcct tctgtgactc aagaaagttt

55081 attctagcat tgtggtcaaa ttagtcaaca ttttgaccgc agttaagtga tctggatgca

55141 ttataacacg ttagtaatca agttcaaaaa agaatacttt gttgatttga ttagctcgcg

55201 tttaggtttt actgctaaaa aagaaaaaat atatatagat tttgttttct catttctctt

55261 aatctattgg ctttatccaa aaagcggaaa tgcagacgtt attaaaggtc gctcttggtg

55321 taatgttgac caggtacaga gttttttttt agttgaaagc gttatgaatt atcgtaggta

55381 gatgaagaaa aaaagtggcg cataaacggg gcacaggccg caaattgtgc cacttttttt

55441 tctctttatt gcagcgcagc tctggctgac catttttctt tgaaataatt ttgtcccttt

55501 cgtctagggg tataggacat cgtcttttca tggcgagaac acgggttcga atcccgtaag

55561 ggatagcctt acttacatat gctccgagag ccaagcgaaa tgagctttct agtgcacgta

55621 ggaattttct gctcaaaaaa ggaaaggaca caaaaaattg aaaaaatact tttttttcag

55681 tgtcttcgcc ctttattata gtttttaact gttctctctt attcttcttt ctcgtgcctt

55741 catgataaaa aaaaatcgta gaaaacatag taatgaaagg gcgcggggta tagcggccaa

55801 aggccccatt cccataacgg atagagcata agaaataaga aagaaaaatt ttatgcaact

55861 ttctaaaaaa aaccaagtaa gtgaaatatg cctacaataa atcaattgat tcgtcatggt

55921 agaaagtcga aacggcgcac acaacgtact cgagctttaa ctcaatgtcc tcaaaagcaa

55981 ggagtatgcc tgcgtgtttc gacgagaaca ccaaagaaac ctaattcggc tttacgcaag

56041 atagccaaag tacgtttaac caatcgaaat gagataattg cttacattcc cggcgaaggc

56101 cataatttgc aagagcattc tgtggttatg gttagagggg gtagagcgaa agatttgcca

56161 ggtgtaaaat accattgtat tcgaggaatt aaagatttgc agggaatacc gagtcgacgt

56221 cgaggcagat ctaaatatgg tacaaaaaaa cccaaagatt ctatatgaat ttatttgtca

56281 aatcatcgaa tttcagcttt gtgtttggtt tattctttga ttggtttcac caatcaaaac

56341 tttcagaaaa ggcaggaatg gaaaaaaagg aaaattggcc atttagcgga aaaaatctat

56401 tttttttttc agaaagcttt tttgcgcgtc gtttatcgca ttgtagatat ttatgctatg

56461 cccttccagg gcatgtgcca tcaagaccta aaggtcgtga ggcaagcata tacaatagct

56521 cagacaattt gggctatatc cggggcttgc atggtaaaca aaaacaatta ataaaaaaat

56581 tggtccatat ttgcatgatt gatggtaaaa aaacgagatc tcgtgctatt gtttataaaa

56641 cttttcattg tctagctcag catggcgata tattaagact tttggttaat gccatagaaa

56701 atgtcaagcc tgtttgtgaa gtgaaaaagg taagaatttc tggtactact caattagtac

56761 catctattat agcaacaaat cgtcaagaaa ccttagccat tcgttggatg ctcgaagcgg

56821 cagccaaacg acgtattaat aaaaaaagca tgagcttaga tcaatgttta gctgatgaga

56881 tattggatgc ttcccgaaag atggggattg cacgtaaaaa aagggatgat cttcataaac

56941 tggctcaagc caatcgtagt ttttcgcatt atagatggtg gtaaactatt tgaagtggaa

57001 aaagaaagaa atcaggcgac gagggaggcg aagcgcatta tttaaaaact tttctgcgct

57061 tcgccccctt ttgctctgcc ccactcgggg attggagaaa gaaaaaaaag gccaagcttc

57121 gttatgcgct tggctactca tttataagga tctcatggct tttatcataa tgaaactatt

57181 cgtcctttct cagaattaga cattaagcgt ctcagctcga gataagtttg ccgcatcaaa

57241 gaagggttgc aagagagtgg gcgcagcaaa tatatatttt ttttgcttgc tgttttttct

57301 atcaaaaaat cgaccagaaa gctagtaata ttcgcgtagt ttttttttgt gcaccctgca

57361 ggtagcacac aattatcagc acaccggggc gaccaaagac aacttttgtc aagctgcggg

57421 ggggggggga gtacctgcgg cctatttgtt ttggtgggaa ttagtccccg gggtcccagg

57481 atattcgctt ccgccaacag ggaactctac aaggccgcag ggcgcagcaa aatatatata

57541 tatatataat attttttttt tcgtagcttt ttgcatcccg cgcgttcaaa ggtcttcgac

57601 cattggtgtg cattattttg cgtcatcaaa agcaaatcca gcttttttat tatggttgat

57661 gatgacgcgc ttaaaaagta aagaagtgat ttttgatgct ccttacacca gtcttttaat

57721 gaaggtttat agcatcattt aaataaatac aaattaaaat agtaaaaaca taagcttgta

57781 atatagcaac acctaattcc aaaccagtta atgcgaatac tattagaaaa ggagcaagat

57841 gtgctaaata cataataccc cccatagata gcatagtcca agcaaacccg cttagaatct

57901 ttactaaact atgaccagcc atcatattgg cgaataaacg tattcctaga cttaatgcgc

57961 gaaaacgata ggagattagc tcgagaagta ctaggaaggg tgctaacggc aagggtactc

58021 cttgcggcaa taaaatactg aaaaaatgaa gcccatgtgt ttgaaatcca actatagtga

58081 ttccaataaa aagagataat gaaagaccca gggtaattat aaaatgactt gttactgtaa

58141 aactataggg tatcatacca atgagattac taaatgataa aaaagtgaaa gtgacgaaaa

58201 ttagaggaaa aaagcgttgt tttatcgaag aaggaccgct tatttgttca tttaccaagt

58261 taagcacaaa atcataaata atttcaacaa aggattgcca agcatttggt actaagtgtc

58321 ctccatttaa agtaacgaaa tgaactagaa gcaatactag actgatagtt aatagcataa

58381 acaaagatga attgggtcgg taggggaaaa aaagattttt ttttttgctc catttgaacc

58441 ttacttaggc ccagggttta aagccgttcc cctcctatag aaccgtacgt gatagtctcc

58501 catcatacgg ctcctctctc ttcgggaccg gccaaaggtc caatagaggc tttattttgg

58561 cagccatctt caaaaaagta ttttttttta cttagccgga gagagccagg actacattcc

58621 tcgccgctta ttttgtggga ggttttccat ccatcctcga gcgcattcca cagtatcctg

58681 ggcactcgcc ctcatagccg tcaccccagt tgatctaccc gcgcgttctg tctaggattc

58741 tctaggctcg accggcgtgt gccggcgtga acatggtttg tatcctgacc caggggcttc

58801 ctttcactgt ttaccatcgg ctatttgagt agatcagtcc tcatattcgt ctcccttcca

58861 aaagagcggc cattctcgag attcgtaaac ctatcctgta cagaacactt tcctgactcc

58921 acggcatcga agtaaacggg agttggatta tcgtctaaaa ccccgacgaa gtgtttacac

58981 catcgagtag tgggcgcgag ctccgcacgt aaatgaaaga tagaaatttc ctatatgaat

59041 aggaatcaat ggaataattg caaattgttc tagcggactg caagccatga tgaattctct

59101 tttttttatt ttagcgcgag gcgaaactaa gaagccgctt cgttgcttga cgcttttcaa

59161 ggcagagtct tgagagaaaa taaaaaagta tttttatttc tttatttttc tttctttcgg

59221 tattttttca tatatatata ttatatatga agaaatacct cgaagccaag ccggatttgc

59281 cctacattcg cgcagcgaat agagcgttaa tttctattta tgtttttctt ttcttaaata

59341 atgaatggta actttttaga aagaaaggag agcgaagcat aatcaaatgg atttgaagag

59401 ctaaaaaaaa atcttttttt ttacttttct gcattttaga atatatatga aaaaaaatct

59461 atatattttt gtgcgcctag atttttcgtt ggtttgctgc gcgtttttct tctataagct

59521 aatggacaaa gtatgcgtga tttcgtgcca tctatgttcg ttttagaaga gaataaaact

59581 caaagtttct aagcctctcc ttgccccaat tccataagtt ggggtcgaag accccaacca

59641 tgtgctttcc aatatttaat caagaagcaa aagtgaaaag cgctcattta catagctaat

59701 ttatgaatcg tttcgtacgg aaacaactag aagcggtttc agctctggaa gccttcggtg

59761 atgcaaggtt caagagtgtc taagggcaca aggaataaag tttataaatg aagatggtta

59821 ttaattatca tacatgatga atttgcttta tgcgaatcca aaatcgaaaa tagtctacgg

59881 atttcacgag cgaaagatag ttttgtacgc aaagttcgcc atgcatttac tattatgata

59941 tattgagatt tatctactcg actgacgaga acctgagaca ctttttattt atgatgtcgt

60001 tccacgaagc cttctaatga gctatatcca aagcgggagg aggaaggcga ataagaaaaa

60061 aaatacatat tttttttgct tcctgttgca ctttttgacc gaaggcttct ttcgtatcga

60121 gagcgctctt atttcgcact tcttcttccg ctccagcagg atctctttct tatggggctc

60181 tttttttatg ctgtgtacat gtgttggctt tagttgtttc tttgcctggt ttgtgtttgc

60241 tcgcattatc tgttctccag atgatgcgta gaaaaaaaat atatatattt tttttcattg

60301 ttagagcaaa tgataaaagg gacttagtaa aataaccatg atactttttt ctgttttttc

60361 gagcattgct ttagtctcta gtgttatggt aatacgtgct aaaaatccag tccattctgt

60421 tttatttttc atcttagttt ttttcaacac ttcgggttta cttgttttgt taggtcttga

60481 cttctttgct atgatttttt tagtggttta tgtaggagct attgccgttt tattttcatt

60541 tgtagctatg atgttaaata ttaaaatagc agaaattcac gagaatgtat tgcgttattt

60601 acctgtaggt ggtattattg gagttatttt tttgttggaa atttttttca ttgtagataa

60661 tgattacatt ccaatattac caacgaaatt gagtacaact tatttaacat atacagttta

60721 tgctgaaaag atacaaagtt ggactaattt ggaaacatta ggcaatttac tttataccac

60781 ctattttgtt ttgtttttgg tttctagtct tattttatta gtagctatga ttggagctat

60841 agtacttact atgcataaaa ctactcaagt caaaagacag gatgtgttcc ggcaaaatgc

60901 tatagatttt aaaaatacta tcaaaaaaat cagagatatt tgaaggcttc agctctctga

60961 agccattaag aagctcaaaa aaaaaggtat atatattttt ttttgtacgc ttcttttttt

61021 tcttgccgtg ccttttcaat ctttgctttt cttttgcaca aagcaaagaa agcgggtaaa

61081 cccccgtcct agctaaggtt ttgcgggact aaagtccttc taccaataat aaatatgggg

61141 cgaaaagaac aaaagctaaa tacaaaaata tatatagcag caaacacaaa gagtcaaaag

61201 tgttggagca gctaccattg cggcaagtag ttttctattt tgctatagca gcaaagagcg

61261 ttattcagcg ttattgagcg ttattgagcg ttattggtgg gtagttttct atgccatttt

61321 tatggcagct cgttttctct ctcaagttac tgcattgcta gtaggatgca tttatctttt

61381 ctattcaatc caaacccctt tactgcaact tttgttgcta ttcgtgttct cactcattcc

61441 gcagttagcg caaatgactg ttaagtgcgc tgaatatatt gatagctgaa tatattgctg

61501 aatatattga tagcggttag cgcaaatatt gatagctgaa tatattgata caggagaagg

61561 acccttctgc tgaatatatt gatacaggat ttcgcttttt acgcagcata ctatagatta

61621 tgtctaagtt aggtatggat aaatcttatg gttaaacgca attcattttt gcgcttagct

61681 ttcatgaatc atgggtcttc tattttggca tagcatctaa ttactatgtt attacataat

61741 taacacgttg cataatctgc gctacacaac ctacatactt acatcctaat aagcccgcgc

61801 cttcggccct acactaaaga tgcatcatgc atgaagatat gaagcacaaa aattaccact

61861 aaattatcta tcaagtactg ggatacttag gttcttttat ttgaacttag gttcttttat

61921 ttatcaccta cgggaaaaaa aacaagaata taccaaatga gtttgaaaaa tacatggcta

61981 tttccaattg gttattgtga cgctgcggaa ccttggcaat taggatttca agacgcagca

62041 acacctatga tgcaaggaat aattgagtgc gcctagatat atcatcacgg ttgcagagct

62101 ctgctccaac tctgccatat ctgctcgagc tggctatcgc caggatgtga gctacacagg

62161 tggggcatcc ttcaataaga ttgccccgaa agcatcatgt gaaactcgca gaacattgag

62221 actgccctca ctaggatgct tcgacaaagc ataaggaaag atcaggataa caaacttgat

62281 acgtgaatct agtccatcca attccgggcc gtatgtctga agcagaatat caaggcatac

62341 gcgtggatag taagcctgca caaaacggcc gaactcgcgc tcgatatcaa ttgcgaacac

62401 gggacttgag tccccacgta gcactctata caggaatagc ccgagaaatc aggcattcac

62461 gcaaggatct aacccaataa aatccgtgat ggtggaagct ggggaactaa ctccctgtac

62521 agggagaatt cagagatccc gtagtaagaa tgcatagttt tagctattaa ggggatcaac

62581 ctaagaccgt ttcgtatcct ctctgaataa ggggcttaaa aaaaaaatat atatctattt

62641 tttcccgtgt ccttatctca gttaaaaagc gaataaaagc ctataaatac gagaatgaag

62701 catacaatgg actgttacgc gctcaacgat actatcatct taactacgtg ttacgaagca

62761 atcagtttat agcctcgctg atgccactgc gcaatagttt gtggaataaa gcaagcaaaa

62821 aaaaatatat atatattttt ttttgcttgc ttctgtgcaa gcttccctgc tgactgattg

62881 tttaacatat gcccactttg ttcgcaaagc gaacaaagaa aggtgcgcgt agcgccgtta

62941 cgtttcatat tttgttttgg agaagagggc aaagcatgct tctttgcccc tccccaggcc

63001 gaggttcgag gtagcgagct ttatgacgga aaactgtcac gtatggttct gagggcagac

63061 accgagcgct gacccctatc ttacatcatg atattttttt cttcttaata attattttga

63121 tctttgtatt atggatgttg gttcgcgctt tatggcattt tcactataaa agaaatccaa

63181 ttccggaaag gattgttcat gggactacta tagagattat ttggaccatt tttcctagta

63241 ttattctgat gtttattgct ataccatctt ttgctttatt atattcaatg gacgaggtag

63301 tagatccaac aattactatc aaagctattg gacatcaacg gtattggagt gcgccctttc

63361 acaagaatga tggaagtgca acgaaatgca ccggactggt tctatggtct gcaaagcttc

63421 tggcttacca aaagaaagat gagccaaaat cattcttcgt ttgacgttga aagactcaag

63481 ggactagagc atgccagaaa cggggagttg aggttaaacc tatagactga aaaggctccc

63541 ccagctaata ggcctatggt tccattcttt aagtcgctgg aggtacacat tcctcttctc

63601 ggtgtaggca atagacgaga aataaactgc tcagcctgca atgtccaata acagcgctga

63661 agtatttaat ctatcagcac cacagccagt ggcatacgac ttcgaatcta acaggcccgg

63721 ccccgtcatt tcttggaata ggggatcccc taacgttggc aacaaccacg gtaatggcaa

63781 aactgccggg agaagaagaa cgagcctctc tgaggcttgc tcttcttctt tggggacgga

63841 ggtcctccag taggtaacat caagaacaag aactttaccg aaggggacca gcgcttatac

63901 tgtactgaag tctcggccgc tcgcaaggga cgtcgggcaa tttcggcgaa aactcaaggg

63961 tcacagagga tttacttacg gtggaaatgt taatatttta gtctattcaa cctaataaag

64021 agttacaaaa ctgcatatcg caagatcaaa tgaaaatttg ggaacattcc ttcaggagtc

64081 ggcgagtcca ctctcgacga ttcagggcgt gactcgtctt atcatcattg ataaaatgaa

64141 ggacagatcc tttcggttcc aagcaaccgg taagtttttt aaaacatcaa taagtactaa

64201 gaacaggaag gaaacgggaa gcctattgct ataaaacaat gagcaaggac ataaggccca

64261 aagattaata aataaaccat gggttcactg ggtcattcct acaacttgga ctatcaccct

64321 cttttttcga acgttccact tattgaacaa aggataacta gattggattc gttttatgtg

64381 gttaactatg cagtaaggtc ccatactttt ttcttccata cgatttgtgt cttgcttgta

64441 aaattactaa aattgcagaa gcacattatt acagacatat caacaaaaag gccaaagttt

64501 tgaccttgaa aggtttcact aagatcatag ttcaataaaa caagaagcag acccctgata

64561 acgccacctg aagatccact gtagagagta tattacgttt tatccccggg tgggaaagaa

64621 ctagacattc tactcttcgg gttttaatga gcgtggagag ctgtctgcgg ggaaacttgc

64681 aagtacagtt tggtgggagg cgtatgggcc cttgggacca aggactggcc gtcgacccaa

64741 ccttatgagt attcagacta taacggttct gatgaacagt cactaacttt tgatagttat

64801 atgattccag aagatgactt agaattgggt caatcacgct tattagaagt agacaatcga

64861 gtagttgtac cagcaaaaac tcatctacgt atgattataa catctgctga tgtacttcat

64921 agctgggcta taccctcctt aggtgtaaaa tgtgatgctg tacctggtcg tttaaatcag

64981 acttccattt ttattaaacg agaaggagtt tactatggtc agtgcagtga actttgtgga

65041 actaatcatg cgtttatgcg tgcgcccgaa taaataggcc gactgctgag cccattctgg

65101 ctcagtcgca ctttctcgaa caaggcaaac ctgggtgcga gctacccaaa aaagctatca

65161 tagcaatatc atggctagag cagtagggaa aagccgggga tcgatgggcc tgcccccatt

65221 agggctcccc gggaaagcag aggatatggt taggataacg agcttgaaac gcggagcccg

65281 tcctaagctg gaccgaacgt cccaagcagg atatagcggc gagcgagtgg ttagtaaacc

65341 aatagtgtta aactcgcagt tgatggcatt agcttctgcg gcacccgagg aaccacaggg

65401 cactccatac agagtaagtc cgagagatca gacgcccgcg caaggaccta aattctcact

65461 aggaggtaaa ccctaattcg gacctatgga agtcggggct aaacatcccc atgacagtgt

65521 cgtgagggag ttcagaggcc tcatagtatt acaggcctct tcaggtcatg cgaagggggc

65581 caatccaaga tcgtactttt cctttactaa gacaacagga gctctcaaca agtctatacg

65641 ctagtttacg ctcaagttaa actggacaga tcttgtttgt ctctcaacag ccatataggt

65701 aaggatctac aaaagcaaac acggcagata ctacggattc acccgaatat tgagcgattc

65761 tttgtctggt acgaggctat ttgaaaaaag caaaaaaaaa tgacaccgag atctgtaagg

65821 aatactctaa atagaaaaaa aaccccagcc catttagtaa ataagccgag caaaagtcag

65881 ttatttttca caccaccgga aatacccaag ccgggcgaga tcggaacggg ggcagcagca

65941 tcgtgtgaaa agacctccgg ctaatgctgg aggtcatatt ttttgcctat tttatttggt

66001 cgctcgcgcg gatttcgacc tcacagagga agccaaggat atgttccaat tacacgcggg

66061 cccgcgttat ttcgattgtg tactatttgt agatctaatt gagaggatat acatagaggt

66121 aagagacgta agagctaaaa ttcgcgcggg aaatgttacc tatgactagt gtaagtatga

66181 aactgctgtg tgggcaacaa ataccacgac gccgccaaca gtgacttgtg ggctgcggcg

66241 cagattaaga tactgagaac tctaactatt gtggagaagg tcgcctaagg tccaaggtta

66301 agatctagga aggtgagcag tacgagctga aaggctccca tactgttcgg agggcagggg

66361 cttttcctga cccctatcca ttgttgtaga agctgtttct ttggatgatt atgtttcttg

66421 gatatcaaat aaattagact aaaaagcaaa caggacgaat tatgagtgtc tctcaaaagc

66481 atccttatca tttagtagat ccaagtccat ggcctctttt gggttcattg ggagctttgg

66541 caagcaccat tggtggtgtt atgtacatgc actcttttat gggaggtgga acacttctta

66601 gcttaggctt gggaatgatc ctatatacta tgtttgtatg gtggcgcgat gttatacgtg

66661 aatccactta cgaaggacat catacagttg tggtccaatt aggacttcgc tatggtatga

66721 ttttgttcat cgtgtctgaa gtcatgtctt ttttagcttt ttttcgggct ttttttcatt

66781 cttctctggc acctacggta gaaattggag ctattcggcc ccctaaagga attgatgtgt

66841 taaatccttg gggaattcct tttctaaata cccttattct actttcatct ggagctgccg

66901 tgacttgggc tcatcatgct atattagctg gattcaaaaa acaagctgtt tatgctttag

66961 tagctaccag gcgaccgtca gattaccaag gaaacttttt gattacctcc tgtaatcata

67021 ccattgctga tgctcgcaat gagacggatg caacttacca tttgaaccaa ccgggacaat

67081 agcatgttgc aaaaggaaag gacagggttg accaactcta gagaactttt ccgaccgtgt

67141 cttggaaata tagccgaatg ggtcattcat gaatgcgagg tgtttatgag acactaactc

67201 gagcgtgttc ggtcaggtta ttgatcaatc aataatatta cagcgctatc tcctccatgg

67261 cagaatggta gcctgcctgt ggcagagcag gaggaggtcc caatttcaat atgaaacaaa

67321 aacactggaa gcacggctgc ttttttgtcc aaactagcac tattagttgg aaatcttatg

67381 tgttttcatg taagtataag agtaccttgg tagtaccggt gaactagata gccatgatcc

67441 ggctatctgg gacggaggac tcgtagtatc cgcctacttg aaagagagct ggcaacagta

67501 aagcacttga ctcgatctca ttcgtttaag ggcaaagcga gaaggagtct aaatcactgt

67561 acagaaatga ttttcattta tggactttac ctgattgaca cgggagatct gacttcatgt

67621 ctgaatagaa ttaagcctaa gcctttataa aggactacgt gccctataga gtaaaaaatc

67681 aggttggtga gccgtgtgat aggtaactat cttgcacggt tcggagagca ctttattatg

67741 tcagatgagt aaacggcgac caagttgtac ggctctatga agtaactttt gactctacca

67801 tttcgctggc tctagtcttc accgggtttc aaggaatgga atatgtagaa gcacctttca

67861 cgatttctga tggtatttat ggttctacct tttttttagc tacagggttt catggttttc

67921 atgttattat aggtaccatt ttcctaataa tatgcgctat tcgtcaatat ctagggcatt

67981 ttacccaaac gcatcacttt ggctttgaag cagctgcttg gtaccggcat tttgttgatg

68041 tggtttggtt attcttatct gtatctattt attggtgggg aggtaattaa aaaaaggaga

68101 aaaaatctga aacagttttt ttaccaacct aatcatactt tatttaaaga tagaacttaa

68161 tttagtctgc ttttttttct aagaaccggg cttataatga tggtggtatt cgtgattaat

68221 tctggcgatt ctaatatgga tcctagtact ttctagaagg aaggtatcca tataccggtg

68281 atgtcacgag gcaaatattt tgatagactt aaaagttatc aataacttaa ttattattct

68341 aagaggaagc ttgaggtcta taaggggcat tatgatgtca atccagagag cataaaaggc

68401 atcccgacct tgctgctgaa aaaataatat gtttgctatg acctatcacg taatatgcaa

68461 cctgcctttt tttccaatag cagtcgaatg gataaaaccg tagctgctta aaagcgaccg

68521 ttagatttgc ataagaaaag cgaagaaggt ggttgaccaa cgccgcgcgg cacgcgcgtg

68581 cttgtcctag gccggtttgc ttagggtgtt gcttacgcgg cgctttggag aggcttcgtg

68641 ttgatggtat accagtaaca tttttgtggg aaccgaataa taaatatagc ttcgcggtct

68701 tcttcgtcca ttatgcgggg ggtgcgcggc ttatgtgaga acccgcgcgc ccccctcccc

68761 ttcctttttg tctgtttggt cttgactgcc tcgttcgtaa aaagcggcat tatgtaaagt

68821 ttacatacat attaaaatag atgggctaga tgcactaaac aataaaacta cttcattaat

68881 tctaggaaat gcttctatgt attttggaaa gatgcgtagc acttggttgg ttttgcaaac

68941 aaagaaaaaa aaatatatat atattttttt ttctttgttt cactaatcag tagaaaaata

69001 tgctatgctc cgcggcctag ttcattttac gagcttgttg gattagccct gaattgaatc

69061 aaagtttttc aaaggctttt cagccggttc tccgattaaa ctaagaaaaa tagaaattaa

69121 tgcattcttc ccgcgcaagt gttttgtcaa tgcctaaaag caaggaacca caaacccgcg

69181 gcaaaaaaat atatatatag ctccgcggta ggtttctgtt ttctggctat agccaattaa

69241 ggtgtcatgt ccataattct tatgacattt cacaacttta ttttggggct atcattcatt

69301 caggctactt taagcggttt aactttcaat catccgcctt atcctgttag cccgcggtcc

69361 acaacctatg tttttctaaa gctattgtaa aaaaaatata tatatatata tatttttttt

69421 aacaatagct tttcatatga aataggatag aaaaaaaagc caacaaaatg agactgtata

69481 tcattggtat tttagcgaaa atacttggaa taataatacc ccttttacta ggagtagcct

69541 tcttagtttc agctgaacgt aaagtaatgg cttctatgca acgtagaaag ggtcctaatg

69601 tagtgggatt gtttggattg ttacaacctt tagcagatgg tttgaaatta atgataaaag

69661 aacctatttt accaagtagt gctaatttat ttatttttat aatggctcca gtaattacat

69721 ttatgttaag tttggttgct tgggctgtta taccgcgcgc cgtgcaaggt gctttcgtcg

69781 ctatggggca tatcctggtg cccgatctcc agtctgcgtc aatggagtaa atcacccaga

69841 ggtagcgttg ccgcaatgcg cgtggaaaag catctgggaa accaagtcac aggagatcca

69901 tatttcaaag gacgactcgg aagatactgt tggggttgat gaggctgacg aatccgaatt

69961 acgtagctgc tgaacgacag cattcaccaa gccagcgggt aacgacttgg tcctgtaatc

70021 ggttcttttg gtaggtataa cggaggccga agacggaaaa aaatatatat ttttttttcg

70081 ggggcattct cagtaaggga ataatactat gcggacatct tacctcgacg gacaggtgaa

70141 aaaagtcgaa gacgcaatca cgggatcgac ctactctcta gtgagagggt cggcggagcc

70201 gctatagtaa gtaaataggg aaatcgacca agccaggtac tgaagggcgg cagtcatgat

70261 tcgatggtag agggcgtagc cctttccttg ccacaaatgt ggtgaaggcg gcgacgcttc

70321 aactcttccg agaagacggg ttgatcatag cagacactta aatgctgcta cgatctcatt

70381 caataagtac ctcgtaaagc gcgtcaatat atatatatat ttttgttgat gtgctttagt

70441 aaatcagtgg cggagagctg tatgacggaa aactgtcacg tatggttcgg agggcgggga

70501 aatctccgac ccctactttt gattatggta tggtattgtc agatttaaat gtaggtatac

70561 tttatctatt tgctatatct tctttaggcg tttatggtat tattacagca ggctggtcca

70621 gtaattctaa atatgctttt ctaggagcat tacgatctgc agctcaaatg gtttcttatg

70681 aagtttctat cggtcttatt attattactg tactaatttg tgtaggttct tgtaatttta

70741 gtgagattgt aatcgcgcaa aagcagatat ggtttggcat tcccttgttc cctgtattta

70801 ttatgttctt tatttcttgt ttagcagaaa ctaatcgagc tccttttgat ctaccagaag

70861 cagaagctga attagtcgcg ggctataatg tagaatattc ttcgatgggt tttgctcctt

70921 tttctccagg ggaatatgct aatatgatct taatgaggtg tggagctttg catctgacat

70981 tcgttgggct gcggccttct gcaggagcca gtgtcctttt aagggccttg tgcagtaaac

71041 ccttctgagc gatctgaaaa ccagtaggct cgcgaagctt tcagagaagg gtagcctggt

71101 gtgtcagcac aacaacgaaa cgcgaaccca tcggacgacc tatctaagac tagggagata

71161 ccctaagtgg gtacttcgta acttttcccc agacctatac gtgtaccgaa tgctcatacg

71221 ggaaagtgcg ctcctaggtc tggaaccagg gagggttgct gcgagaaaaa acttctcgtc

71281 tcatccaggg ggtgcgaaca cacctgcgcg aattacagat gacagctaca agggtggcgg

71341 gggaaggaaa cagtacccca tatccgggga tgaatgtaaa cccattgaac aaggataatc

71401 attctggttc tgggagatag aactcaccgc cggtggtgga cattagtctg aaaatcgggc

71461 gtagcgatcc caagtcacta gggcgcaaag cgcagcacct atattatcgc ggacaataat

71521 agactactac tcgcgcctta ttacgagcga atccagtcta aaccgagcag cttaaaattt

71581 gacggaaaat acatttttcc cgctctgtgc tgatcatcca cactcagaca tccatgcgag

71641 gccttcgtga ttcgaaaacc tacgcgtagc attggttaga gggggatgag actcaggatt

71701 tccagaacgg agccgtatga cgcgagagtg tcatgtacgg ttctttgaga agggtgtgaa

71761 tatctattat tctcaggccc caaggggcca cgaagctaca cccttactct cctagtctat

71821 gtacattgct ttttctagga ggttggctgc ccatcctaga tattcctatt ttttatgtga

71881 ttccgggttc gatttggttt agtatcaagg ttcttttctt tttgtttgta tatatatggg

71941 ttcgtgcagc atttccacga tatcgttatg accaattgat gaggcttggt tggaaagtat

72001 tcttaccttt atcattagct tgggtagttt ttgtatctgg tgttctagta gcttttgact

72061 ggctccctta attcattgaa caatttcact gcagtgcaac taaaaaatat atatttttaa

72121 ttaaaaaaaa ctccgttaaa tagcagctaa aaaaccaaat gaattgatta gaagaaatat

72181 aaaataatat attttattcg ttctattaac ttcataaatg cgggctttga gagaaggaga

72241 gagaggccaa gcctctctct tttcttgagt gttccaaaac gaataaaata tatatatata

72301 tatatatata ttctttttat ctaaggcctt gtaatgatgg gtaaatcctg cccatgatgg

72361 attgcgttaa tcatgaagaa cacaaagttt ccatgatccg cgaaaacgag aaagcacgaa

72421 acattcatat ggcaagacga ctatcgattc ttaaacaacc tatattttct acatttaaca

72481 atcatttgat agattatcca accccaagca atataagtta ttggtggagt tttggttcgt

72541 tggctggtct ttgcttgttc attcagataa ttacaggcgt ttttttagct atgcattata

72601 cgcctcatgt ggatttagct ttcttaagcg tagaacacat catgagagat gtgaaaggcg

72661 gctggttgct tcgttatatg catgctaatg gggcaagtat gttttttatt gtggtttatc

72721 ttcatatttt tcgtggtcta tattatggaa gttattcgag tcctagggaa ttagtttggt

72781 gtcttggagt tgtcatttta cttttaatga ttataacagc ttttatagga tacgtactac

72841 cgtggggttg atttggcccc tccttctact aatggaagga taaaaaaacc ccactaaatg

72901 cgggaaactc tttattacta aggtactttt ctcctatgga aggcgcgaaa tggacgattt

72961 ttggtggcga tccccagaat tttagccttg ctgtcttgtg tgggtttaaa ttctaagtaa

73021 aaatccttag catgtcatca tagacaatcc gcaggaaaac tcttgctaca cgagaatagg

73081 cgtcttcggc cctagaaaaa atagcataga gatttcctca gagactacac gtggggtgcc

73141 tagtctatca ttgatctttg gctaggtaaa tatatagtcc catttctctc taaaaaatca

73201 tgtctatttc actctaatga atgaataaat aaaggccgga ggcctattgg agtcttctta

73261 tggtctattt aaaccgtata ttaaggtttt atttataagc cttacttaaa cagctttgta

73321 accttttgcc ataatagatc caggatattt taatagggtt tactttcaat tcttgctccg

73381 gggatatttg agtaacaccc aatttaacgg ataatagtaa ggccgaaggc ccgccagtat

73441 ctaggatttc aaatcaaaac ctcggctagt tcgaattggt tcctgttttc tttcagggca

73501 gttaagagag tttctaagaa aattattgac tattcgactc ttggagcatt agcctattgg

73561 cttatgaatg atcaggccaa agagcgcgcg gcccttattc atacaaatag ttttaccaaa

73621 atactgcaga aaaaatgtcg tatcagggct aattctagta aaaaatactt taaatcgctg

73681 ctcgatatcc tgagtgaaat gctgagatag aaaaaaccgg tggagcctga catcattcat

73741 ccataaaatc tgaaaagaat gtagaaaaga catggtttgg gggaatttag gcaaatgagt

73801 ttttggggag ctacagtcat tacgagctta gctagtgcaa tacctgtggt aggagacact

73861 atagtaactt ggctttgggg tggtttctcg gtagataatg ctaccttaaa tcgttttttt

73921 agccttcatt acttacttcc ttttataata gctgccgctg ctattattca tcttgctgca

73981 ttacatcaat atggctctaa taatccattg ggtatcaatt cttctgtgga taaaatagct

74041 ttttatccct atatatacgt aaaagattta gtatgttggg tagcttttgc catttttttt

74101 tctattttta ttttttatgc acctaatgtt ttggggcatc ccgacaacta catacccgcg

74161 aatcctatgt caactccggc tcatatagtg ccagaatggt atttcttacc agtttatgcg

74221 attcttcgaa gtatacctaa caaattaggg ggtgtagctg ccataggact agtttctgtg

74281 tcattattcg ctttaccgtt tattaacaca tcgtatgtac gtagttcaag ttttcgacca

74341 attcaccaaa aattattttg gttgcttttg gcagattgct tagttttagg ctggattgga

74401 tgtcaacccg tggaagcacc atatgttact attggacaaa ttgcttcagt tggttttttc

74461 ttctattttg ctattacgcc tattctcggc aaattagaag ctagattaat ccaaaattct

74521 aatgtttgcg aggacttaag tcctcgcaag ctttcccaca tttttaagaa ttcaatttat

74581 gttgtgaaat gaaaagccat caatttatta accgtcttat taccaaatcc ccgtatccgg

74641 tttttatcca tgatttctgc attaattagt ggtgtgtttt taattgcgcc acttttcaaa

74701 cccatcattg cactttgtga aaatgggttc ctgaatgcag cttcgccctt ggatgctcac

74761 gcccttgccg ggatcactct agcaattaca gcgggtattt caggaatcca gcgtcggtca

74821 agaattccgg atcaaacaaa gtttctgtcc agaattgttg acctaggaga cccccaacaa

74881 ataggggccc atgaacgttt tgtttggttc aattgaatga tcacagaaag gcttctattt

74941 gtatttcgct atttgttgtt gtcgtcgcca taatagattt caattcaatt ggatgccgac

75001 aaaataaaac aaatattaaa caacaataga aattgacttc gtgttgtttg tcttattgac

75061 ttctttgtct tatcaaagaa gcagcatata ttgctggtaa tcgtgttgtc ttattgactt

75121 cgtgttgtct tattctcatt ctctattatg ttcgctgcgc acataatagc cttttagagt

75181 tcagttcttc tttttggaaa gaaattacaa aaagaaatta caattacaat acattacaag

75241 atgtatttgg ctattacaaa tacatcttac agctattata ataagcttct ttcctttctg

75301 tgttgtttcc tttctgtggt cttttatttc tgaagtttac taagtttaca aggcgcaaag

75361 cggaattcgt atatttttat ttgcaaataa taattaacat tttaacattt gcactagaga

75421 ctaaatgcta cacaacgtat tcactgtgct gcgcacataa tagattacca attttgcagc

75481 ttcacccaca gaaaggaaac tgaatattca gttaataatt ttctctaaga aaagaaaaga

75541 actaaaatat gcttcgctct ttaactcatg ttctaatgtg tttacttctt ggaaaaaaag

75601 agacgatttg tggataacca atcgtttttc aaatctctga tagctacttt accaaaatgg

75661 atacatcaat ttcaaaaatc aaaacatgaa aatatattat ataccaatcc tgattaccta

75721 tttcaattat tatggttttt gaaatatcat accaatacac gttttcaggt cttaattgat

75781 attggtggag ttgattatcc ttctcgaaaa caaagatttg aagtagttta taatttacta

75841 agggcgaccg cgaagtcatc gaataaagtg ctcattcgta ccaaacgaat gagacgttgt

75901 agaagtctgc aacgaaacgg gcacgactta tttgacggat ataccgctag ggacacccaa

75961 cggaacgaac ttccaatggg gaacatagcc tgtcgtgaaa ggggatcaca gggaatagcc

76021 aacccataat acccaaccgt gtctttgaaa cgcagttgaa cggggaaaaa aatttatttc

76081 ttttttttca ttcgtaaacg tgaggtgtag gtgggatatt agcccgggcg cattaggcaa

76141 gactcgaatg aagtatcata gcgtcttctt cgtacgacgg agcttagtga cgatcgtacc

76201 aggacaacaa aggaaccaag atccccaaaa gtattttttt tctttttgct ctcatgaaaa

76261 tcggagtaaa gggcgaagct ttaaaggcac agcacttgag ggtatctaaa gcacctgaag

76321 cgcactgcgc gaagatgccc aaaagcatcc aattacgagc attcggtgga accggtgaac

76381 catacatttt tctaaataga gatgcgtggg acagagggct cgtagtacct acctacccaa

76441 aacaaaccct gcaaaaggaa agcgctggca ctgtatgacg gaggggaagg agcctaaatc

76501 gacgtacccc ctcctagagg gggtacgttg catactcgag cagcatgaag ggaccgagat

76561 tgagcttgga taagactaag cagttaaaaa aaatataaat ttttttgctg cttcgacata

76621 ttctaatcgt ctgcatgttt tttgagaaca ttgtcataaa gagcagttcc agacagaaag

76681 cctttttcag cttataaagg gtatcaatct gtggattacc gcccataaaa agcttttacc

76741 taattaaggt tcgaagaatg gtgcttatag gaaaactact atatgcgcca ctttgatcaa

76801 agtaccatga acactggggg actaggtaat ccacttcgga ttacatttta aagcaaaaaa

76861 cgctaaaagc gtgcagcaaa taatttcttt tttttatgta gctttcctcg gccacactgc

76921 gatacataga gccataattg aaacagtagg aaacctcagt ataagccatc cgcaagcgtg

76981 cgtttcacag atgattttac tattggggta attggtccac gagctctggc agaaaagata

77041 cttgacttgg ttacataatt tattgaagta cggctttagc tcaggcttaa cctggatagg

77101 accctaataa cctgaaccaa aattaataaa atttctttcc ttggatatct tactagtcac

77161 ctacaagttt tgacaacaat acggcggcaa ttggataata tatagaatcc atcaatctgg

77221 tgggcttagc ttacttgtca atatgtagaa aatgataaac cgtctggcgg ttttctgata

77281 aatgaagtaa cccgaaaccc tgttttgctt actttcagta tttttaaagc cattcagtag

77341 tgcgcgttaa cctccattct actctgccct gtcaattagt agcatctggc aaactctaaa

77401 aaccaatgta taacgcaccg cgcttaagct acatactacg tatttcattg gccaaaacat

77461 atacgaatta tacaccacgg ctaataaagg caaagcccgc tcgaattgca taactcattc

77521 gcttacaaaa attaccacaa taagcaatac gagggcgcag cacctctgga agccatatat

77581 gctgctagtt tttgctataa tgcaaggcta cgagtgctcc atgtacgtaa aaaatatatt

77641 atttcgccgg gaaaacccac gcttagagcc atatgattac aaacaacctt agttaaactt

77701 gagttggaga gccgtgtgat gggtaactat ctcgcacggt tcggggagca ctttattcta

77761 ttgagagaat gcataggaaa actgcggctc tgtgatggaa ttcttgactc tatgtattca

77821 atataactca cgcattcgtg tacaaaccag tgtggacgaa ataactccaa tttgttcggc

77881 agtcaatata tttccgtcag cgggctggtg ggagcgagaa gtttgggata tgtttggtgt

77941 ttatttttct gatcatcccg atttacgccg tatattaaca gattatggtt ttgagggtca

78001 tccattacga aaagactttc ctttaagtgg atatgtggaa gtacgttatg atgattcaga

78061 gaaacgtgtg gtttctgaac ctattgagat gactcaagaa tttcgctatt ttgattttgc

78121 tagtccttgg gaacaaagtt cgcgtagtga caaatcgagg aaaaagtaaa gaatttttgc

78181 ttacagccat cttgataata ttcaatttcg tagtaattgc atgctcggct cagttctatg

78241 gcatgctgaa taatccgcct tgcctgtctg aaccaaactc ggtcttttcg atctaaaaca

78301 gcgggagtgt tgacctttta tggaaacgcc gcgctcgggt gatgaggatt caaaagaata

78361 aagtgggcct ccgctacttc attagcttaa cagagaaaag aaggatgaaa agaaaagaat

78421 aaaaaaaaat atatggaaat gccccaaaat tttttatcta ttttgccttt catcttcttt

78481 tccttatgtt ttattagctt gaaggagctt ggctaatgaa tgcctccccc cttcccgtct

78541 tgatctatca tttaagatga taaattggac acaatctgaa ggttcagatt gtggaattat

78601 ttaatttatt ggtagaaggt tttattaatt tatgaacaaa ttaactggaa ataagttggc

78661 tggagcagaa ctatctacat tattagaaca aagaattacc aattactaca ccaaattgca

78721 agtggatgag atcggtcgag tggtatcagt tggagatgga attgcacgtg tttatggatt

78781 aaacaagatt caagctggag aaatggttga atttgccagc agtgtgaaag gaatggcttt

78841 gaatctagaa aatgagaatg taggaattgt tatatttggt agtgatactg ccattaaaga

78901 aggagacatt gtcaagcgca ctggatctat tgtggatgtt cctgtaggaa aggccttgtt

78961 aggtcgtgtg gttgatgcct taggagtacc tattgatgga aaaggtgctt taagcgctgc

79021 agaacgaaag cgtgtagaag ttaaagctcc cgggattatt gcacgtaaat ctgtgcacga

79081 acccatgcaa acaggattaa aagcagtaga tagcctggtt cctataggtc gtggtcaacg

79141 agaacttata ataggagaca gacaaactgg aaaaactgct atcgctattg ataccatatt

79201 gaaccagaag caaatcaaca cacagggcac ctcggatagt gaaaaattgt attgtgtgta

79261 tgtagcgatt ggacagaaac gttcaaccgt ggcacaatta gttaagattc tttcagaagc

79321 gggtgcttta gaatattgca ttattgtagc agctactgct tcggatcctg ctcctttgca

79381 attcctggca ccatattcag gtcgcgctat gggagaatat tttcgagata atggaatgca

79441 cgcattaatc atttatgatg acctttcaaa gcaatcagtg gcatatcgac aaatgtcatt

79501 attattacgt cgaccaccag gtcgtgaggc cttccctgga gatgttttct atttacattc

79561 tcgttcatca gaaagagccg ctaaaatgtc agaccaaact ggtgcaggta gcttgactgc

79621 attacctgta attgaaacac aagctggaga tgtatctgct tatattccga ccaatgttat

79681 ttccattaca gatggacaaa tctttttgga aacagaactt ttttatcgtg gaattcgacc

79741 tgctattaac gtaggattat ctgtaagtcg tgtgagcggg gtgagatcta catgggatcg

79801 ctggagttgg aaagctctgc gtaggatccc tcagcgcgta atctgcctta ctcgattata

79861 actgggtcga aagccggtaa gtcagaaatt aactatcgga agttcaggaa aacaaacctc

79921 gatgatggtc gccctactct cagagggaag acacccggga ttctacctgc gtgaacttgg

79981 gatatgtgcc gtctgcagca tgagtacttc tactacacga gaaggaaaag gggaaccctg

80041 catcggaaaa cacacgaact ccacggcgat gaacgagtca accaaggctc tacaaatcgt

80101 taaataccta gagggaactc ccgatgggaa tccggacctg ttcataagga aaggccgcga

80161 ttcgtacggt cccagtggaa ccaccacaaa aacttacgag gggagaacct cgttggttcg

80221 gcgatggata gaactgagaa tcagaaaagt cctgcttctc ctgcccgagt tgaggctgcg

80281 gccgatcgaa tttggaaact gagacctaac gtcgacggaa aatatcggaa aatcatcaac

80341 ataataacgg acccacatgc gctcatagca gcgtaccaac gcatcaaatc taaattcatg

80401 gaaaaggagg ggatgacgaa tgggaaatct tccagggagg aaatcaactt cttatccgcg

80461 ttaatcgaag atgattcgag cacatcgcag aacaactgcg cgcaaagaaa taccccgcga

80521 aacaaataaa catcccagaa taaacaaaac ccgaggagac aagaacactt acgatgatca

80581 gcgccagaga ccagatatta caaacagcca tcaaggcggc ctttaagctc acgccataag

80641 acttaagcag aggctataag accgctaagg aagagagtaa tgctgcacag ttccgggata

80701 atgaactagc attcaatagg gagcaaatcc ctttgcgtgc ttaacattct aaaaaactac

80761 acacggacaa aatattgatg atttgattgt cctcgtctat aagtgacagg tttcaggaga

80821 agggagccgt gtgataggcg actatcgcgc gcggttcttt gagagggagc tgggttctat

80881 atcctgtgct agcgcctaga gatgggtgcg aaccctactc tcgaggttct gcggctcagt

80941 taaaagctat gaaacaagta tgcggtagct caaaaccaga attggcgcaa tatcgtgaag

81001 tagcggcttt tgctcaattt ggttcagacc ttgatgctgc tactcagtat ttattaaatc

81061 gtggggcgag gctaacagag gttcttaaac aaccacaata tagcccaatt cctattgaaa

81121 aacaaatagt ggttatttat gcagctgtca aaggttattt agatcaaatt cctatttcga

81181 gcattaataa atatgaacat gagcttttga agtctataga ctcagatata ctttctgcta

81241 tcgtacaaca aaaaaacatt accgagcaga ttaatagtca actggctacc ttttgtaaaa

81301 aatttacaca gagcttctta gcaactcatt cagtttagaa aaatgaaact aaaaaaaaat

81361 tttcaggccg caggttttgt tttcgtgctt ccgggtggag ggtgaaagcg gccagggcgc

81421 agccaatttt ttttcttccg ccctctggct acgctcctag tagggcttcg tcatacgagc

81481 ggagctcgaa aaccctccat ccccacatta acaacaagta gatttggaga aaacaaaaac

81541 gcaacaaaac attaacaaaa ttgaatatat agaacgcttc tttttctaat gtactatttg

81601 taggaaaagg gctttacgcc tttttatttg taccatcaga tattatttac gtatgcgtta

81661 tctttgccca ctatttgggc ttgcgcttag atagatgttt gcatcagtct tttctttctt

81721 ctaaaatgaa tcaatcattt tcgatgattg cttcgccctt caccaaattc tagctggtgt

81781 aatcaggaga aaagggattc gaacccttaa cctgtggttt tggagaccat tgttctacca

81841 ttggaactat tctcctaaaa aaaaagtggt tcttggttta tggaatttgc acctatttgt

81901 gtctatttag taataagttt gctattttct ttgatcttaa tcggtgtttc ctttctattt

81961 gcttcttctt ctaattcggc ttatccggag aaattgtcag cttacgaatg cggttttgat

82021 ccttttgatg atgctagaag tcgtttcgat atacgatttt atctcgtttc tattttattc

82081 attatatttg atctggaagt caccttttta tttccttggg cagtttctct taacaagatt

82141 ggtttgtttg gattttggtc tatgatagta tttttattga ttttgacgat tggattttta

82201 tacgaatgga agaagggcgc tttagattgg gagtaacccg gcaatttctg agccaaaaaa

82261 cgataaaagc aaaaaaacag tttttttgct tttatcgttt tgcaagcttt tagcattcct

82321 tctatcgctg cgtaaacaaa atgggataaa cctcgataag taggcataat aagtcattca

82381 ttaactttat tgagctctcg gagcctttgt tggctacgcc aacaaagtgc agcccacggc

82441 aagagagccc gtaaggccgc accggctctc taataaaatg aactgaaagg atgctgggac

82501 gttaaacgaa tcgagcaggg cgctgccaaa ttagtttgtt ttcgtgcgtt tgattttttt

82561 tgttttgttt ggtagtttaa tatctttacc ctatcgggtg gaaaatatta aagcgtttcg

82621 cggaacaatg actgtctgtt cccgtacagt gaatgcttaa aaacaataga atagatcttt

82681 ttgcgatggg ggaagcccga aaaagcggct gggagggttc gcacaacgaa tgaaaggtga

82741 aggtagtttt cctacttttt cctctcgcca aagagcttct gaataatatg cttcgcttcc

82801 cccgcgctct ttccaacaaa atgaaaaaaa agacattata tattaattac atagtatact

82861 caattctata caatcaataa agagattagc gcaacctacc ttttgttgat gctttatgct

82921 atataagaaa aaagtggtaa tagaaagaaa aaaaagaaat atttttttgc tatgcttttt

82981 atctttaagc tttacgctcc tactttcggg tccggccttt tatccgcttt actttaatca

83041 ataggctgga ggaaccactt tggtggcgta gctgtgaaag atcaatttga gtgatgtgca

83101 ataagataaa acatcaagca attggtaaaa agtgaacacc tttaataaaa aataaactaa

83161 tgatgatgtg ttctttttta attgattatt tagcatatta gggtaattag ctcagttggt

83221 agagcgcctc gtttacaccg agagagtcag cggttcaagt ccgttattac ccaagggttt

83281 tcattatcca tgattctaaa atggaaagat cccgatgaat catcgtagat gattcattat

83341 gtttgggaaa atagcttagt tggttagagt gctggtctgt cacgccagaa gtcgcgggtt

83401 caaatcccgt ttttcccgta ggggacgtag ttcaattggt agagcgcatg ttttgcaagc

83461 atgaagctgt cggttcaact ccgatcgtct ccaaataaac aagtaatata ttgtagaaaa

83521 gcagtataag tgcttggatg aattacgctt tataatagcc gcaggagatc tcgttatgct

83581 ttgcacttta acttggcctt ggaaaaaaga atctgaaaac caaactgaat aatttgtaat

83641 aaaacgtgtt aaaggtaaag atggaggaca ctacgcgttc ttgtaacgct ggcaagataa

83701 atatttggct gcgctctatg tgttaagcca gcttaaatta cgatattttt ccttaataat

83761 acaaacagtg gctatatatt ggcctgcgta gctcagatgg tagagcattc ccatggtaag

83821 ggaaaggtct ccggttcaag tccggttgta ggctctataa aaacaaaaat gattaaatat

83881 ggctaaaacc aaacaaatca aaaattttac tttgaatttt ggacctcaac atcctgctgc

83941 tcatggtgtt ttacgattag tattagaaat gaatggagaa gttgtagaac gcgcggaacc

84001 gcatattgga ttacttcagt gcggcatgaa gccgctaacg ctgagtcccc actgtggttg

84061 acttaccact gggctttctt ggaaacgaga acgcgtcggc gggtcctgga gtacccgtca

84121 ggggcgcacg cgtattcccg cggggtgatt attacgacca gcccctccgc atcttagcac

84181 agcggaacgt gtaaccggcc tgaggtccca tttcaaccgc caatttattg tccttacaat

84241 gggtaggcta accacaaggt acaagccaaa accttaggtc gaataggacg gcactactgg

84301 caaatactat ctagcaaaga cacgaatcat gaaggataga gcttgcgtca aaagcatacg

84361 ggaaagttct agcaacgggg aaaccggagg gagaaaccag tagagccgca agagcataac

84421 cggaaggtta agccagggag gctgggtcat ttaaaaccat acatttttct aaatagagat

84481 gcgtgggaca gagggctcgt agtacctgcc tagcctttgc ttcgagaacc atgtaaacga

84541 agaaaggaag tgctgctgga aagcgaaagg aaagaggcct aaatcagcag actgacgccg

84601 cgcacttgag cagttcggag aagaccggcc taatccagac tctttagaaa ttaagccaga

84661 atgcgcaact ttcaagaaac gaaggaagtc cgttaatatt tggttcgttc gctagcgcat

84721 aaaccaggca gacgctttat tcgaaccaga gcttcatcac ccaaaagcga aaatacttct

84781 gaagtcaaaa ctcaaccatt atgacgctgc gctcctggtt tgcttattta aaatttaagg

84841 gtaactcaag ttagagagcc gtgtgatggg tgaccatctc acacggttca gggagcattt

84901 tattatgtga tgcgagtgaa tgtgtacagc atagctggca tcaattaaat tttgacttta

84961 tttatgttta tatgatggtc caagaacatg cttattcttt agccgtagaa aaactttata

85021 attgtaaggt accattacga gctcagtata tacgagtgtt atttcgtgaa ataactcgaa

85081 ttttaaatca tttacttgct ttaactactc atgctatgga tgtgggggca ttaactccgt

85141 tcctgtggcc ctttttgaag agcgagaaaa actttcagaa ttctatgaga gagtttcggg

85201 agccaggatg catgccagtt acatacgacc aggcggattt gcacaagaca tacctttggg

85261 cttaagtgaa gatatttttc tatttacaca acaatttgct tctcgtattg acaaaataaa

85321 agaaatgtta accaacaatc gtatctgaaa acaacgatta gttgatattg gtactgttac

85381 tgcacagcaa gctttggatt gggtattcag tggtgtaatg ttaagaggct caggagtatg

85441 ccgggatttg cgaaaataag caccttacga tgtttataac caattgattt tcgatgtacc

85501 cgtaggtacc agaggagatt gttatgaccg ttattgtatt cgtattgaag agatgcgaca

85561 aagtattcga atcattatgc aatgtcttaa tcaaatgcct agtagcatga ttaaagcgga

85621 tgatcgtaag ctatgtcctc cctcacgatc tcaaatgaaa caatccatgg aatctttaat

85681 tcaccatttt aaactttata cagaaggttt ttctgtacca gcttctttta cctatactgc

85741 agtagaagca cctaagggat aatttggtgt gtttttagtc agtaatggaa ccaatcgtcc

85801 ttatcattgt aaaataagag cacctggttt tgctcattta caagggtttg attttatgtt

85861 caaacatcac atgctatcag atgttgttac cataattggt attcaagata ttgtttttgg

85921 agaggtagat agatagaatt actatttcac aggtaggacc ctaactttat cgagccaaaa

85981 aatatttttt tcgcgaaact caaaacgtcg ctgaatcatc tatgatgact catcaacata

86041 gcttgcggag aagtatatac atagcgaatt gctacggaat gcgctatttt aaggcttttc

86101 ttctccagag ctacgcactt ttttgttcgt tttgtgaaca aagagcacag aggcagaggg

86161 tgccttggcg cttttttccc tcatgctttt tcgataagta gagaaaataa gcttgcaaag

86221 gtcacggagc gcagtaaaaa aaatatatat atatatttca ttctgctgtc tgctctaccc

86281 ttggcatagt gaatgacagg gccgggtgga acgatgaaag cgcccgcggc ccatcaggat

86341 tatggcattc ctacgtaatg ccataaaaaa agcactaatt taatttcatt atggaacgac

86401 taactaaaat gcatcgttat taaatctttt gagaatgcaa gcctagagca cctacttgac

86461 acacacaaga ttgaatcgct tagaaaaaag atcttatata cagaaaacaa tctgaaataa

86521 gaatacatag aagaaggtta aaaaaaagcg cgagaagggc gcagcaactc tatgatctac

86581 tcgcagttca atccatctta ttataaggtg atagcaaacc ttgttgcagg cgatcaatca

86641 tcgtagatga gacattgata caaataaaaa aggcaaatac accatgacac taaaacaatt

86701 aacttttcgt ttaaaaaaaa agtctgctgg cagaaattca tcagggcgta ttaccgtttt

86761 tcatcgagga ggtggatcaa agcgattgca tcggaaaatt gattttcaac ggagcacttc

86821 gtctattggc cttgtacaaa gaatcgacta tgatcctaat cgttcttctt ggattgcttt

86881 agtacgatgg cttagagcaa tgaaacaagc ggaggcagcc aatagtcaaa cagaagaaaa

86941 cgccaagcgt tttcttggtc gaagagaaaa aaatcttttt tttttcggcc tcttattttc

87001 gttttcttct ttgtccagga aagcccagag aagaaattac gtttttttct ctgccctttt

87061 ttccctagag acaaagagag aggctgcaat tttaggctct ttcggtagct ttcttgattt

87121 acccaggata gccttagccg gagcaaagcc cgctttcttt gcttcgcgaa tgaaagactt

87181 cagaggacat aatacgtttt ataaaaatga aagcggaagg tggaaaacgc atagcgaagt

87241 gcaaagaatc gaacgcaaag cgctttcttg gagaactaat ctattttttt cttttaaacc

87301 taagcatagc gaagaagaac caatggttga agcgggcaaa gtagatcgtg cacctttcac

87361 ttatatatta gccagtgatc agttagaagc tggcaagacg gtaatgaatt gtgattggtc

87421 taaaccttcg acttcgttcg accaatataa atcttcccat aatttgctag cccataacga

87481 ccttcggttc caaaaccact ttgttcatac aacaaatgaa ggccaaaggt ccctcagggt

87541 ggaagagccc gtgcagcgta gtcaggctgc ttcttggcta cgccccgggg aggactacgc

87601 ttcaaatgaa aataaaaaca tacttgattc atattatcaa atggtaggaa attgcgtagc

87661 attggctaat atacctatag gcacatggat acataatatt gaatggaatc caggtcaagg

87721 cgcaaagttg attcgagctg cagggacctt tgctcaaata attaagaaat tcgaaaatac

87781 accacaatgt attgtgcgat taccttcggg tgttgacaaa ctcatagatt cccgatgccg

87841 agctactgtc ggtatagtgt ccaatcttca tcatggtaaa cgtaagcttg acaaagcggg

87901 acaaagccga tggttaggca gacgtcccat tgttcggggt gttgctatga atccggtgga

87961 ccatcctcat ggaggaggtg aaggacgcac aaaaggaggt agaccttcgg tatcaccttg

88021 gggaaagccc gccaaaggtg gatttagaac agtagtaaga aaacgcagaa attagtttat

88081 gacacgatct gtatggaaag gcccttttgt ggatgcttgc ttgttcaagc aaaaaaagat

88141 cagatggaaa atttggtcac gtagatcttg tattttgcct caatttgtcg gttgctatgc

88201 acaaatttat aacggaaaag gttctgttgg tttaaaaatt actgaagaaa tggttggtca

88261 taaatttgga gagtttgctt ctacacggaa accttcttcc tctgggaaga gagcttcccc

88321 cttgaaaaca aaaataaaac aaaaaaaaaa ggtacgatag tgaactatgg cgcaaaaaat

88381 aaatccgatt tcagtcagac tgaatctgaa tcgtagttca gattcaagtt ggtttagtga

88441 ttattattat ggaaaattgt tgtatcaaga tgtaaatttt agagattact ttaatttaat

88501 acgtccacct acgggaaaaa cgtttggctt tcgtctcggt aagtttatta ttcatcattt

88561 tcctaaaagg acattcattc acgtattttt tttggatcga cttggccgat caagacacac

88621 aggccttggg gcaatacaat cggtcaagct gattaggcat attgacgacg ctacaaagat

88681 acagcgaaac gaagtcaaga ttcgccgcta tggatacgat gataggttac catcaatgca

88741 cgaaatcgat caattacttc ggatcagcgg ttggatggcc tccaaaaact ctacttcttt

88801 gaggaacgat gcccttttgg agaatgatga cagaaaaatg tcagaaaaaa gctatgcgtt

88861 ttcttgtttt ggctctttgc gtcaaattag cgacgtattt ccacagacca ttttcgcagc

88921 tgtgcgtgct cctttaaatc atttggtcat gcaatacctc ttttattcaa agaaccgaat

88981 tcaatttgat cctattgtta acataatttc caatttggcg gcacggagca taattaaaaa

89041 atatattacg aaggaagcaa aaaagaaaga ggacagcttg aaaaaaagaa tgcgttctat

89101 tctgttaaat aaaagcattt gttcgaaaaa agaaggctta acctatatgg acaaaaccgc

89161 gcaaggctcc tatgtggaag ccttacgggg ttctacccac ttcatccgcc aagcgaatga

89221 agtgggcttt gcaagaaaaa atagacccga aatttctccg aacatccaaa cggcttattc

89281 agtttggctt ttctctaaag atattaattc cggaaggaca gagatgcgta gcgcggaaga

89341 gcttttagct ttgcgcacgg cgctccccag ctttgtccgg gcactaacgt tcccacacca

89401 aaatgccctc cactgcttgc gcaaacagag ccttttaagg cttcgttttc aaatccatcg

89461 cgagcaaggc atgccattgg ctaataatta cgtaataaaa aacacagagc cgattcgtca

89521 acccgggtct atattggatt ttggtgctcc ctttatttca agagatgctg aatggaagaa

89581 agttcactct ttgttcagtc gttattatta ttggaaaaaa atgcaatttt ttttatctaa

89641 tcaaacaaaa actaatacct taattaggcc agtcaaaata gcttctgttt atcaaagtgc

89701 ttctctaatt gctcaagaaa tatcttggaa actagaacaa aaaaaatcat ttcgacaaat

89761 ttgtagatct acatttcaag agattgaaaa atgtcaatat gttaaaggaa tccgtatttg

89821 ttgttcaggc cgattaaatg gcgcagaaat agctaaaact gaatgcagaa agtacggtga

89881 aacctcttta catgtatttt ccaatcaaat cgattatgcg aaagcacaag catctactcc

89941 ttatgggatt ttaggtgtca aagtgtgggt ttcatatttt taacacaaaa aaagggacaa

90001 gttgtgctat atccaaaacg tacaaaattt cgtaaatatc aaaaaggcag atttaagggt

90061 tgcaaagcag acggtacaca actttgtttc ggaagatatg gcatgaaaag ttgtgaagct

90121 ggtcgtatct catatcaagc aattgaagca gcgcgtcgtg ctataagcag agaatttcga

90181 agaaatggtc aaatatgggt aagagttttc gcagatattc ctattactag taaacccacc

90241 gaagtcagaa tgggaaaagg aaaagggaat tctacaggtt ggattgctcg tgtggtagag

90301 ggacaaatct tatttgaaat ggatggtgtg agtttgtcaa atgcgcaaca agctgccaca

90361 ttagcagcgc ataaactatg tttgtcaacc aagtttgttc aatggtttta attggttaat

90421 ggataaaaag cgaggccgaa aggcgcagag caaagcaaag aaagtgggta aagaccagga

90481 atctttgtaa acttatggcc tctatcagcc taaaaacgaa caagcaatca agacgataga

90541 agtgttgaaa ccgtaaagcg agtaaaaaat ttattattat aaataattca tggtctttga

90601 ccccaataga tatagcccaa aggttagaaa aaaagcctaa aaaaataaag aaatatctat

90661 cttgtgttcg ctctttgctg cgccttttgg aatggaggaa cggcgcgact tacaatttat

90721 ttgtaatacg aataaagtaa ttttagcaga ataaaatgcc ttgaggccga aggcctcgag

90781 tccaagacga ttattttgtt cgcagccttc taggtgaacc atgtaataga ttaaattgcg

90841 tagcgcagtt ttgtcccaca cagcactttt cttgtttttg aatataataa agcgcatggc

90901 gttgaggtcg aagacccccg agtgaagcgc taaggcttcc gggctaaaat atttgctgcg

90961 aaaagtgaaa aaccactttt ttcgctttct tggggcgcga agctaatcaa gagcttgcta

91021 ctacgtcctc ttacgctctt ccttttatta cggaaaagga aggcataaca gcccgctatt

91081 taaaaatcag ataggggaca gtgcttatat tcgtttttac ctgaaataaa aaaaaaagca

91141 gccaacttat gttcactcca aatagactcc gttttcatta cgaaaattta ttacgtcaag

91201 atttgttgtt aaaattgaat tatggcaaca ttatggaagt tcctagattg tgtaaaataa

91261 taatagttcc aaaagcaccc tctaatttga taaaaaatgt aaaattagct atggagattg

91321 tttgtggtca aaaattcata cggacacgaa gcagagattc ggcaggaaag tcgtttcgat

91381 ttaataaatt tatattgaat caagagtcga aaaaagacac aggatatatc acttacctag

91441 cacaaagcac tctacgaggg cataccatgt ataatttctt ggaaaagctt attacgataa

91501 tatcttttta cgattacccg gttaaagtac aaaaaagctc cattcaatta gcaatgccaa

91561 cgccgttgtt acgattattt ccggaaatac aaaatcattt cgagattttt gaacatattc

91621 aagggtttga tgtaactatt gttacttcag ccaaaacaca agatgaggct ttcattttgt

91681 ggagtggttt tttgcaaaaa gaggtttagt catatgtcaa atcaaattat acgagatcat

91741 acacgtagat tacttgtggc taaatatgaa ttagagcgaa tgcaatgtaa agctatttct

91801 cgacataaaa acctccctaa tcaaatacgt tatgaatatt ttttaaagtc gtctaagttg

91861 ccaagaaata gttccaaaac acgagtaaga aaccgatgta ttttcacagg tcgccctcgt

91921 tccgtatata agttatttcg cgtttctcgt atagtttctc gtgaattagc atctaaaggt

91981 tctttaatag gcataaacaa atcgtgttgg tagtcaatgg aataaaggtt agctttgcga

92041 gtatccatag ggtgcagcaa aaaaaactat tttttgcttg aaatagtttt tttttgcttt

92101 acgttttttg gttttattcc ctacagcgct gtgcgctctt tggctcctga atactgaacg

92161 aactcgaccg gtgtgccgcg ctatgtatgc gccttaaacc caagacgtac ttgctggact

92221 ttgttaccat aaggttgcaa catgccccac tcctaatgaa ttagcagtct taaggcggtt

92281 atactggaat gcgcgaaggc ccctcctaag ctaaaccacg taaaaaatta tgtagaggac

92341 ggttgccccg atcttcctaa ctgaagttct aaaattgcca ttataacaag aagaatgcgg

92401 tcctgtttcc aaagagatca ggaccggaat gataaataaa aagcagggtc ctttgaaatg

92461 aaggatattg aaaccacggg cttcgcccta agttcaagaa aatctcgccg ggcgtaaacc

92521 attattgtat cacaatccta cctaaccatg tggctactct ctgcgtacta aggtgcgcga

92581 gttgatggcg tggaacaact ccattactag aattctacaa gattttgatt aggttacgcg

92641 caaatctcat ccgctgcaat aagcaggcaa ggaactcatg tttcgtgcag catgcttaaa

92701 tgagcgtaca agaaaatatc tatccgcccg cgtttactaa tagaatatat tctaccgcct

92761 ccgtcacctt aatgagcttt tttaacataa gaacttttaa acgcgtaatg gtcaaatccc

92821 atgaaataag agttcaacct atacccaagc gaaggccaga gttcttatta gaagcgccgc

92881 aaaatactta taaatagaat atctccatag tctaccgtag gtgtgattac tggcgtgtag

92941 tctataaaga gagtgactat acaataggta actggtgaat ctgcacctta atcaaaaggt

93001 cgcggctggg atgcttttaa catctaacct ttgcatgcca cttgccatca gcaaatcacg

93061 cgaagtaata ctagatttgc gaatgaatca aaaatgccct gaaagcgccc gcggacgccc

93121 gacgatagca cttctgataa tcaacttgag ctgtatgaag cggaagcttt cacgtatagt

93181 tctgaggact tacctatttt gattattcaa aaagagaaac aagagaataa aacgcttgct

93241 tttgtcagca tcaaggtgtc tccggtgttt tcgttttgcg tctctatcgg gattttttta

93301 tgattttgta caatgcaagt tttctattaa cagattctgt agggaagcaa acagaaggtt

93361 gagaatcgtt ttaaaatgtc atttttttta ttaaatttat tctaaaaaaa tgaaaaaaat

93421 ctctgaaccg aagtctctaa tttcttcatc aacaactcgt ttagggctta gtataataaa

93481 gaatcttatc cacttcaaag tgggctttga tatgtgatcg tgaagtacga aaaaccgatg

93541 gcgagattct atgccaggtc tataaaaaaa aattaagtca agtttatgga agccaaattc

93601 ttttgttttt tggaaataat tggagttggg tacaaagcca gtactaatcc acaaggctct

93661 attttatatc caaaattagg ctttagtcat gagattcgac ttcaagtcac gtccgcagtt

93721 cgcgttcttt gcttcaagcc taatatcatt tgttgtactg gaatagatca tcaaaaagta

93781 actcaatttg ctgccagcat caaaagttgt aaacctcctg aggtttataa aggaaaaggt

93841 atacaatatc gaaacgaaat tctacgtaaa aaacaaggaa aaaaaaaata aatcatgtca

93901 tatattttag gaactaattt aatgccgaat gaacaagtag aaattgcttt aactcgaatc

93961 tttggacttg gccctaaaaa agcaattcaa gtgtgtgctc aattaggttt taatgataat

94021 attaaagtta ataagttaac taagtatcaa attgatcgaa ttatcaaaat aataagtcaa

94081 aattatttag ttgatttaga attagcaaga gtaattcaga gggatattaa acaattgatg

94141 agtattggtt gttatcgcgg ttttcgccat aatgcgggat tgcccttacg tggtcaacga

94201 actcatacta atgctaagac ttgtcgcaaa ttcagaaacg tttcgatcaa tcaacgaagt

94261 tgattcaggc cgcaggctgt aagttttttt tattatccat aataaatgat gaaggcgcga

94321 agcgatgtgt aatgaaattg aaatttcatt acacattttg ttattggctt ttcctccata

94381 aaaacatcat cgattggtaa ggccggctcc tattggttgg catataggcg caacaagtca

94441 aagggcgcag taaatctata tatatatatt ttttttttaa gttatcgcat attttttatc

94501 tattctcgca ccgtaactgc ctttcggcag ggcgggctcg gataatagaa tatctcacct

94561 aaaggaccaa aagctgcggc gttctctttt gttcaatgga ttattttgta caaatttttt

94621 ccacaaaatt tatttttaat tagtaaacag ataagatgga ttgtaaaaaa acccgatcga

94681 tgatatcaaa caaaatctaa acattcaaaa aaaactcatg cagaagaaaa aaaagcacgg

94741 tattgcatat attcgatcaa ctttaagtaa taccattatt actgtaacag atcataaagg

94801 agatacaaaa acttggtcct cctcaggttc attggggttc aagggatctc gtcgctcaac

94861 caattatgct gctcaagcaa ctgcagaaaa tgctgcccga actgctattc aactgggaat

94921 taagtcggtt gaagttgaaa taaaagggtt aggttatgga aaggaatcgt cgctacgtgg

94981 tttacgacta ggaggtctta ttattaccga aattagagat gtaaccccaa ccccacataa

95041 tggatgccga ccccctaaaa aacggcgtgt ttaaatatca tcataaagtt attcattttc

95101 aattacttga taatgcaata tagtaaaatc ccggggtaca ggcccggttt caccattttc

95161 taatgctaat gtaggaagcc ctcgttagca tttaacagcg agtttatcac atctggggag

95221 ataacaacaa gtgccaatcc tttccagtct tattataaaa accaaccaag tttgtgggta

95281 aagatacttt ctttctataa aaaacaacaa taactaactt tggattaatt tattacacgg

95341 attttctttt ttaaggaaag aaggatcgga taaactcgaa agcgaaccca gggctatttt

95401 acttgtccta taagagatta cataaacgtg ataaaaggcc gaaaaaaaaa tatatttttg

95461 ctacgcccgc aattacatag taattgcatt cctcatgaaa ctgagtatga ggcgcaactc

95521 tcagctatac gactccagtc tcttctggct cgctccatta gtcgagattg tgtaaataga

95581 aaacgtcttt acgcctttat ttgtgtttta accacatgaa atgaatatga catcacttgg

95641 ctaaatggtt gggttggcct catttcgatt gatcagccct ggaatggtca ttgttttgac

95701 agaaacaaaa attaaattgt tatgctagaa ggtgcaaaat tagtgcgacc cgttggccca

95761 caaacctaaa aatacttaaa aaaaaatata tatttttttt tttgccggaa cttagtattc

95821 taactcttgt cggatgcagg tgtaatccct gtcgcgcggt aatgtcccgc aaactctcga

95881 tcattacatg ggagtggcaa ccccggaatt catagcagaa tggtcgcagg aagaaaaccg

95941 agaggaacac tttggttaac gagttaaagc ttcggtgccc gatcaccttc ggtatgctga

96001 acccttactg ggggctagac cacaagtcaa tgaggacgag tatgattagc ttgatttcta

96061 atcataggca ttctggggat acagtaaaag aggccaggaa agtagttgct ttcactacgt

96121 tctacgtgcg tgttccacct cccgcagtat tgctcgtctc ttaggttttc gcaacaatcc

96181 gactcgggaa cggggtaact accttgaact ccaaacaaat gatcgtaggg taggctagcc

96241 aggccacatg ttcattggta gcaggattct ccaaaaagcg aaagcgctgt gataaattat

96301 tgtgatatgc ccacttggag actcataact ttaaaaaaaa ctgggtgttc cgggtaaaaa

96361 atatatatat tttttttaag tgatattttt cgataaaaaa tctatttttt ttacctgcgg

96421 cctggacacg ctatgccccg gccaaaggcc gaagggcttg tgttcagatt agaatccagg

96481 atctataagg cttcgcggca gaataaccat ggaaaacaaa acgcaggcac tccaaaagag

96541 gctaacggct ggaacacagg ccatattgat aggtatgttg aacaaaggca gactgtaaac

96601 aaggtagaca atactcacca ccaaagagcc aagattgaag atggcgcgaa cattgcagtt

96661 gaataaaggt gcacaatccg tgcatcgcat cctttcgact tttgttcagt gatcaaataa

96721 aaaaaatctt tttttattct tggttaagct atttcataag acatgcactt ggttcgccac

96781 ccgaacccag ttgcagaaaa aaaaagtaaa aaaaaatata tctatttttt tggtaaccat

96841 tcgatggtga tacaatgtac ttaattagat gtaagaccgc caaagctttc tgagtcgaca

96901 cgagcttagg cttttgaagg tgaaaggaag gatatggatg tcagggttct tttagaagtg

96961 gatcatatta ctcctaagaa aagcggaagg caagcaggac atttatgcaa acttttaatt

97021 gccaaaattt tcttttacat ggtcaaaact ctcgaagata caaacattat gagaagagcc

97081 gtatgaggcc gtaggctcac gtacggttcg gaagccgagc tgcgtcagca atagagtggc

97141 ttaggttaac attggagcag gagcagctac cattgcttca gcgggagctg ctataggtat

97201 tggaaacgta tttagtgtgc gcctcgctag agcgcgtttg gatcagcgaa ggttaataga

97261 ttatcgcctg ggcggtcccc taacccgtag cggaaacaga ccgcagggaa ccatagcatg

97321 gggcctggtc aagtttcgtg gcacggttat cacgagtgcg tcagggtcaa gcgttacccc

97381 ttccaacaaa ggtggcccgg aaggctccaa ggcccaacta aattcttggt gcgaacaacc

97441 ctccggggga aactgcaaga agcatgaaaa accgctcact aacctaaacc acgagcaggc

97501 acccaaacgt gaaagcgagg gatcacccca atggaccctt taaggttaac acttgggtac

97561 atgccagcca aagagccgag gtatgaacta aaaagaaatg ggtgacagat cagccataag

97621 tactccaaag gatacactgg gcaaggcaag tcgtgcatgc gacaatgccc gtaacgtgaa

97681 aaattttgag gaaagggctg taaatggtaa tgtgctaccc cttacagacg cgggcatgcc

97741 cgcgtctgta aggggtagcg aaaatcagcg aaagcaacta ctaaaactaa cgccaataaa

97801 aggcgcggca gactggtggc gcctcctgcg cttcaacctt ggccagatag catagcctac

97861 agccggccga ggctgctttc tacaaatttg gttcgaacct gcaaatcacg gaagaagggc

97921 agaaggtagc gtcactatac tactcacttc tccaaaaaat ataaaaaagt ttcacattca

97981 aaacccccgc tttacttagt gagataacta cactcgaaga accttattat agagtaaagc

98041 gtggttacag ctgggcagat tactcacaat acacggatga atccgacttg tgcggtaaca

98101 caaaccagct tgcactacat cacttaagac ccaaagacca aaggactctt gttaaagtag

98161 ccatagccaa atctaaaaag tagatcacac tatgctgcaa atgccatagt tatgaggtgc

98221 acgcggaaga ggagggatgg aatcgcataa tatgtgtccc tgtgcagaga ggactagtag

98281 tgcttatacg gcaagaagcc gcaaaagctg aaaaatttta ccatggacga gccacatgcg

98341 aagaaacttg cacgtgtggt tctgaccggg gggggggaaa gcaccctatc ggaattcttc

98401 gatgtgcgga gcttcgcatc tgaaacccga tgacgctttg cgttctgccg gggccttggg

98461 cagtaatcca actcccgcca actcacgaga agctggcaat gccgcggagt tagggaagtg

98521 gcttgtttaa gtgtaggcgg acgaatctcg acaatagccg ctcttataaa ctagggggga

98581 ttattctcat cacaggttgc cgcccttact taagtatact gagaatcgac agtgaaaggg

98641 agcccctagg ggaggggaat gaacgacctg tggtcgccga ctaacgtcgg ttaagcttag

98701 aaagcactga acaggctgga cgggtcgaca aagatgacag ctacaaggat ggtaatcctg

98761 gtgacagaga tatacattcg gggatgaata gaaaacctcc cacagaaaag gccgcatttt

98821 tctctggcga ggaatgtagt tctggctttt taccaggaaa aagcgaaaaa aatactgttt

98881 tttgtgctgc ttgcctggca aaattattca atcttacgcg tgaacctaat gggaagtatg

98941 agaacctgat ggagataata tcggacttaa acgtactgat agctgcttaa gataagctga

99001 aatctaactc ggataagggg atggggtgtt atgacacaat cgaccggcac cgcttagtct

99061 tcattctctc gtaagaaatc cggtttattg agtggatatt gaagttattg caaggcaaag

99121 gccacaggta ggtcgcaggt atatcttttt tttttattaa tctagtgaga gaccccgcct

99181 gaagcggaag cctcacaagg ttgtgccata tcactcatac ttcgcaatat ttacccgaat

99241 aaatcggcgc tgaaagatcc aacatgactc cggacctccc aaaccccgaa tccagagtat

99301 aaaaaagcta tcgctatatc gaaagcaaag acacgagctc atggcgaaaa caacaaaatg

99361 gtgctgcccg gactaaaagc tcatgaaact gaaaaagttt aagaaggggc gcagcaatat

99421 atatgttgcg cccttgctgc cgcgttcttc tctggccaca cttgccaagc gtataagggt

99481 cttcagggcg caaatatttt ctgagaaaac aagtagagaa atgacgtaag atatgaaaaa

99541 agaggagccg tatgatgggc aactatcacg tacggttcta tcaggggggg gagttgcgca

99601 tcatgggtac cttcttattg ccgtgaaggg cgatatgaaa ataaccccct atccgacctc

99661 attctgttgc gcgaaatcca tcattggcta agcaattatt tggttatgct attttaggtt

99721 ccgcttcaac agaagctatt gctttgtttg cctcaatgat ggcctttttg atcttattcg

99781 tctttcaatt tttcggtgct gggattttct gggaaaaaaa atatattttt gctgcgccct

99841 attggctttg cgaatagggc tgcgcccaaa aaatctagat ttttggcacc ttagggccga

99901 acgattcgta cgttcgagcc cttcaccact tgctaccaaa agcataagcg taaaagaact

99961 gaactgacaa agattttaac ctttacaatt gctttgggag cagagcccta aaggctcatt

100021 ggataaaaaa agaagcagga caaatatata tatatatata tatatttttt ttctttttta

100081 gctgctttac tttttgttag aaataatctt taataatctt tgataatgca tagcttcgcg

100141 tgcttttcga ttgatgatga gaccggagac caaaggcgcg aagcggccgc gggtctagat

100201 tttttttttt tcggccgcgc gcctgcggcg gccctcggtc ttcgttaact caccaaatgc

100261 ttccgcgtta ttctacgcca ctaacctttg agcggcttct ttcgtttgat tcccggcctc

100321 ctatacctat agttaacgag gg

//
