# Supplementary figures and images for: Complete mitochondrial genome sequence of the “copper moss” Mielichhoferia elongata reveals independent nad7 gene functionality loss
Source: PeerJ. 2018 Feb 2;6:e4350. doi: 10.7717/peerj.4350 (PMC5798402; doi:10.7717/peerj.4350)

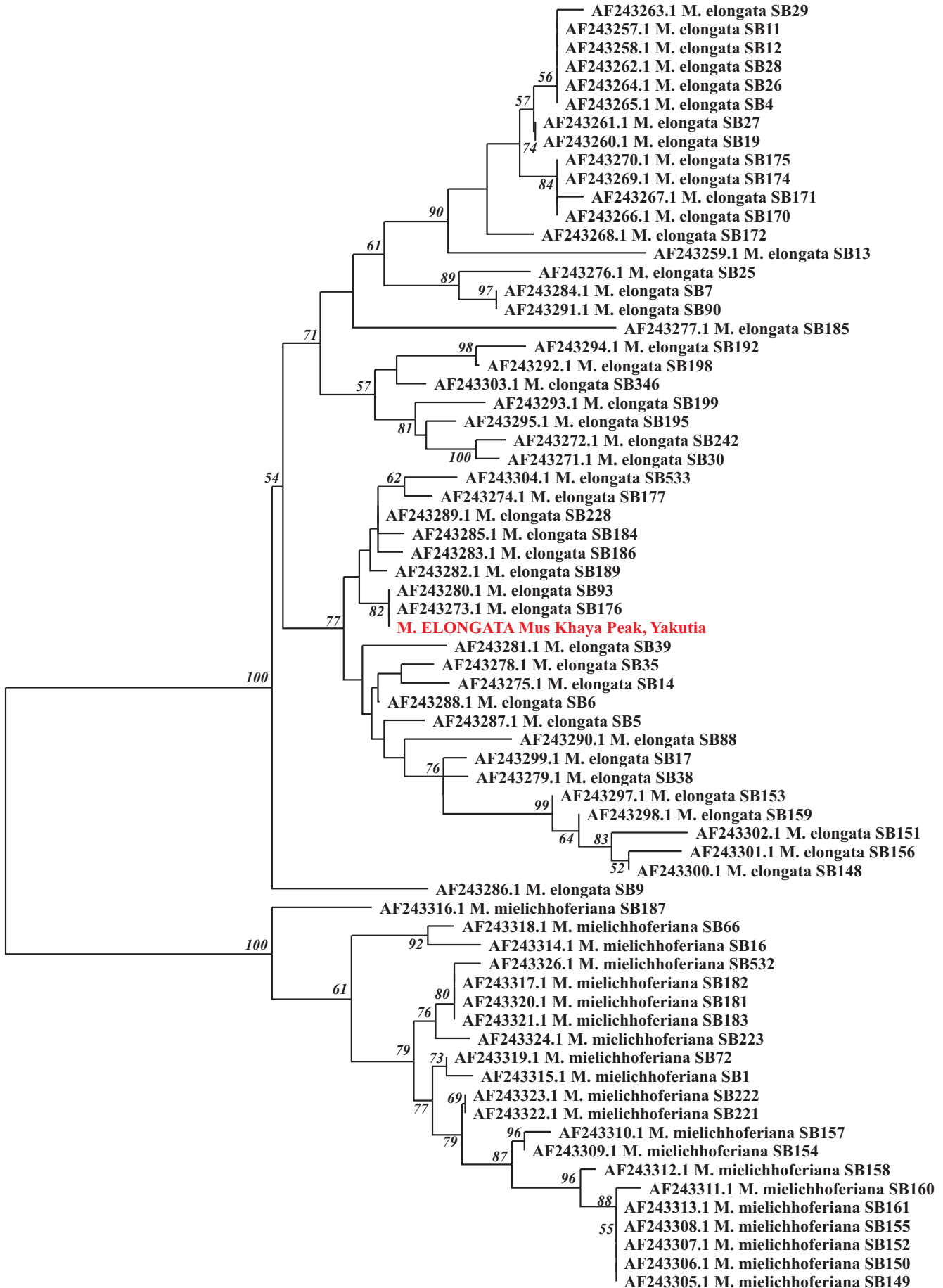

Supplement: Supplemental Information 2 — The phylogenetic tree based on nuclear rDNA region (5.8S rDNA–ITS 2–5‘-end of 26S rDNA). The alignment consists of 440 positions. The tree reconstruction was conducted in TREECON software (Van de Peer & De Wachter, 1994) using the Neighbor-Joining method (Saitou & Nei, 1987) with 500 bootstrap replications. Bootstrap support values >50% are shown next to the branches. The evolutionary distances were computed using the Kimura method (1980) with gaps taken into account as it implemented in the TREECON package. [file peerj-06-4350-s002.pdf]
