# Supplementary material for: Complete mitochondrial genome sequence of the “copper moss” Mielichhoferia elongata reveals independent nad7 gene functionality loss
Source: PeerJ. 2018 Feb 2;6:e4350. doi: 10.7717/peerj.4350 (PMC5798402; doi:10.7717/peerj.4350)
Supplement: Supplemental Information 3 [file peerj-06-4350-s003.pdf]

```
         10        20        30        40        50        60        70        80
....|....|....|....|....|....|....|....|....|....|....|....|....|....|....|....|
ATGGCTAAAACCAAACAAATCAAAAATTTTACTTTGAATTTTGGACCTCAACATCCTGCTGCTCATGGTGTTTTACGATT NC_027974_Sanionia
ATGGCTAAAACCAAACAAATCAAAAATTTTACTTTGAATTTTGGACCTCAACATCCTGCTGCTCATGGTGTTTTACGATT NC_024515_Climacium
ATGGCTAAAACCAAACAAATCAAAAATTTTACTTTGAATTTTGGACCTCAACATCCTGCTGCTCATGGTGTTTTACGATT NC_031212_Brachythecium
ATGGCTAAAACCAAACAAATCAAGAATTTTACTTTGAATTTTGGACCTCAACATCCTGCTGCTCATGGTGTTTTACGATT NC_024520_Atrichum
ATGGCTAGAACCAAACAAATCAAGAATTTTACTTTAAATTTTGGACCTCAACATCCTGCTGCTCATGGTGTTTTACGATT NC_024521_Sphagnum
ATGGCTAAAACCAAACAAATCAAAAATTTTACTTTGAATTTTGGACCTCAACATCCTGCTGCTCATGGTGTTTTACGATT NC_024516_Hypnum
ATAGCTAAAAGCAAACAAATCAAGAATTTTACTTTGAATTTTGGACCTCAACATCCTACTGCTCATGGTGTTTCACGATT KC 784953 Tetraphis
ATGGCTAAAACCAAACAAATCAAAAATTTTACTTTGAATTTTGGACCTCAACATCCTGCTGCTCATGGTGTTTTACGATT MF 417767 Mielichhoferia
-------------------TCAATAATTTTACTCTGAATCTTGAACCTCAACATCCTGCTGCTCATAGGGTTTTACGATT NC 024518_Buxbaumia

                                                              Intron 1
         90       100       110       120       130       140       150       160
....|....|....|....|....|....|....|....|....|....|....|....|....|....|....|....|
AGTATTGGAAATGAATGGAGAAGTTGTAGAACGCGCGGAACCGCATATTGGATTACTTCA--GTGCGGCATGAAGCCGCT NC_027974_Sanionia
AGTATTGGAAATGAATGGAGAAGTTGTAGAACGCGCGGAACCGCATATTGGATTACTTCA--GTGCGGCATGAAGCCGCT NC_024515_Climacium
AGTATTGGAGATGAATGGAGAAGTTGTAGAACGCGCGGAACCGCATATTGGATTACTTCA--GTGCGGCATGAAGCCGCT NC_031212_Brachythecium
AATATTAGAAATGAATGGAGAAGTTGTAGAACGCGCGGAACCGCATATTGGATTACTTCA--GTGCGGCACGAAGCCGCT NC_024520_Atrichum
AGTATTAGAAATGAATGGGGAAGTTGTAGAACGTGCAGAACCGCATATCGGATTACTTCA--GTGCGACACGAAGCCGCT NC_024521_Sphagnum
AGTATTGGAAATGAATGGAGAAGTTGTAGAACGCGCGGAACCGCATATTGGATTACTTCA--GTGCGGCATGAAGCCGCT NC_024516_Hypnum
AGTATCGGAA-----TGGAAAAGTTGTAGAACGCGCGGAACCGCATATTGGATCACTTCA--GTGCGGCACGAAGCCGCT KC 784953 Tetraphis
AGTATTAGAAATGAATGGAGAAGTTGTAGAACGCGCGGAACCGCATATTGGATTACTTCA--GTGCGGCATGAAGCCGCT MF 417767 Mielichhoferia
AGTATTAGAAATGAATGTGGAAGTTGTAGAACGCGTGGAATC----ATTGGATTACTTCA--GTGTGGCACGAAGCCGCT NC 024518_Buxbaumia

        170       180       190       200       210       220       230       240
....|....|....|....|....|....|....|....|....|....|....|....|....|....|....|....|
GACGCCGAGTCGGCA-TATCC---TATGCCGCTAGCTATGTCCTGCTTGTTCCCCACCACGGGT-GGCGCGTGGAGGCAA NC_027974_Sanionia
GACGCCGAGTCGGCA-TATCC---TATGCCGCTAGCTATGTCCTGCTCGTTCCCCACCACGGGT-GGCGCGTGGAGGCAA NC_024515_Climacium
GACGCCGAGTCGGCA-TATCC---TATGCCGCTAGCTATGTCCTGCTTGTTCCCCACCACGGGT-GGCGCGTGGAGGCAA NC_031212_Brachythecium
GACGCCGAGTCGGCA-TATCC---TATGCCGCTAGCTATGTCCTGCTTGTTCCCCAGCACGGGT-GGCGCGTGAAGGCAA NC_024520_Atrichum
GACGCCGAGTCGGCG-TATCC---TATGCCGCTAGCTATGTCCTGCTTGTTCCCTGGCACGGGT-GGCGTGTGGAGGCAA NC_024521_Sphagnum
GACGCCGAGTCGGCA-TATCC---TATGCCGCTAGCTATGTCCTGCTCGTTCCCCACCACGGGT-GGCGCGTGGAGGCAA NC_024516_Hypnum
GACGCCGAGTCGGCA-TATCC---TATGCCGCTAGCCATGTCCTGCTTGTTTCCCAGCACGAGT-GGCGCGTGGAGGCAA KC 784953 Tetraphis
AACGCTGAGTCCCCACTGTGG---TTGACTTACCACTGGGCTTT-CTTGGAAACGAGAACGCGTCGGCGGGTCCTGG--A MF 417767 Mielichhoferia
GATGCCGAGTCGGCA-TATCTTATTATGCCGCTAGCTATGTTTTGCTTGTCTCGCA-------T-GACGCCCAAAGGC-- NC 024518_Buxbaumia

        250       260       270       280       290       300       310       320
....|....|....|....|....|....|....|....|....|....|....|....|....|....|....|....|
CTCCCGCGTCATAAGCACCGGGCAAAA-------------------------------GGCGTTTTGTTGGGCAACTCAA NC_027974_Sanionia
CTCCCGCGTCATAAGCACCGGGCAAAA-------------------------------GGCGTTTTGTTGGGCAACTCAA NC_024515_Climacium
CTCCCGCGTCATAAGCACCGGGCAAAA-------------------------------GGCGTTTTGTTGGGCAACTCAA NC_031212_Brachythecium
CTTCCGCGTCATAAGCACCGGGCAAAA-------------------------------GGCGCTTTGTCAGGCAACTCAA NC_024520_Atrichum
CTTTCGCGTCATAAGCACCGGGCAAAA-------------------------------GGCAGTTTGTCGGGCAACTCAA NC_024521_Sphagnum
CTCCCGCGTCATAAGCACCGGGCAAAA-------------------------------GGCGTTTTGTTGGGCAACTCAA NC_024516_Hypnum
CTTCCGCGTCATAAGCACCAGGCAAAAATAAAAGGAAGAGTTTTTTCTTTTTATAGGCGGCGTTTTGTCGGGCAACTCAA KC 784953 Tetraphis
GTACC-CGTCAGGGGCGC-ACGCGTATTCCCGCGGGGTGATTATTACGA---------CCAGCCCC--TCCGCATCTTAG MF 417767 Mielichhoferia
-TT----------GGCA-CGGGCAAAA-------------------------------CCCGCTTTGTCGGGCA--TAAG NC 024518_Buxbaumia

        330       340       350       360       370       380       390       400
....|....|....|....|....|....|....|....|....|....|....|....|....|....|....|....|
GTGAGGCAAATCGCTCAGGGGAAAGGGGGGAGAAGGTCGTGAAGGTGGCCTCGTTATCCACACTAGAGGTCTCGACAGAG NC_027974_Sanionia
GTGAGGCAAATCGCTCAGGGGAAAGGGGGGAGAAGGTCGTGAAGGTGGCCTCGTTATCCACACTAGAGGTCTCAACAGAG NC_024515_Climacium
GTGAGGCAAATCGCTCAGGGGAAAGGGGGGAGAAGGTCGTGAAGGTGGCCTCGTTATCCACACTAGAGGTCTCGACAGAG NC_031212_Brachythecium
GTGAGGCGAATCGTCTAGGGGAAAGGGGGGAGAAGGTTGTGAAGGTGGCCTCGTTATCCACACT-GGGGTCTCGATGGAG NC_024520_Atrichum
GTGAGGCAAATTGCCTAGGGGAAAGAAGGAGGAAGGTTGTGAAGGTGGCCTCGTTATCCACACTGGAGGTTTCAACAGAG NC_024521_Sphagnum
GTGAGGCAAATCGCTCAGGGGAAAGGGGGGAGAAGGTCGTGAAGGTGGCCTCGTTATCCACACTAGAGGTCTCGACAGAG NC_024516_Hypnum
GTGAGGCGAATCGTCCGGGGGAAAGGAGGGAGAAGGTCGTGAAGGTGGCCTCGTTATCCACACTGGAGAGATGAGTAAA- KC 784953 Tetraphis
CACAG------------------------CGGAACGT-GTAA--CCGGCCTGAG-GTCC-CATTTCAACCGCCAATTTAT MF 417767 Mielichhoferia
GCGAATAAATCGCCAAAACAGAAAGAAGGGAGAAGATCGTGAAAGGTGCTCTCTTATCCA------GGATCTCGATAGAG NC 024518_Buxbaumia

        410       420       430       440       450       460       470       480
....|....|....|....|....|....|....|....|....|....|....|....|....|....|....|....|
ATGAATAAGGGTATGTCA--ATACCACCACTGTGGTTGACTC--------ACCACTGGGCTTTCTTGGAAACGAGAACGC NC_027974_Sanionia
ATGAATAGGGGTATGTCA--ATACCACCACTGTGGTTGACTC--------ACCACTGGGCTTTCTTGGAAACGAGAACGC NC_024515_Climacium
ATGAATAGGGGTATGTCA--ATACCACCACTGTGGTTGACTT--------ACCACTGGGCTTTCTTGGAGACGAGAACGC NC_031212_Brachythecium
ATGAGTGGGGGGACTTGG--CGCCCCCCACTGTGGTTGACTTACAAGTCGACCACTGGGCTTTCTTGAAAACGAGAACGC NC_024520_Atrichum
ATGAATAAGGGGACTTCA--GT-CCTCTACTGTGGTTGACTCAGAAGCCAACTATTGGGGTTTCTCAGAAACGAGAACGC NC_024521_Sphagnum
ATGAATAGGGGTATGTCA--ATACCACCACTGTGGTTGACTT--------ACCACTGGGCTTTCTTGGAAACGAGAACGC NC_024516_Hypnum
TTAAATGGGCGGATGGGGACGGGGACTTGGCGTCCCTCTCACGAGCCGATATAGTCGGGCTTTCTTGGAAACAAGGACGC KC 784953 Tetraphis
TGTCCT-----TACAAT---GGGTAGGCTAACCACAAGGTACAAGCC---AAAACCTTAGGTCGAATAGGACG-GCACTA MF 417767 Mielichhoferia
ATGAGCAGGGG--------------------------------------------------------------------- NC 024518_Buxbaumia
```

```
        490       500       510       520       530       540       550       560
....|....|....|....|....|....|....|....|....|....|....|....|....|....|....|....|
GTCGGCGGGTCCTGGAGTGCCC-GTCAAGGGCGCACGCGTATTCCTACGGGGTGATTATCACGACCAG-CCCCTCCGCAT NC_027974_Sanionia
GTCGGCGGGTCCTGGAGTGCCC-GTCAAGGGCGCACGCGTATTCCTACGGGGTGATTATCACGACCAG-CCCCTCCGCAT NC_024515_Climacium
GTTGGCGGGTCCTGGAGTGCCC-GTCAAGGGCGCACGCGTATTCCTACGGGGTGATTATCACGACCAG-CCCCTCCGCAT NC_031212_Brachythecium
GTCGGCGGGTCCTGAAGTGCCC-GTCAAGGGCGCACGCGTATTCCCGCGGGGTGATTATCATGACCAG-CCCCTCCGCAT NC_024520_Atrichum
GTCGGCGGGTCTTGGAGTGCTC-GTCAAGGATGCACGCGTATTCCTCCGGGGTGATTATCACGACCAGCCTTCTTCGCAT NC_024521_Sphagnum
GTCGGCGGGTCCTGGAGTGCCC-GTCAAGGGCGCACGCGTATTCCTACGGGGTGATTATCACGACCAG-CCCCTCCGCAT NC_024516_Hypnum
GTCGGCGGGTCCTGGAGTGCCC-GTCAGGGGCGCACGCGTACTCCCGCGGGGTGATTATCACGACCAG-CCCTTCTGCAT KC 784953 Tetraphis
CT-GGCAAATACTATCTAGCAAAGACACGAATCATGAAGGATAGAGCTTGCGTCAAAAGCATACGGGA-AAGTTCTAGCA MF 417767 Mielichhoferia
-------------------------------------------------------------------------------- NC 024518_Buxbaumia

        570       580       590       600       610       620       630       640
....|....|....|....|....|....|....|....|....|....|....|....|....|....|....|....|
CTCGGCACAGCGGAACGTGTAACCGGCCTAAGGTCCCATTT-CAACCGCCAATTTATTGTCCTTACAATGGGTAGGCTAA NC_027974_Sanionia
CTCGGCACAGCGGAACGTGTAACCGGCCTAAGGTCCCATTT-CAACCGCCAATTTATTGTCCTTACAATGGGTAGGCTAA NC_024515_Climacium
CTCGGCACAGCGGAACGTGTAACCGGCCTAAGGTCCCATTT-CAACCGCCAATTTATTGTCCTCACAATGGGTGGGCTAA NC_031212_Brachythecium
CTCGGCACAGCGGAACGTGTAACCGGCCCGGGGTCCCATTT-CAATTGCCAATTTTTTGTTCCCGGAATGGGTAGGCTAA NC_024520_Atrichum
CTCGGCATAGCGGAACGTGTAACCGGCCTGAGGTCTCATTT-CAAC-TTTAATTTGTT---CTTGAAATGGGTAGGCTAA NC_024521_Sphagnum
CTCGGCACAGCGGAACGTGTAACCGGCCTAAGGTCCCATTT-CAACCGCCAATTTATTGTCCTTACAATGGGTAGGCTAA NC_024516_Hypnum
CTCGGAACAGCGGAACGTGTAACCGGCCTGGGGTCCCATCT-CA-----CAATCTGTTGTTCTTGGAATGGGTGGGCTAA KC 784953 Tetraphis
ACGGGGAAACCGGAGGGAGAAACCAGTAGAGCCGCAAGAGCATAACCGGAAGGTTAA-G-CCAGGGA--GGCTGGG-TCA MF 417767 Mielichhoferia
----------------------------------------------------CTTGGCGTCC------------------ NC 024518_Buxbaumia

        650       660       670       680       690       700       710       720
....|....|....|....|....|....|....|....|....|....|....|....|....|....|....|....|
CCACAAGG--TACGAGCCAGAACCTTATA-----GGTCGGGTAGGACGGCACTACTGGCAAATACTATCTAGCAAAGGCA NC_027974_Sanionia
CCACAAGG--TACGAGCCAGAACCTTATATTATAGGTCGGGTAGGACGGCACTACTGGCAAATACTATCTAGCAAAGGCA NC_024515_Climacium
CCACAAGG--TACGAGCCAGAACCTTATA-----GGTCGGGTAGGACGGCACTACTGGCAAATACTATCTAGCAAAGGCA NC_031212_Brachythecium
CTGCAAGG--GACAAACCAAAACCTCA-------GGTCGGGTAGGATGGCACTACTGGCAAATACTATCTAGCGAAGACA NC_024520_Atrichum
CCGTAAAG--GACAAGCCAAAATATCA-------GGTCGGGTAGGATGGCACTACTGGCAAATACTATCTAGCGAAGACA NC_024521_Sphagnum
CCACAAGG--TACGAGCCAGAACCTTATA-----GGTCGGGTAGGACGGCACTACTGGCAAATACTATCTAGCAAAGGCA NC_024516_Hypnum
CCGCGAGGGCGCAGCACCAAAACCTCA-------GGTCGGGTAGGATGGCACTACTGACAAATACTATCTAGCGAAGACA KC 784953 Tetraphis
TT-TAAA------ACCATACATTTTTCTA-----AATAGAGATGCGTGGGACAGAGGGCTCGTAGTACCTGCCT-AGCCT MF 417767 Mielichhoferia
-------------------------------------------------------------------------------- NC 024518_Buxbaumia

        730       740       750       760       770       780       790       800
....|....|....|....|....|....|....|....|....|....|....|....|....|....|....|....|
CGAGTCGTGAAGGATAAAGCTTGCGTCAAAAGCATACGGAAAGATTCTAGCAACGAGGAAACCGGGGGAGGAA-CCGGTA NC_027974_Sanionia
CGAGTCGTGAAGGATAAAGCTTGCGTCAAAAGCATACGGAAAGATTCTAGCAACGAGGAAACCGGGGGAGGAA-CCGGTA NC_024515_Climacium
CGAGTCGTGAAGGATAAAGCTTGCGTCAAAAGCATACGGAAAGATTCTAGCAACGAGGAAACCGGGGGAGGAG-CCGGTA NC_031212_Brachythecium
CGAGCCTTAGGGGATAAGGGCTGCGTCGAAAGCATACGGGAAAATTCTAGCAACAAGGAAACCGGGGA-GGAAGCCGGTA NC_024520_Atrichum
CAGGCCGGAG----TAAGGCTTGCGTCGAAAGCATACAGGAAAATTCCAGCAACAAGGAAACCGAGGG--GAA-CCGGTA NC_024521_Sphagnum
CGAGTCGTGAAGGATAAAGCTTGCGTCAAAAGCATACGGAAAGATTCTAGCAACGGGGAAACCGGGGG-AGGAACCGGTA NC_024516_Hypnum
CAAGGGCGCA-----GCGACTTGCGTCGAAAGCATACGGGAAAATTCTAGCAACGAGGAAACCGGGGGGGGGAACCGGTA KC 784953 Tetraphis
TTGCTTCGAG-----AACCATGTAAACGAAGAAA---GGAAG--TGCT-GCT----GGAAAGCGAAAGGAAAGAGGCCTA MF 417767 Mielichhoferia
-------------------------------------------------------------------------------- NC 024518_Buxbaumia

        810       820       830       840       850       860       870       880
....|....|....|....|....|....|....|....|....|....|....|....|....|....|....|....|
GAGCTGCAAGAGCATAACCGGAA----GGTCAAGCCAGGGAGGCCGGGTCATTTAACGGAAGTGGAAAGGTTCGAATCAA NC_027974_Sanionia
GAGCTGCAAGAGCATAACCGGAA----GGTCAAGCCAGGGAGGCCGGGTCATTTAACGGAAGTGGAAAGGTTCGAATCAG NC_024515_Climacium
GAGCTGCAAGAGCATAACCGGAA----GGTCAAGCCAGGGAGGCCGGGTCATTTAACGGAAGTGGAAAGGTTCGAATCAA NC_031212_Brachythecium
GAGCTACAAGAACATAACCGGAA----AGTCAAGCCAGGGAGGCCGGGTCATTTAACGGAAATGGAAAGCTTCGAATAAG NC_024520_Atrichum
GAGCTGCAGGAGCATAACTGGAA----GGTCAAGTCAGGGAGGCCAGGTCATTTAACGGAAATGGAAAGGTTCGAATAAG NC_024521_Sphagnum
GAGCTGCAAGAGCATAACCGGAA----GGTCAAGCCAGGGAGGCCGGGTCATTTAACGGAAGTGGAAAGGTTCGAATCGA NC_024516_Hypnum
GAGCTGCAAGAGCATGACCGGAA----GGTCAAGCCAGGGAGGCGGGGTCATTTAACGGAAATGAAAAGGTTTGAATAGA KC 784953 Tetraphis
AATCAGCAGACTGACGCCGCGCACTTGAGCAGTTCGGAGAAGACCGGCCT---------AATCCAGACTCTTT-AGAAAT MF 417767 Mielichhoferia
-------------------------------------------------------------------------------- NC 024518_Buxbaumia

        890       900       910       920       930       940       950       960
....|....|....|....|....|....|....|....|....|....|....|....|....|....|....|....|
GGCTGAAAGAAAAGGGAGAAATAGATAGCTCGTAGGACCCCACTGTAGTTGAAACGC--------GGGGC--AAGACTGC NC_027974_Sanionia
GGCTGGAAGAAAAGGGAAAAATAGGTAGCTCGTAGGACCCCACTGTAGTTGAAACGC--------GGGGC--AAGACTGC NC_024515_Climacium
GGCTGAAAGAAAAGGGAAAAATAGGTAGCTCGTAGGACCCCACTGTAGTTGAAATGC--------GGGG---CAAGACTG NC_031212_Brachythecium
GGCTGGAAGAGAAAGTGAAAATGAGTAGCTTATAGGACCCCACTGTAGTTGGGGCGTAGGACCATAGGGCC-AAGGCCGC NC_024520_Atrichum
AGCTAGAAGAGGAAATGATAATAGGTAGCCT----GACCCCACTGTGGTTGGACTGTAGGGCCATAGGGCC-AAGGCCGC NC_024521_Sphagnum
GGCTGAAAGAAAAGGGAAAAATAGGTAGCTCGTAGGACCCCACTGTAGTTGAAACGC--------GGGGC--AAGACTGC NC_024516_Hypnum
AGCTGGGGGAGGCAGTAAAAATGGGTAGCTCGTAGGACC------TGGTTGGAGCGTAGGGCC-ATGGGCCGAAGGCCGC KC 784953 Tetraphis
TAAGCCAGAATGCGCAACTTTCAAGAAACGAAGGAAGTCCGTTAATATTTGGTTCGTTCG--C-TAGCGCA-TAAACCA- MF 417767 Mielichhoferia
-------------------------------------CCCCACTATGATCGGAGCAC--------AGAGC--CAAGGCCA NC 024518_Buxbaumia
```

```
         970       980       990      1000      1010      1020      1030      1040
....|....|....|....|....|....|....|....|....|....|....|....|....|....|....|....|
-GGGTGTTGGAT-ACGACAGATGGCGCTGCCTAAACTTCATGGAATTCCATGAACT---AGGGAAACAAGCAGTCTCAAA NC_027974_Sanionia
-GGGTGTTGGAT-ACGACAGATGGCGCTGCCTAAACTTCATGGAATTCCATGAACT---AGGGAAACAAGCAGTCTCAAA NC_024515_Climacium
CGGGTGTTGGAT-ACGACAGATGGCGCTGCCTAAACTTCATGGAATTCCATGAACT---AGGGAAACAAGCAGTCTCAAA NC_031212_Brachythecium
AGGTTA-AGGGT-ACGACAGACAGCGCTGCCCACACTTCATGGAATCACATGAACT----GGAAGAAAAGCAGTCTCAAA NC_024520_Atrichum
TGGTTG-ACAGT-ATGATAGACAGTGCTGTCTGCACTTCATAGAATTGCATAAACTA--AAAGAAACAAGCAGTCTCAAA NC_024521_Sphagnum
-GGGTGTTGGAT-ACGACAGATGGCGCTGCCTAAACTTCATGGAATTCCATGAACT---AGGGAAACAAGCAGTCTCAAA NC_024516_Hypnum
AGGGCGCAGCAT-ACGACAGACAGCGCTGTCTACACTTCATGAA----ATTCCATG-------AAAAAAGCAGTCTCGAA KC 784953 Tetraphis
-GGCAGACGCTTTATTCGAACCAGAGCT-TCATCAC-CCAAAAG--CGAAAATACTTCTGAAGTCAAAACTCAAC-CATT MF 417767 Mielichhoferia
CAAGTTAGGGATATGACAGACAACGCTGCCTACACTTTTCCCTTTATGTAATTCCACAAGT--AGAAAAGCAGTCTCAAA NC 024518_Buxbaumia

        1050      1060      1070      1080      1090      1100      1110      1120
....|....|....|....|....|....|....|....|....|....|....|....|....|....|....|....|
TTTGCGCATGCACATTTCCAAGCTACCAAAACGGCTGCAGAGCAT---AGCATATATATCTAT----TTTTTTTT-GCAG NC_027974_Sanionia
TTTGCGCATGCACATTTCCAAGCTACCAAAACGGCTGCAGAGCAT---AGCATATATATCTAT----TTTTTTTT-GCAG NC_024515_Climacium
TTTGCGCATGCACATTTCCAAGCTACCAAAACGGCTGCAGAGCAT---AGCATATATATCTAT----TTTTTTTT-GCAG NC_031212_Brachythecium
TTTACGCATGCGCATTTCCAAGCTACTAAAAAGGCTGCAGGGCGC---AGCGTATATATATA-----TTCTTTTT-GCAG NC_024520_Atrichum
ATGATGCATGCGCATTTCTGAGCTACCAAAGGGGCCGCAGGGCGC---AGCATATATATATA-----TTTTTTTT-GCAG NC_024521_Sphagnum
TTTGCGCATGCACATTTCCAAGCTACCAAAACGGCTGCAGAGCAT---AGCATATATATCTAT-----TTTTTTTTGCAG NC_024516_Hypnum
TTTACGCATGCACATTTCCAAGCTACCGAAAAGGATGCAGGGCGC---AGCATATATATATATATATATTTTCTTCGCAG KC 784953 Tetraphis
ATGACGC-TGCGCTCCTGGTTTGCTTATTTAAAATTTAAGGGTAACTCAAGTTAGAGAGCCGTGTG-ATGGGT---GACC MF 417767 Mielichhoferia
AATGCG-ATGCGCATTTCCAAGCTACCAAAACGGCTGCAGGGTACA--AGCATATATATATATACATGTTTTTT---CAG NC 024518_Buxbaumia

        1130      1140      1150      1160      1170      1180      1190      1200
....|....|....|....|....|....|....|....|....|....|....|....|....|....|....|....|
GCTTCAGACCTTCTTT-ATTGAATCTCGGGCCACCTGTAATAAATGG-CGCGAGCCGTATGCGAGGAGACTTGCACGTAC NC_027974_Sanionia
GCTTCAGACCTTCTTT-ATTGAATCTCGGGCCACCTGTAATAAATGG-CGCGAGCCGTATGCGAGGAGACTTGCACGTAC NC_024515_Climacium
GCTTCAGACCTTCTTT-ATTCAATCTCGGGCCACCTGTAATAAATGG-CGCGAGCCGTATGCGAGGAGACTTGCACGTAC NC_031212_Brachythecium
GCCTTAGGCTTTTTTTTATCGAATGTTAGGCCACCTGTAATAAATGG-CGCGAGCCGTATGCGGGGAGACTTGCACGTAC NC_024520_Atrichum
GCCTCAGTCCTCTTTC---CGAATGTTGGGCCACCTGTAACAAATGG-CGTGAGCCGTATGCGGGGAGACTTGCACGTAT NC_024521_Sphagnum
GCTTCAGACCTTCTTT-ATTGAATCTCGGGCCACCTGTAATAAATGG-CGCGAGCCGTATGCGAGGAGACTTGCACGTAC NC_024516_Hypnum
GCCTCAGGCTTTTTTT-ATCGCATGTTGAGCCACCTGTAATAAGTGG-CGTGAGCCGTATGCGGGGAGACTTGCACGTAC KC 784953 Tetraphis
ATCTCA--CACGGTTC-AGGGAGCATTTTATTATGTGATGCGAGTGA-AT------GTGTACAGCATAGCTGGCATCAAT MF 417767 Mielichhoferia
GCTTTTTTTACCTGAAAATTTTTT---GGATC----GTAATAAGTGATGTGAACTATCT-GCAA-------TCTATTCGC NC 024518_Buxbaumia

                                         Exon 2
        1210      1220      1230      1240      1250      1260      1270      1280
....|....|....|....|....|....|....|....|....|....|....|....|....|....|....|....|
GGTTCTTAGGGGGG-TTCCGGCCGAAAGATCGGCGCCTACCCGAC--TAGAGGCACAGAAAAATTAATCGAGTATAAAAC NC_027974_Sanionia
GGTTCTTAGGGGGG-TTCCGGCCGAAAGATCGGCGCCTACCCGAC--TAGAGGCACAGAAAAATTAATCGAGTATAAAAC NC_024515_Climacium
GGTTCTTGGGGGGG-TTCCGGCCGAAAGATCGGCGCCTACCCGAC--TAGAGGCACAGAAAAATTAATCGAGTATAAAAC NC_031212_Brachythecium
GGTTCTTAGGGGGG-TTCCGGCCGAAAGACCGGCGCCTACCCAAC--TAGAGGCACAGAAAAATTAATCGAGTATAAAAC NC_024520_Atrichum
GGTTCTTAGGGGGGTTTTTGGCCAAAAGACCGACGCCTACCCGAC--TAGAGGCACAGAAAAATTAATTGAGTATAAAAC NC_024521_Sphagnum
GGTTCTTAGGGAGG-TTCCGGCCGAAAGATCGGCGCCTACCCGAC--TAGAGGCACAGAAAAATTAATCGAGTATAAAAC NC_024516_Hypnum
GGTTCTTGGGGGG--TTCCGGCCGAAAGACCGGCGCCTACCCGAC--TAGAGGCACAGAAAAATTAATCGAGTATAAAAC KC 784953 Tetraphis
TAAATTTTGAC----TTTAT------------------------------------------------------------ MF 417767 Mielichhoferia
GGTTC------------------------------------------------AATCTAAAAATACATTTATTATAAATT NC 024518_Buxbaumia

                            Intron 2
        1290      1300      1310      1320      1330      1340      1350      1360
....|....|....|....|....|....|....|....|....|....|....|....|....|....|....|....|
TTATCTTCAAGCTTTACCTTATTTTGATCG--TTTAGAGGGCGACCGCGAAGTCATCGAATGAACTCCTCCAGAATGGAG NC_027974_Sanionia
TTATCTTCAAGCTTTACCTTATTTTGATCG--TTTAGAGGGCGACCGCGAAGTCATCGAATGAACTCCTCCAGAATGGAG NC_024515_Climacium
TTATCTTCAAGCTTTACCTTATTTTGATCG--TTTAGAGGGCGACCGCGAAGTCATCGAATGAACTCCTCCAGAATGGAG NC_031212_Brachythecium
GTATCTTCAAGCTTTACCTTATTTTGATCG--TTTAGAGGGCGACCGCGAAGTCACCGAATGAACTCCTCTAAAATGGAG NC_024520_Atrichum
GTACCTTCAAGCTTTACCTTATTTTGATCG--TTTAGAGGGCGACTGTGAAGTCACCAAATGAACTCCTCCGAAAAGGAG NC_024521_Sphagnum
TTATCTTCAAGCTTTACCTTATTTTGATCG--TTTAGA------------------------------------------ NC_024516_Hypnum
GTATCTTCAAGCTTTACCTTATTTTGATCG--TTTAGAGGGCGACCGCGAAGTTACCGAATGAATTCCTCCAAAATGGAG KC 784953 Tetraphis
-------------------------------------------------------------------------------- MF 417767 Mielichhoferia
ATA----------------------------------------------------------------------------- NC 024518_Buxbaumia

        1370      1380      1390      1400      1410      1420      1430      1440
....|....|....|....|....|....|....|....|....|....|....|....|....|....|....|....|
GTGCTGCAGAAACCCGCAGCAAAACAGATACGACTAATCGACATATAAAATATGGCGACGACGACAGCATGTCGTAAAAG NC_027974_Sanionia
GTGCTGCAGAAACCCGCGGCAAAACAGATACGACTAATCGACATATAGAATATGGCGACGACGACAGCATGTCGTAAAAG NC_024515_Climacium
GTGCTGCAGAAACCCGCAGCAAAACAGATACGACTAATCGACATATAGAATATGGCGACGACGACAGCATGTCGTAGAAG NC_031212_Brachythecium
ATGCTGCAGAAACCCGCGGCAAAGCAGATACGACTGATCGACATATAGAATATGGCGACGACGACAGCATGTCGTAAAAG NC_024520_Atrichum
GTGCTGCAGATACCCGCGGCAAAGCAGATATGACTAATCGACATATAGAATATGACGACAACAACAGCATGTCGTAAAAG NC_024521_Sphagnum
-------------------------------------------------------------------------------- NC_024516_Hypnum
GTGCTGCGGAAACCCGCAGCAAAACAGATACGACTAATCGACATATAGAATATGGCGACGACGACAGCATGTCGTAAGAG KC 784953 Tetraphis
-------------------------------------------------------------------------------- MF 417767 Mielichhoferia
-------------------------AGATGCGTTTAG------------------------------------------- NC 024518_Buxbaumia
```

```
      1450      1460      1470      1480      1490      1500      1510      1520
....|....|....|....|....|....|....|....|....|....|....|....|....|....|....|....|
GAGATAACTGGGAATAGCCAACCCTTGGCCGTATCTTCAACTGCATCCTTGAGTGTCAGTCAAATGGAAAGTATCATGAA NC_027974_Sanionia
GAGATAACTGGGAATAGCCAACCCTTGGCCGTATCTTCAACTGCATCCTTGAGTGTCAGTCAAATGGAAAGTATCATGAA NC_024515_Climacium
GAGATAACTGGGAATAGCCAACCCTTGGCCGTATCTTCAACTGCATCCTTGAGTGTCAGTCAAATGGAAAGTATCATGAA NC_031212_Brachythecium
GAGATAACTGGGAATAGCCAACCCTTAGCCTTATTTCTAACTGCATCCTTGAGTGTCAGTTAAACAGAAAGTATCGTGAA NC_024520_Atrichum
AAGATAACTAG----AGCCAACCCTTGGTG-TATTTCCAACTACATTCCTGAGTGTCAGTTAAACAAAGAATATCGTAAA NC_024521_Sphagnum
-------------------------------------------------------------------------------- NC_024516_Hypnum
AAGATAACTGGGAATAGCCAACCC-------------------------------------------------------- KC 784953 Tetraphis
-------------------------------------------------------------------------------- MF 417767 Mielichhoferia
------------------------------------------------------------------------ATAATGAA NC 024518_Buxbaumia

      1530      1540      1550      1560      1570      1580      1590      1600
....|....|....|....|....|....|....|....|....|....|....|....|....|....|....|....|
TGAGAGATGCCAGCGGAATGATCACCTGGGCGCGCTAGGCTAGAAGGAAAATAAGCTTCTCTT----------------- NC_027974_Sanionia
TGAGAGATGCCAGCGGAATGATCACCTGGGCGCGCTAGGCTAGAAGGAAAATAAGCTTCCCTT----------------- NC_024515_Climacium
TGAGAGATGCCAGCGGAATGATCACCTGGGCGCGCTAGGCTAGAAGGAAAATAAGCTTCCCTT----------------- NC_031212_Brachythecium
TGAGAGATGCCGGCGGAATGATCACCCGGACGTGCTGGGCTAGGAGGAGAATAAGCTTACTCTTTATTCTTTTGGCGTTC NC_024520_Atrichum
TGAAAGATGCCAGCGGAATGATCAGCCGGACGTGCTAGGCTAGGAAGAAAAAAAGCTTGCTTCTTATTTCTTTGGCCTTT NC_024521_Sphagnum
-------------------------------------------------------------------------------- NC_024516_Hypnum
-------------------------------------------------------------------------------- KC 784953 Tetraphis
-------------------------------------------------------------------------------- MF 417767 Mielichhoferia
TGGATAATATTAGCAGA------------------------------------------CTTT----------------- NC 024518_Buxbaumia

      1610      1620      1630      1640      1650      1660      1670      1680
....|....|....|....|....|....|....|....|....|....|....|....|....|....|....|....|
---------------GTAACAAAGTA---------------------------------------------AGGCAAAAT NC_027974_Sanionia
---------GGCAAGATAACAAAGTA---------------------------------------------AGGCAAAAT NC_024515_Climacium
---------GGCAAGATAACAAAGTA---------------------------------------------AGGCAAAAT NC_031212_Brachythecium
GTTCGTAAAGACAACAAGGTAGAGCAGAAAATAAAAAA-GGTGCAAAGCGAAAAAAAAATATCTAATCCCCAAACAAAAT NC_024520_Atrichum
GTTCATAGGGAGGAAAAAATAGAGCAAGAAAAAGGAAAAGGCGCAAAGCGAAAAAAGAATATCCC---------CAAAAT NC_024521_Sphagnum
-------------------------------------------------------------------------------- NC_024516_Hypnum
-------------------------------------------------------------------------------- KC 784953 Tetraphis
-------------------------------------------------------------------------------- MF 417767 Mielichhoferia
---------GATCAAA---------------------------------------------------------------- NC 024518_Buxbaumia

      1690      1700      1710      1720      1730      1740      1750      1760
....|....|....|....|....|....|....|....|....|....|....|....|....|....|....|....|
ATTTTTTTTTGCTGGCTTTCAGTTTTCTGAGTGCA---GCCTCCTCCTACAACGTCTTTCTTCGTGTGTCGAAGATCTGA NC_027974_Sanionia
ATTTTTTTTTGCTGGCTTTCAGTTTTCTGAGTGCA---GCCTCCTCCTACAACGTCTTTCTTCGTGTGTCGAAGATCTGA NC_024515_Climacium
ATTTTTTTTTGCTGGCTTTCAGTTTTCTGAGTGCA---GCCTCCTCCTACAACGTCTTTCTTCGTGTGTCGAAGATCTAA NC_031212_Brachythecium
ATTTTTTTTTGCTGGCTTGTAGTTTTATGAATGCATTCTCCCCCCCCCACAACGTCTTTCCTTGTGTGTCGAAGATCTAA NC_024520_Atrichum
ATTTTTTTTT-CTGGTTTGTCATTTTCTGGGTTTA---GCCTCCCCCCACAACGTCTTTCCTTGTGTGTTGAAGATTTAA NC_024521_Sphagnum
-------------------------------------------------------------------------------- NC_024516_Hypnum
-------------------------------------------------------------------------------- KC 784953 Tetraphis
-------------------------------------------------------------------------------- MF 417767 Mielichhoferia
-------------------------------------------------------------------------------- NC 024518_Buxbaumia

      1770      1780      1790      1800      1810      1820      1830      1840
....|....|....|....|....|....|....|....|....|....|....|....|....|....|....|....|
GGCGAGTGCACCAGAGATAGAAACAGAAACTACGATTCAATATGAATATAGCAAAGCATCTGAAGCATAACTACACACTC NC_027974_Sanionia
GGCGAGTACACCAGAGATAGAAACAGAAACTACGATTCAATATGAATATAGCAAAGCATCTGAAGCATAACTACACACTC NC_024515_Climacium
GGCGAGTACACCAGAGATAGAAACAGAAACTACGATTCAATATGAATATAGCAAAGCATCTGAAGCATAACTACACACTC NC_031212_Brachythecium
AGCGAGTACACCGGAGATAGAAAGGGAAACTAGGATTCAATATGAATATAGCAGAGCATCTAAAACATAACTACACACTC NC_024520_Atrichum
AGCAAGTGCACCAAGGTTAAAAACGGAAACTAGGATTCAATATGAATATAGCAAAACATCTAGAGCATAACTACACACTC NC_024521_Sphagnum
-------------------------------------------------------------------------------- NC_024516_Hypnum
-------------------------------------------------------------------------------- KC 784953 Tetraphis
-------------------------------------------------------------------------------- MF 417767 Mielichhoferia
-------------------------------------------------------------------------------- NC 024518_Buxbaumia

      1850      1860      1870      1880      1890      1900      1910      1920
....|....|....|....|....|....|....|....|....|....|....|....|....|....|....|....|
ATACGATCTTGTGAAAAGATAGGAGCATTCGGTGGAACCGGTGAACCATACATTTTTCTAGATAGAGATGCGTGGGACAG NC_027974_Sanionia
ATACGATCTTGTGAAAAGATAGGAGCATTCGGTGGAACCGGTGAACCATACATTTTTCTAGATAGAGATGCGTGGGACAG NC_024515_Climacium
ATACGATCTTGTGAAAAGATAGGAGCATTCGGTGGAACCGGTGAACCATACATTTTTCTAGATAGAGATGCGTGGGACAG NC_031212_Brachythecium
ACACAATCTTGTAAAGAGATAGGAGCATTCGGTGAAACCGGTGAACCACACATTTTTATAGATAGAGATGTGTGGGACAG NC_024520_Atrichum
ACACAATCTTGTAGAGAGATAGGAGCATTTAGTGAAACCAGTGAACCACACATCCTTCTAGATAGA--TGCGTAGGACAG NC_024521_Sphagnum
-------------------------------------------------------------------------------- NC_024516_Hypnum
-------------------------------------------------------------------------------- KC 784953 Tetraphis
-------------------------------------------------------------------------------- MF 417767 Mielichhoferia
-------------------------------------------------------------------------------- NC 024518_Buxbaumia
```

```
         1930      1940      1950      1960      1970      1980      1990      2000
....|....|....|....|....|....|....|....|....|....|....|....|....|....|....|....|
AGGGCTCGTAGTACCTGCCCCAGCCTTTGCTTCGAGAACCATGTAAACGAAGAAAGGAAGTGCTGCTGGAAAGCGGAAAG NC_027974_Sanionia
AGGGCTCGTAGTACCTGCCCCAGCCTTTGCTTTGAGAACCATGTAAACGAAGAAAGGAAGTGCTGCTGGAAAGCGGAAGG NC_024515_Climacium
AGGGCTCGTAGTACCTGCCCTAGCCTTTGTTTCGAGAACCCTGTGAACGAAGAAAAGAAGTGCTGCTGGAAAGCGGAAGG NC_031212_Brachythecium
AGGGCTCGTAGTACCTGCCT-GCCCTTCGCTCCGAGGACCATGTGAACGAAGGAAGGGAGTGCTGCTGTGAAGCAAAAGG NC_024520_Atrichum
AGGGCTCGTAGTACCTGCCT-ACCCTTTGCTTTGAGAACCATGTAAACAGAGAGAGAGAGTGCTGCTTTACAGCAGAAGG NC_024521_Sphagnum
-------------------------------------------------------------------------------- NC_024516_Hypnum
-------------------------------------------------------------------------------- KC 784953 Tetraphis
-------------------------------------------------------------------------------- MF 417767 Mielichhoferia
--------------------------TTGCTTCGA----------AGTGCGTAAAAGAAGTACGAATTAA---------- NC 024518_Buxbaumia

         2010      2020      2030      2040      2050      2060      2070      2080
....|....|....|....|....|....|....|....|....|....|....|....|....|....|....|....|
AAAGGAGCCTAAGTCGGCAGACTGACGCCGCGCACTTGAGCAGTTCGGAGAAG--------------------------- NC_027974_Sanionia
AAAGGAGCCTAAGTCGGCAGACTGACGCCGCGCACTTGAGCAGTTCGGAGAAG--------------------------- NC_024515_Climacium
AAAGGAGCCTAAGTCGGCAGACTGACGCCGCGCACTTGAGCAGTTCGGAGAAG--------------------------- NC_031212_Brachythecium
GAAGAAGCCTAAATCGGCGGACTAATGTCGCGCACTTGAGCAGTGCGGAGAAGGCCGGTCAAAACCCGGAGGGGGAAAAA NC_024520_Atrichum
GAAGGGGCCTAAATCGGCGGACTGACGTCGCACACTTGAGCAGTGCGGAGAAGGCTGGTCAAAAAAGGGAGGGGAAAAAA NC_024521_Sphagnum
-------------------------------------------------------------------------------- NC_024516_Hypnum
-------------------------------------------------------------------------------- KC 784953 Tetraphis
-------------------------------------------------------------------------------- MF 417767 Mielichhoferia
---------TAAAATGGTCGGTTAGCACTGCATA-----------TAATCGAA--------------------------- NC 024518_Buxbaumia

         2090      2100      2110      2120      2130      2140      2150      2160
....|....|....|....|....|....|....|....|....|....|....|....|....|....|....|....|
----------------ACCAGCCTACTCCATACTCTTTAGAAATTAAGCCAGAATGCGCAACTTTTAAGAAACGAAGGAA NC_027974_Sanionia
----------------ACCAGCCTACTCCATACTCTTTAGAAATTAAGCCAGAATGCGCAACTTTTAAGAAACGAAGGAA NC_024515_Climacium
----------------ACCAGCCTACTCCATACTCTTTAAAAATTAAGCCAGAATGCGCAACTTTTAAGAAACGAAGGAA NC_031212_Brachythecium
ATATATTTTTTTCGAG-CCAGTCTACTCCATACTCTCTGAAAATTAAGCCAGAATGCACGACTTCCAGGAAACGAAAAAA NC_024520_Atrichum
ATTGATTTTTTTT----------TACTCTATACCCTTCGAAGATGAAGCCAGAATGCACGATTTCCAGGAAAGGAAGGAA NC_024521_Sphagnum
-------------------------------------------------------------------------------- NC_024516_Hypnum
-------------------------------------------------------------------------------- KC 784953 Tetraphis
-------------------------------------------------------------------------------- MF 417767 Mielichhoferia
----------------TCCAGGCGGCTATCAAATCATCTACGACGATGTTGTACCGCGTAACATGTGCAGATCGAAAAAA NC 024518_Buxbaumia

         2170      2180      2190      2200      2210      2220      2230      2240
....|....|....|....|....|....|....|....|....|....|....|....|....|....|....|....|
GTCCGTTAATATTTGGTTC-----------GTTCGCTAGCGCATAAACCAGGCAGACGCTTTATTCGAACCAGAGC-TT- NC_027974_Sanionia
GTCCGTTAATATTTGGTTC-----------GTTCGCTAGCGCATAAACCAGGCAGACGCTTTATTCGAACCAGAGC-TT- NC_024515_Climacium
GTCCGTTAATATTTGGTTC-----------GTTCGCTAGCGCATAAACCAGGCAGACGCTTTATTCGAACCAGAGC-TT- NC_031212_Brachythecium
GTCACTGAATATTTGATTCGTTGCTTATTTGTTTGTTGGCGCATAAAGCAGTCAGACGCTTTGTTCAAACCAAAGCCTTG NC_024520_Atrichum
GTCATTGAATATTGGGTTTGTCGCCTATTTGTTTGCTGGCGCATAAACTAGGCAGGCACTTTCTTCAAACCAAACCTTTG NC_024521_Sphagnum
-------------------------------------------------------------------------------- NC_024516_Hypnum
-------------------------------------------------------------------------------- KC 784953 Tetraphis
-------------------------------------------------------------------------------- MF 417767 Mielichhoferia
-------AATATTTA---------------------TAGGACATGAACAAA----------------------------- NC 024518_Buxbaumia

         2250      2260      2270      2280      2290      2300      2310      2320
....|....|....|....|....|....|....|....|....|....|....|....|....|....|....|....|
CATCACCCAGAAGCGAAAATACTTCTGAAGT-----------C-GAAACTCAACCATTATGACGCTGCGCTCCTGGTTTG NC_027974_Sanionia
CATCACCCAGAGGCGAAAATACTTCTGAAGT-----------C-GAAACTCAACCATTATGACGCTGCGCTCCTGGTTTG NC_024515_Climacium
CATCACCCAGAAGCGAAAATACTTCTGAAGT-----------C-GAAACTCAACCATTATGACGCTGCGCTCCTGGTTTG NC_031212_Brachythecium
TTATACCCAAAAGCAGAAATACTTTTGAAGT-----------CAAAAACTCAACCATTATGACGCTGCGCCCTTGGCCTA NC_024520_Atrichum
T--CATCTAGAAGC---TCTTATATGGAAGTACTTCTAAGGGCAAAAACTCAACCATTATGACGCTGCGCTCTTGGCCTG NC_024521_Sphagnum
-------------------------------------------------------------------------------- NC_024516_Hypnum
-------------------------------------------------------------------------------- KC 784953 Tetraphis
-------------------------------------------------------------------------------- MF 417767 Mielichhoferia
------------------------------------------------------------AATGATGCACACTTAGTGTG NC 024518_Buxbaumia

         2330      2340      2350      2360      2370      2380      2390      2400
....|....|....|....|....|....|....|....|....|....|....|....|....|....|....|....|
CTTATCTAAAATCTAAGGGTAACTCAAGTTAGAGAGCCGTGTGATGGGTGACCATCTCACACGGTTCGGGGAGCACTTTA NC_027974_Sanionia
CTTATCTAAAATCTAAGGGTAACTCAAGTTAGAGAGCCGTGTGATGGGTGACCATCTCACACGGTTCGGGGAGCACTTTA NC_024515_Climacium
CTTATCTAGAATCTAAGGGTAACTCAAGTTAGAGAGCCGTGTGATGGGTGACCATCTCACACGGTTCGGGGAGCACTTTA NC_031212_Brachythecium
GTTATTCAAAACCTAAGGGTAACTTAAGTTGGAGAGCCGTGTGATGGGTGACCATCTCACACGGTTCGAAGAGCACTTTA NC_024520_Atrichum
GGGATTGAAAACCTGAGG-TAACTCAAGTTGGAGAGCCGTGTGATGGGTGACCATCTCGCACGGTTCGGAGAGCACTTTT NC_024521_Sphagnum
-------------------------------------------------------------------------------- NC_024516_Hypnum
-------------------------------------------------------------------------------- KC 784953 Tetraphis
-------------------------------------------------------------------------------- MF 417767 Mielichhoferia
CTTA----------------------------------------------------------------------GCTTTC NC 024518_Buxbaumia
```

```
                                                                          Exon 3
        2410      2420      2430      2440      2450      2460      2470      2480
....|....|....|....|....|....|....|....|....|....|....|....|....|....|....|....|
TTATGTGATGCGAGTGAATGTGTGCGGCATAGCTGGCATCAATTAAATTCTGACTCTAT--TTATGTTTCTATGATGGCC NC_027974_Sanionia
TTATGTGATGCGAGTGAATGTGTGCGGCATAGCTGGCATCAATTAAATTCTGACTCTAT--TTATGTTTCTATGATGGCC NC_024515_Climacium
TTATGTGATGCGAGTGAATGTGTGCGGCATAGCTGGCATCAATTAAATTCTGACTCTAT--TTATGTTTCTATGATGGCC NC_031212_Brachythecium
TTATGTGATGCGAGTGAATGTGTACAGCATAGCTGGTATCAATTAAATTTTGACTCTAT--TTATGTTTCTATGATGGCC NC_024520_Atrichum
GTATGTAATGTAAGTAAATGTGTACAGCATAGCTGGCATTAATGAATTTTTGACTCTAT--TTATGTTTCTATGATGGCC NC_024521_Sphagnum
-------------------------------------------------------------TTACGTTTCTATGATGGCC NC_024516_Hypnum
-------------------------------------------------------------------------------- KC 784953 Tetraphis
-------------------------------------------------------------TTATGTTTATATGATGGTC MF 417767 Mielichhoferia
TTATAAGA------------------------------------------------------------------------ NC 024518_Buxbaumia

        2490      2500      2510      2520      2530      2540      2550      2560
....|....|....|....|....|....|....|....|....|....|....|....|....|....|....|....|
CAAGAACATGCTTATTCTTTAGCTGTAGAGAAACTTTGTAATTGTGAGGTACCATTACGAGCTCAGTATATACGAGTGTT NC_027974_Sanionia
CAAGAACATGCTTATTCTTTAGCTGTAGAGAAACTTTGTAATTGTGAGGTACCATTACGAGCTCAGTATATACGAGTGTT NC_024515_Climacium
CAAGAACATGCTTATTCTTTAGCTGTAGAGAAACTTTGTAATTGTGAGGTACCATTACGAGCTCAGTATATACGAGTGTT NC_031212_Brachythecium
CAAGAACATGCTTATTCCTTGGCCGTAGAGAGACTTTGTAATTGTGAGGTACCATTACGAGCTCAGTATATACGAGTGTT NC_024520_Atrichum
CAAGAACATGCTTATTCTTTAGCTGTAGAGAGACTTTGTAATTGTGAAGTACCACTACGAGCTCAGTATATACGAGTGTT NC_024521_Sphagnum
CAAGAACATGCTTATTCTTTAGCTGTAGAGAAACTTTGTAATTGTGAGGTACCATTACGAGCTCAGTATATACGAGTGTT NC_024516_Hypnum
-------------------------------------------------------------------------------- KC 784953 Tetraphis
CAAGAACATGCTTATTCTTTAGCCGTAGAAAAACTTTATAATTGTAAGGTACCATTACGAGCTCAGTATATACGAGTGTT MF 417767 Mielichhoferia
CAAGATCATAGCGCTTATGTAG---------------------------------------------------------- NC 024518_Buxbaumia

        2570      2580      2590      2600      2610      2620      2630      2640
....|....|....|....|....|....|....|....|....|....|....|....|....|....|....|....|
ATTTCGTGAAATAACTCGAATTTTAAATCATTTACTTGCTTTAACTACTCATGCTATGGATGTGGGGGCATTAACTCCGT NC_027974_Sanionia
ATTTCGCGAAATAACTCGAATTTTAAATCATTTACTTGCTTTAACTACTCATGCTATGGATGTGGGGGCATTAACTCCGT NC_024515_Climacium
ATTTCGCGAAATAACTCGAATTTTAAATCATTTACTTGCTTTAACTACTCATGCTATGGATGTGGGGGCATTAACTCCGT NC_031212_Brachythecium
ATTTCGTGAAATAACTCGAATTTTAAATCATTTACTTGCTTTAACTACTCATGCTATGGATGTGGGAGCATTGACTCCGT NC_024520_Atrichum
ATTCTGTGAAATAACTCGAATTTTAAATCATTTACTTGCTTTAACTACTCATGCTATGGATGTAGGAGCATTAACTCCAT NC_024521_Sphagnum
ATTTCGTGAAATAACTCGAATTTTAAATCATTTACTTGCTTTAACTACTCATGCTATGGATGTGGGGGCATTAACTCCGT NC_024516_Hypnum
-------------------------------------------------------------------------------- KC 784953 Tetraphis
ATTTCGTGAAATAACTCGAATTTTAAATCATTTACTTGCTTTAACTACTCATGCTATGGATGTGGGGGCATTAACTCCGT MF 417767 Mielichhoferia
-------------------------------------------------------------------------------- NC 024518_Buxbaumia

        2650      2660      2670      2680      2690      2700      2710      2720
....|....|....|....|....|....|....|....|....|....|....|....|....|....|....|....|
TCCTGTGGGCCTTT--GAAGAGCGAGAAAAACTTTTAGAATTCTATGAAAGAGTTTCAGGAGCCAGGATGCATGCCAGTT NC_027974_Sanionia
TCCTGTGGGCCTTT--GAAGAGCGAGAAAAACTTTTAGAATTCTATGAAAGAGTTTCAGGAGCCAGGATGCATGCCAGTT NC_024515_Climacium
TCCTGTGGGCCTTT--GAAGAGCGAGAAAAACTTTTAGAATTCTATGAAAGAGTTTCAGGAGCCAGGATGCATGCCAGTT NC_031212_Brachythecium
TCCTGTGGGCTTTT--GAAGAGCGAGAAAAACTTTTAGAATTCTATGAAAGAGTTTCGGGAGCCAGAATGCATGCCAGTT NC_024520_Atrichum
TTTTGTGGGCTTTT--GAAGAGCGAGAAAAACTTTTAGAATTTTATGAAAGAGTTTCGGGAGCCAGGATGCATGCTAGTT NC_024521_Sphagnum
TCCTGTGGGCCTTT--GAAGAGCGAGAAAAACTTTTAGAATTCTATGAAAGAGTTTCAGGAGCTAGGATGCATGCCAGTT NC_024516_Hypnum
-------------------------------------------------------------------------------- KC 784953 Tetraphis
TCCTGTGGCCCTTTTTGAAGAGCGAGAAAAACTTTCAGAATTCTATGAGAGAGTTTCGGGAGCCAGGATGCATGCCAGTT MF 417767 Mielichhoferia
------------TT--GATTGGCAGGTAAAACTTT---AATCCCAT---------------------------------- NC 024518_Buxbaumia

        2730      2740      2750      2760      2770      2780      2790      2800
....|....|....|....|....|....|....|....|....|....|....|....|....|....|....|....|
ACATACGACCAGGCGGAGTTGCACAAGACATGCCTTTGGGCTTAAGTGAAGATATTTTTCTATTTACACAACAATTTGCT NC_027974_Sanionia
ACATACGACCAGGCGGAGTTGCACAAGACATGCCTTTGGGCTTAAGTGAAGATATTTTTCTATTTACACAACAATTTGCT NC_024515_Climacium
ACATACGACCAGGCGGAGTTGCACAAGACATGCCTTTGGGCTTAAGTGAAGATATTTTTCTATTTACACAACAATTTGCT NC_031212_Brachythecium
ACATACGACCAGGCGGAGTTACACAAGACATGCCTTTGGGCTTAAGTGAAGATATTTTTCTATTTACACAACAATTTGCT NC_024520_Atrichum
ACATACGACCAGGCGGAGTGGCACAAGATATGCCTTTAGGCTTAAGTGAAGATATTTTTCTATTTACACAACAATTTGCT NC_024521_Sphagnum
ACATACGACCAGGCGGAGTTGCACAAGACATGCCTTTGGGCTTAAGTGAAGATATTTTTCTATTTACACAACAATTTGCT NC_024516_Hypnum
-------------------------------------------------------------------------------- KC 784953 Tetraphis
ACATACGACCAGGCGGATTTGCACAAGACATACCTTTGGGCTTAAGTGAAGATATTTTTCTATTTACACAACAATTTGCT MF 417767 Mielichhoferia
------------------------------TGTGGTTGGCTTTAAGCGCAACCAT------------------------- NC 024518_Buxbaumia

        2810      2820      2830      2840      2850      2860      2870      2880
....|....|....|....|....|....|....|....|....|....|....|....|....|....|....|....|
TCTCGTATTGATGAAATAGAAGAAATGTTAACCAACAATCGTATCTGGAAACAACGATTAGTTGATATTGGTACTGTTAC NC_027974_Sanionia
TCTCGTATTGATGAAATAGAAGAAATGTTAACCAACAATCGTATCTGGAAACAACGATTAGTTGATATTGGTACTGTTAC NC_024515_Climacium
TCTCGTATTGATGAAATAGAAGAAATGTTAACCAACAATCGTATCTGGAAACAACGATTAGTTGATATTGGTACTGTTAC NC_031212_Brachythecium
TCTCGTATTGATGAAATCGAAGAAATGTTGACCAACAATCGTATCTGGAAACAACGATTAGTTGATATTGGTACTGTCAC NC_024520_Atrichum
TCTCGTATTGACGAAATAGAAGAAATGTTAACTAACAATCGTATCTGGAAACAACGATTAGTAGATATTGGTACTGTCAC NC_024521_Sphagnum
TCTCGTATTGATGAAATAGAAGAAATGTTAACCAACAATCGTATCTGGAAACAACGATTAGTTGATATTGGTACTGTTAC NC_024516_Hypnum
-------------------------------------------------------------------------------- KC 784953 Tetraphis
TCTCGTATTGACAAAATAAAAGAAATGTTAACCAACAATCGTATCTGAAAACAACGATTAGTTGATATTGGTACTGTTAC MF 417767 Mielichhoferia
-------------------------------------------------------------------------------- NC 024518_Buxbaumia
```

```
        2890      2900      2910      2920      2930      2940      2950      2960
....|....|....|....|....|....|....|....|....|....|....|....|....|....|....|....|
CGCGCAGCAAGCTTTGGATTGGGGATTCAGTGGTGTAATGTTAAGAGGCTCAGGAGTATGCTGGGATTTGCGAAAATCAG NC_027974_Sanionia
CGCACAGCAAGCTTTGGATTGGGGATTCAGTGGTGTAATGTTAAGAGGCTCAGGAGTATGCTGGGATTTGCGAAAATCAG NC_024515_Climacium
CGCACAGCAAGCTTTGGATTGGGGATTCAGTGGTGTAATGTTAAGAGGCTCAGGAGTATGCTGGGATTTGCGAAAATCAG NC_031212_Brachythecium
TGCACAGCAAGCTTTGGATTGGGGATTCAGTGGTGTAATGTTAAGAGGCTCAGGAGTATGCTGGGATTTGCGAAAATCAG NC_024520_Atrichum
TGCACAGCAAGCTTTGGATTGGGGATTTAGTGGTGTAATGCTAAGAGGCTCAGGAGTATGTTGGGATTTGCGAAAATCAG NC_024521_Sphagnum
CGCACAGCAAGCTTTGGATTGGGGATTCAGTGGTGTAATGTTAAGAGGCTCAGGAGTATGCTGGGATTTGCGAAAATCAG NC_024516_Hypnum
-------------------------------------------------------------------------------- KC 784953 Tetraphis
TGCACAGCAAGCTTTGGATTGGGTATTCAGTGGTGTAATGTTAAGAGGCTCAGGAGTATGCCGGGATTTGCGAAAATAAG MF 417767 Mielichhoferia
--------AGGCTTTCGGTCGA-------------------TAGCATAATCATGAGCATGATATATGTTATGGCGATCA- NC 024518_Buxbaumia

        2970      2980      2990      3000      3010      3020      3030      3040
....|....|....|....|....|....|....|....|....|....|....|....|....|....|....|....|
CACCTTACGATGTGTATAACCAATTGATTTTCGATGTACCCGTAGGTACCAGAGGAGATTGTTATGACCGTTATTGTATT NC_027974_Sanionia
CACCTTACGATGTGTATAACCAATTGATTTTCGATGTACCCGTAGGTACCAGAGGAGATCGTTATGACCGTTATTGTATT NC_024515_Climacium
CACCTTACGATGTGTATAACCAATTGATTTTCGATGTACCCGTAGGTACCAGAGGAGATCGTTATGACCGTTATTGTATT NC_031212_Brachythecium
CACCTTACGATGTTTATAATCAATTGATTTTTGATGTACCTGTAGGTACCAGAGGAGATTGTTATGATCGTTATTGTATT NC_024520_Atrichum
CACCTTACGATGTTTATAAGCAATTGGTTTTTGATGTACCCATAGGTACCAGAGGAGATTGTTATGATCGTTATTGTATT NC_024521_Sphagnum
CACCTTACGATGTGTATAACCAATTGATTTTCGATGTACCCGTAGGTACCAGAGGAGATTGTTATGACCGTTATTGTATT NC_024516_Hypnum
-------------------------------------------------------------------------------- KC 784953 Tetraphis
CACCTTACGATGTTTATAACCAATTGATTTTCGATGTACCCGTAGGTACCAGAGGAGATTGTTATGACCGTTATTGTATT MF 417767 Mielichhoferia
-------------------------------------------------------------------------------- NC 024518_Buxbaumia

        3050      3060      3070      3080      3090      3100      3110      3120
....|....|....|....|....|....|....|....|....|....|....|....|....|....|....|....|
CGTATCGAAGAGATGCGACAAAGTATTCGAATCATTATGCAATGTCTTAATCAAATGCCTAGTGGCATGATTAAAGCGGA NC_027974_Sanionia
CGTATTGAAGAGATGCGACAAAGTATTCGAATCATTATGCAATGTCTTAATCAAATGCCTAGTGGCATGATTAAAGCGGA NC_024515_Climacium
CGTATCGAAGAGATGCGACAAAGTATTCGAATCATTATGCAATGTCTTAATCAAATGCCTAGTGGCATGATTAAAGCGGA NC_031212_Brachythecium
CGTATCGAAGAGATGCGACAAAGTATTCGAATCATTATGCAATGTCTTAATCAAATGCCTAGTGGCATGATTAAAGCGGA NC_024520_Atrichum
CGTATCGAAGAGATGCGACAAAGTATTCAAATCATTATGCAATGTCTTAATCAAATGCCTAGTGGCATGATTAAAGCCGA NC_024521_Sphagnum
CGTATCGAAGAGATGCGACAAAGTATTCGAATCATTATGCAATGTCTTAATCAAATGCCTAGTGGCATGATTAAAGCGGA NC_024516_Hypnum
-------------------------------------------------------------------------------- KC 784953 Tetraphis
CGTATTGAAGAGATGCGACAAAGTATTCGAATCATTATGCAATGTCTTAATCAAATGCCTAGTAGCATGATTAAAGCGGA MF 417767 Mielichhoferia
---------------------------------------------------------------------ATTATCGTAGA NC 024518_Buxbaumia

        3130      3140      3150      3160      3170      3180      3190      3200
....|....|....|....|....|....|....|....|....|....|....|....|....|....|....|....|
TGATCGTAAGCTATGTCCCCCTTCACGATCTCAAATGAAACAATCCATGGAATCTTTAATTCATCATTCCAAACTTTATA NC_027974_Sanionia
TGATCGTAAGCTATGTCCCCCTTCACGATCTCAAATGAAACAATCCATGGAATCTTTAATTCATCATTTCAAACTTTATA NC_024515_Climacium
TGATCGTAAGCTATGTCCCCCTTCACGATCTCAAATGAAACAATCCATGGAATCTTTAATTCATCATTTCAAACTTTATA NC_031212_Brachythecium
TGATCGTAAGCTATGTCCTCCCTCACGATCTCAAATGAAACAATCCATGGAATCTTTAATTCACCATTTTAAACTTTATA NC_024520_Atrichum
TGATCGTAAGCTATGTCCTCCGTCACGATCCCAAATGAAACAATCCATGGAATCTTTAATTCACCATTTTAAACTTTATA NC_024521_Sphagnum
TGATCGTAAGCTATGTCCCCCTTCACGATCTCAAATGAAACAATCCATGGAATCTTTAATTCATCATTTCAAACTTTATA NC_024516_Hypnum
-------------------------------------------------------------------------------- KC 784953 Tetraphis
TGATCGTAAGCTATGTCCTCCCTCACGATCTCAAATGAAACAATCCATGGAATCTTTAATTCACCATTTTAAACTTTATA MF 417767 Mielichhoferia
TGATTGATGGCGCTG----------------------------------------------------------------- NC 024518_Buxbaumia

        3210      3220      3230      3240      3250      3260      3270      3280
....|....|....|....|....|....|....|....|....|....|....|....|....|....|....|....|
CAGAAGGTTTTTCTGTACCAGCTTCTTCTACCTATACTGCAGTAGAGGCACCTAAGGGGGAATTTGGTGTGTTTTTAGTC NC_027974_Sanionia
CAGAAGGTTTTTCTGTACCAGCTTCTTCTACCTATACTGCAGTAGAGGCACCTAAGGGAGAATTTGGTGTGTTTTTAGTC NC_024515_Climacium
CAGAAGGTTTTTCTGTACCAGCTTCTTCTACCTATACTGCAGTAGAGGCACCTAAGGGAGAATTTGGTGTGTTTTTAGTC NC_031212_Brachythecium
CAGAAGGTTTTTCTGTACCAGTTTCCTCTACTTATACCGCAGTAGAAGCACCTAAAGGAGAATTTGGTGTGTTTTTAGTC NC_024520_Atrichum
CAGAAGGTTTTTCTATACCAGCTTCTTCTACCTATACCGCAGTAGAAGCACCTAAAGGAGAATTTGGTGTGTTTTTAGTC NC_024521_Sphagnum
CAGAAGGTTTTTCTGTACCAGCTTCTTCTACCTATACTGCAGTAGAGGCACCTAAGGGAGAATTTGGTGTGTTTTTAGTC NC_024516_Hypnum
-------------------------------------------------------------------------------- KC 784953 Tetraphis
CAGAAGGTTTTTCTGTACCAGCTTCTTTTACCTATACTGCAGTAGAAGCACCTAAGGGATAATTTGGTGTGTTTTTAGTC MF 417767 Mielichhoferia
--------------------------------------ACAGTAGAGGAA--------------------GTTTACATTC NC 024518_Buxbaumia

        3290      3300      3310      3320      3330      3340      3350      3360
....|....|....|....|....|....|....|....|....|....|....|....|....|....|....|....|
AGTAATGGAACCAATCGTCCTTATCGTTGTAAAATAAGAGCACCTGGTTTTGCTCATCTACAAGGGCTTGATTTTATGTC NC_027974_Sanionia
AGTAATGGAACCAATCGTCCTTATCGTTGTAAAATAAGAGCACCTGGTTTTGCTCATCTACAAGGGCTTGATTTTATGTC NC_024515_Climacium
AGTAATGGAACCAATCGTCCTTATCGTTGTAAAATAAGAGCACCTGGTTTTGCTCATCTACAAGGGCTTGATTTTATGTC NC_031212_Brachythecium
AGTAATGGAACCAATCGTCCTTATCGTTGTAAAATAAGAGCACCTGGTTTTGCTCATTTACAAGGGCTCGATTTTATGTC NC_024520_Atrichum
AGTAATGGAACCAATCGTCCTTATCGTCGTAAAATAAGAGCACCTGGTTTTGCTCATTTACAAGGACTCGATTTTATGTC NC_024521_Sphagnum
AGTAATGGAACCAATCGTCCTTATCGTTGTAAAATAAGAGCACCTGGTTTTGCTCATCTACAAGGGCTTGATTTTATGTC NC_024516_Hypnum
-------------------------------------------------------------------------------- KC 784953 Tetraphis
AGTAATGGAACCAATCGTCCTTATCATTGTAAAATAAGAGCACCTGGTTTTGCTCATTTACAAGGGTTTGATTTTATGTT MF 417767 Mielichhoferia
AGT----------------------------------------------------------------------------- NC 024518_Buxbaumia
```

```
        3370      3380      3390      3400      3410      3420      3430
....|....|....|....|....|....|....|....|....|....|....|....|....|....|....|.
CAAACATCACATGCTATCAGATGTTGTTACCATAATTGGTACTCAAGATATTGTTTTTGGAGAGGTAGATAGATAG     NC_027974_Sanionia
CAAACATCACATGCTATCAGATGTTGTTACCATAATTGGTACTCAAGATATTGTTTTTGGAGAGGTAGATAGATAG     NC_024515_Climacium
CAAACATCACATGCTATCAGATGTTGTTACCATAATTGGTACTCAAGATATTGTTTTTGGAGAGGTAGATAGATAG     NC_031212_Brachythecium
CAAACATCACATGCTATCAGATGTTGTTACCATAATTGGTACTCAAGATATTGTTTTTGGAGAGGTAGATAGATAG     NC_024520_Atrichum
TAAACATCATATGCTAGCAGATGTTGTCACCATCATTGGTACTCAAGATATTGTTTTTGGAGAGGTAGATAGATAG     NC_024521_Sphagnum
CAAACATCACATGCTATCAGATGTTGTTACCATAATTGGTACTCAAGATATTGTTTTTGGAGAGGTAGATAGATAG     NC_024516_Hypnum
----------------------------------------------------------------------------     KC 784953 Tetraphis
CAAACATCACATGCTATCAGATGTTGTTACCATAATTGGTATTCAAGATATTGTTTTTGGAGAGGTAGATAGATAG     MF 417767 Mielichhoferia
-----------------------TTTTCGCTGTCATTGATACTTCAAATG--------------------------     NC 024518_Buxbaumia
```
