# Supplementary material for: Complete mitochondrial genome sequence of the “copper moss” Mielichhoferia elongata reveals independent nad7 gene functionality loss
Source: PeerJ. 2018 Feb 2;6:e4350. doi: 10.7717/peerj.4350 (PMC5798402; doi:10.7717/peerj.4350)
Supplement: Supplemental Information 4 [file peerj-06-4350-s004.docx]

Tblast results

Query: Bartramia pomiformis exon 2

RGTEKLIEYKTYLQALPYFDRL

\\\\\\\\\\\\\\\\\\\\\\\\\\\\\\\\\\\\\\\\\\\\\\\\\\

*Tetraphis pellucida* mitochondrion, complete genome

Sequence ID: [KC784953.1](https://www.ncbi.nlm.nih.gov/nucleotide/KC784953?report=genbank&log$=nuclalign&blast_rank=1&RID=2VKS0UF0015)Length: 107736Number of Matches: 1

Range 1: 93554 to 93616[GenBank](https://www.ncbi.nlm.nih.gov/nucleotide/KC784953?report=genbank&log$=nuclalign&blast_rank=1&RID=2VKS0UF0015&from=93554&to=93616)[Graphics](https://www.ncbi.nlm.nih.gov/nuccore/KC784953?report=graph&rid=2VKS0UF0015%5bKC784953%5d&tracks=%5bkey:sequence_track,name:Sequence,display_name:Sequence,id:STD1,category:Sequence,annots:Sequence,ShowLabel:true%5d%5bkey:gene_model_track,CDSProductFeats:false%5d%5bkey:alignment_track,name:other%20alignments,annots:NG%20Alignments|Refseq%20Alignments|Gnomon%20Alignments|Unnamed,shown:false%5d&v=93551:93619&appname=ncbiblast&link_loc=fromHSP)Next MatchPrevious Match

| Alignment statistics for match #1 | | | | | | |
| --- | --- | --- | --- | --- | --- | --- |
| **Score** | **Expect** | **Method** | **Identities** | **Positives** | **Gaps** | **Frame** |
| 46.2 bits(108) | 4e-11 | Compositional matrix adjust. | 21/21(100%) | 21/21(100%) | 0/21(0%) | +2 |

Query 1 RGTEKLIEYKTYLQALPYFDR 21

RGTEKLIEYKTYLQALPYFDR

Sbjct 93554 RGTEKLIEYKTYLQALPYFDR 93616

\\\\\\\\\\\\\\\\\\\\\\\\\\\\\\\\\\\\\\\\\\\\\\\\\\\

*Brachythecium rivulare* mitochondrion, complete genome

Sequence ID: [KR732319.1](https://www.ncbi.nlm.nih.gov/nucleotide/KR732319?report=genbank&log$=nuclalign&blast_rank=1&RID=2VN2J98V014)Length: 104460Number of Matches: 1

Range 1: 89284 to 89349[GenBank](https://www.ncbi.nlm.nih.gov/nucleotide/KR732319?report=genbank&log$=nuclalign&blast_rank=1&RID=2VN2J98V014&from=89284&to=89349)[Graphics](https://www.ncbi.nlm.nih.gov/nuccore/KR732319?report=graph&rid=2VN2J98V014%5bKR732319%5d&tracks=%5bkey:sequence_track,name:Sequence,display_name:Sequence,id:STD1,category:Sequence,annots:Sequence,ShowLabel:true%5d%5bkey:gene_model_track,CDSProductFeats:false%5d%5bkey:alignment_track,name:other%20alignments,annots:NG%20Alignments|Refseq%20Alignments|Gnomon%20Alignments|Unnamed,shown:false%5d&v=89281:89352&appname=ncbiblast&link_loc=fromHSP)Next MatchPrevious Match

| Alignment statistics for match #1 | | | | | | |
| --- | --- | --- | --- | --- | --- | --- |
| **Score** | **Expect** | **Method** | **Identities** | **Positives** | **Gaps** | **Frame** |
| 47.0 bits(110) | 2e-11 | Compositional matrix adjust. | 22/22(100%) | 22/22(100%) | 0/22(0%) | +1 |

Query 1 RGTEKLIEYKTYLQALPYFDRL 22

RGTEKLIEYKTYLQALPYFDRL

Sbjct 89284 RGTEKLIEYKTYLQALPYFDRL 89349

\\\\\\\\\\\\\\\\\\\\\\\\\\\\\\\\\\\\\\\\\\\\\\\\\\\\\

Buxbaumia aphylla mitochondrion, complete genome

Sequence ID: KC784954

No significant similarity found.

++++++++++++++++++++++++++++++++++++++++++++++++++++++++++++++

Query: Bartramia pomiformis exon 3

YVSMMAQEHAYSLAVEKLCNCEVPLRAQYIRVLFCEITRILNHLLALTTH

AMDVGALTPFLWAFEEREKLLEFYERVSGARMHASYIRPGGVAQDMPLGL

SEDIFLFTQQFASRIDEIEEMLTNNRIWKQRLVDIGTVTAQQALDWGFSG

VMLRGSGVCWDLRKSAPYDVYNQLIFDVPVGTRGDCYDRYCIRIEEMRQS

IRIIMQCLNQMPSGMIKADDRKLCPPSRSQMKQSMESLIHHFKLYTEGFS

VPASSTYTAVEAPKGEFGVFLVSNGTNRPYRCKIRAPGFAHLQGLDFMSK

HHMLSDVVTIIGTQDIVFGEVDR

Brachythecium rivulare mitochondrion, complete genome

Sequence ID: [KR732319.1](https://www.ncbi.nlm.nih.gov/nucleotide/KR732319?report=genbank&log$=nuclalign&blast_rank=1&RID=2VTGAEU0014)Length: 104460Number of Matches: 2

Range 1: 90352 to 91320[GenBank](https://www.ncbi.nlm.nih.gov/nucleotide/KR732319?report=genbank&log$=nuclalign&blast_rank=1&RID=2VTGAEU0014&from=90352&to=91320)[Graphics](https://www.ncbi.nlm.nih.gov/nuccore/KR732319?report=graph&rid=2VTGAEU0014%5bKR732319%5d&tracks=%5bkey:sequence_track,name:Sequence,display_name:Sequence,id:STD1,category:Sequence,annots:Sequence,ShowLabel:true%5d%5bkey:gene_model_track,CDSProductFeats:false%5d%5bkey:alignment_track,name:other%20alignments,annots:NG%20Alignments|Refseq%20Alignments|Gnomon%20Alignments|Unnamed,shown:false%5d&v=90304:91368&appname=ncbiblast&link_loc=fromHSP)Next MatchPrevious Match

| **Score** | **Expect** | **Method** | **Identities** | **Positives** | **Gaps** | **Frame** |
| --- | --- | --- | --- | --- | --- | --- |
| 670 bits(1729) | 0.0 | Compositional matrix adjust. | 321/323(99%) | 321/323(99%) | 0/323(0%) | +1 |

Query 1 YVSMMAQEHAYSLAVEKLCNCEVPLRAQYIRVLFCEITRILNHLLALTTHAMDVGALTPF 60

YVSMMAQEHAYSLAVEKLCNCEVPLRAQYIRVLF EITRILNHLLALTTHAMDVGALTPF

Sbjct 90352 YVSMMAQEHAYSLAVEKLCNCEVPLRAQYIRVLFREITRILNHLLALTTHAMDVGALTPF 90531

Query 61 LWAFEEREKLLEFYERVSGARMHASYIRPGGVAQDMPLGLSEDIFLFTQQFASRIDEIEE 120

LWAFEEREKLLEFYERVSGARMHASYIRPGGVAQDMPLGLSEDIFLFTQQFASRIDEIEE

Sbjct 90532 LWAFEEREKLLEFYERVSGARMHASYIRPGGVAQDMPLGLSEDIFLFTQQFASRIDEIEE 90711

Query 121 MLTNNRIWKQRLVDIGTVTAQQALDWGFSGVMLRGSGVCWDLRKSAPYDVYNQLIFDVPV 180

MLTNNRIWKQRLVDIGTVTAQQALDWGFSGVMLRGSGVCWDLRKSAPYDVYNQLIFDVPV

Sbjct 90712 MLTNNRIWKQRLVDIGTVTAQQALDWGFSGVMLRGSGVCWDLRKSAPYDVYNQLIFDVPV 90891

Query 181 GTRGDCYDRYCIRIEEMRQSIRIIMQCLNQMPSGMIKADDRKLCPPSRSQMKQSMESLIH 240

GTRGD YDRYCIRIEEMRQSIRIIMQCLNQMPSGMIKADDRKLCPPSRSQMKQSMESLIH

Sbjct 90892 GTRGDRYDRYCIRIEEMRQSIRIIMQCLNQMPSGMIKADDRKLCPPSRSQMKQSMESLIH 91071

Query 241 HFKLYTEGFSVPASSTYTAVEAPKGEFGVFLVSNGTNRPYRCKIRAPGFAHLQGLDFMSK 300

HFKLYTEGFSVPASSTYTAVEAPKGEFGVFLVSNGTNRPYRCKIRAPGFAHLQGLDFMSK

Sbjct 91072 HFKLYTEGFSVPASSTYTAVEAPKGEFGVFLVSNGTNRPYRCKIRAPGFAHLQGLDFMSK 91251

Query 301 HHMLSDVVTIIGTQDIVFGEVDR 323

HHMLSDVVTIIGTQDIVFGEVDR

Sbjct 91252 HHMLSDVVTIIGTQDIVFGEVDR 91320

Range 2: 48190 to 48243[GenBank](https://www.ncbi.nlm.nih.gov/nucleotide/KR732319?report=genbank&log$=nuclalign&blast_rank=1&RID=2VTGAEU0014&from=48190&to=48243)[Graphics](https://www.ncbi.nlm.nih.gov/nuccore/KR732319?report=graph&rid=2VTGAEU0014%5bKR732319%5d&tracks=%5bkey:sequence_track,name:Sequence,display_name:Sequence,id:STD1,category:Sequence,annots:Sequence,ShowLabel:true%5d%5bkey:gene_model_track,CDSProductFeats:false%5d%5bkey:alignment_track,name:other%20alignments,annots:NG%20Alignments|Refseq%20Alignments|Gnomon%20Alignments|Unnamed,shown:false%5d&v=48188:48245&appname=ncbiblast&link_loc=fromHSP)Next MatchPrevious Match[First Match](https://blast.ncbi.nlm.nih.gov/Blast.cgi#hsp940528309_1)

| Alignment statistics for match #2 | | | | | | |
| --- | --- | --- | --- | --- | --- | --- |
| **Score** | **Expect** | **Method** | **Identities** | **Positives** | **Gaps** | **Frame** |
| 20.0 bits(40) | 8.8 | Compositional matrix adjust. | 8/18(44%) | 11/18(61%) | 0/18(0%) | -1 |

Query 234 SMESLIHHFKLYTEGFSV 251

+M L+H F T+GF V

Sbjct 48243 NMAVLVHKFSTNTQGFHV 48190

\\\\\\\\\\\\\\\\\\\\\\\\\\\\\\\\\\\\\\

*Buxbaumia aphylla* mitochondrion, complete genome

*Tetraphis pellucida* mitochondrion, complete genome

No significant similiarity found

| **Search Parameters** | |
| --- | --- |
| Program | tblastn |
| Word size | 6 |
| Expect value | 10 |
| Hitlist size | 100 |
| Gapcosts | 11,1 |
| Matrix | BLOSUM62 |
| Low Complexity Filter | Yes |
| Filter string | L; |
| Genetic Code | 1 |
| Window Size | 40 |
| Threshold | 21 |
| Composition-based stats | 2 |
